# Implementing trust in non-small cell lung cancer diagnosis with a conformalized uncertainty-aware AI framework

**DOI:** 10.1038/s41551-026-01694-8

**Published:** 2026-06-23

**Authors:** Xiaoge Zhang, Tao Wang, Chao Yan, Fedaa Najdawi, Kai Zhou, Yuan Ma, Yiu-ming Cheung, Maximus C. F. Yeung, Bradley A. Malin

**Affiliations:** 1 Department of Industrial and Systems Engineering, State Key Laboratory of Ultra-precision Machining Technology, and Research Institute for Artificial Intelligence of Things, The Hong Kong Polytechnic University, Hong Kong, China.; 2 The Hong Kong Polytechnic University Shenzhen Research Institute, Shenzhen, China.; 3 Department of Biomedical Informatics, Vanderbilt University Medical Center, Nashville, TN, USA.; 4 Department of Pathology, Microbiology and Immunology, Vanderbilt University Medical Center, Nashville, TN, USA.; 5 Department of Computing, The Hong Kong Polytechnic University, Hong Kong, China.; 6 Department of Mechanical Engineering and Research Institute for Intelligent Wearable Systems, The Hong Kong Polytechnic University, Hong Kong, China.; 7 Department of Computer Science, Hong Kong Baptist University, Hong Kong, China.; 8 Department of Pathology, School of Clinical Medicine, LKS Faculty of Medicine, The University of Hong Kong, Queen Mary Hospital, Hong Kong, China.; 9 Department of Computer Science, Vanderbilt University, Nashville, TN, USA.; 10 Department of Biostatistics, Vanderbilt University Medical Center, Nashville, TN, USA.; 11 These authors contributed equally: Xiaoge Zhang, Tao Wang, Chao Yan.

## Abstract

Ensuring trustworthiness is fundamental in cancer diagnostics, where a misdiagnosis can have dire consequences. Current pathology AI models lack systematic solutions to address trustworthiness concerns arising from model limitations and data discrepancies between model deployment and development environments. Here we introduce TRUECAM (Trustworthiness-focused, Uncertainty-aware, End-to-end Cancer diagnosis with Model-agnostic capabilities), a framework designed to ensure both data and model trustworthiness for non-small cell lung cancer subtyping with whole-slide images. TRUECAM integrates (1) a spectral-normalized neural Gaussian process for identifying out-of-scope inputs, (2) an ambiguity-guided tile elimination to filter out highly ambiguous regions, addressing data trustworthiness, and (3) conformal prediction to ensure controlled error rates. We systematically evaluated TRUECAM across multiple cancer datasets using both task-specific and foundation models. Computational experiments suggest that models wrapped with TRUECAM consistently outperformed their unwrapped counterparts in classification accuracy, robustness, interpretability, data efficiency and fairness. These findings establish TRUECAM as a versatile framework for the responsible deployment of pathology AI in real-world settings.

In digital pathology, artificial intelligence (AI) has shown strong potential for decision support across a wide spectrum of clinical tasks, from differentiating between various types of cancer to detecting subtle histopathological changes that may predict disease progression^1–5^. However, the reliability of medical AI models can be non-trivially compromised by their inherent limitations, such as weak ability in measuring uncertainty and controlling error rates. These issues are further compounded by data discrepancies between model development and deployment environments, including variations in patient demographics, disease characteristics, data acquisition techniques, staining protocols and clinical practices. Addressing these challenges is essential to ensure model reliability, uphold patient safety, and establish trustworthiness in AI systems for medical applications^6,7^.

Rather than merely training AI models to output the most likely prediction–a practice that often lacks context and can lead to inaccurate interpretations–researchers have sought to quantify the confidence associated with AI-generated results. This additional layer of insight enables users to make more informed decisions about when to accept its results^8–10^. Multiple uncertainty quantification (UQ) approaches have been recently integrated into AI development to characterize confidence^11–14^. For instance, Olsson et al.^15^ developed a conformalized deep convolutional neural network ensemble to generate all likely cancer diagnoses in biopsies and demonstrated the value of uncertainty estimates in flagging classifications with insufficient confidence. In addition, Dolezal et al.^16^ applied Monte Carlo dropout to quantify classification uncertainty, facilitating the recognition of high-versus low-confidence outputs to address domain shifts. Most recently, Sun et al.^17^ introduced TISSUE, a general framework for estimating uncertainty in spatial gene expression predictions that works by creating well-calibrated prediction intervals.

Despite advances in quantifying uncertainty, the current collection of approaches do not adequately align with essential properties needed for safeguarding practical utilization of pathology AI, and medical AI more broadly^18^. First, an ideal UQ approach should consistently characterize a model’s confidence across varying levels of data risk. Beyond capturing uncertainty for in-domain (In-D) data, it should reliably identify out-of-domain (OOD) inputs, address potential distribution shifts between data used during model development and data encountered after deployment, and recognize regions within a slide that could adversely impact inference. Second, the claimed confidence interval (for example, a set of likely cancer subtypes) or the error rate established for real-world inference must closely align with the observed outcomes. Although existing methods, such as Platt scaling^19–21^ and temperature scaling^22,23^, seek to improve calibration, they neither ensure consistency between expected and observed confidence nor objectively represent the model’s confidence. These methods lack the ability to abstain from inputs that are difficult to infer accurately, a practice widely advocated for reliable medical AI with a human in the loop^7^. Third, when estimating uncertainty, an effective UQ approach should enhance interpretability without adding substantial computational overhead beyond its deterministic counterpart (that is, the original model without UQ). This is essential for medical imaging applications, where strict time constraints and resource limitations demand rapid and efficient processing without compromising reliability. Most UQ approaches rely on ensemble-based estimations, which require a large number of repeated inferences to be made using the same pathology AI models to estimate uncertainty for each patch (or tile) in a single slide. Given that these models typically contain millions of parameters, the resulting computational demand for generating uncertainty and interpretability can become prohibitively large, hindering their usability in practice^24^.

In light of these issues, we set out to equip pathology AI with a formal, principled and scalable UQ, as well as demonstrate how it empowers trustworthy subtyping of non-small-cell lung cancers (NSCLC)^25^ using whole-slide images (WSI). We decompose the trustworthiness of medical AI into two constituent parts: (1) data trustworthiness, which ensures that the input data during deployment aligns with the model’s training scope and allows ambiguous slide regions to be excluded, and (2) model trustworthiness, which offers valid confidence intervals with a customizable level of coverage, ensuring the true label is covered in a specified proportion of classifications. To do so, we developed TRUECAM (Fig. 1a), a framework that provides TRustworthiness-focused, Uncertainty-aware, End-to-end CAncer diagnosis with Model-agnostic capabilities. TRUECAM consists of three components designed to simultaneously ensure data and model trustworthiness: (1) a spectral-normalized neural Gaussian process (SNGP) to establish informative data representation and UQ, (2) an elimination of ambiguous tiles (EAT) mechanism for filtering out noisy patches from slides and (3) conformal prediction (CP) to enable a statistically guaranteed error rate. Over two major NSCLC datasets, we conducted a wide range of experiments using a widely adopted specialized model (that is, Inception-v3 (refs. 26,27)) and multiple general-purpose pathology foundation models (that is, UNI^28^, CONCH^29^, Prov-GigaPath^30^ and TITAN^31^). Our results indicate that TRUECAM provides notable improvements in NSCLC subtyping accuracy and often enhances fairness in model performance across demographic groups without explicitly enforcing it into the model training process. Our analysis further shows that TRUECAM is robust to OOD input and distribution shift, delivers more informative confidence intervals, enhances interpretability and substantially reduces the computational inference burden. TRUECAM’s superiority scales within NSCLC subtyping and generalizes beyond lung cancer, as evidenced by its consistently strong performance on a geographically distinct private NSCLC cohort and across five additional non-lung cancer subtyping tasks, including a challenging 46-class pan-cancer slide-level classification setting^31^. Together, these observations position TRUECAM as a general framework that can be integrated with pathology AI models of various sizes, architectures, purposes and complexities to support their responsible applications.

## Results

### An overview of TRUECAM

We designed the TRUECAM framework (Fig. 1a) to wrap around deep learning models of diverse architectures for imaging tasks (Fig. 1b). TRUECAM uses SNGP to perform distance-preserving transformations on the input data and efficiently estimate data uncertainty, enhancing data trustworthiness by enabling OOD detection and data shift control before model inference. Furthermore, CP is applied on top of SNGP to calibrate model predictions (represented as a set of values, that is, prediction set), providing a statistical guarantee on the model’s coverage of true labels. These components contribute complementary functions to assure the trustworthiness of AI for NSCLC subtyping, framed as a binary classification task distinguishing lung adenocarcinoma (LUAD) from lung squamous cell carcinoma (LUSC). Specifically, SNGP establishes a valid foundation for CP to operate. This is important because CP generally requires that data in the deployment environment comes from the same underlying distribution as those in the development environment (that is, data exchangeability)—an assumption that might not hold in the real-world setting, where OOD data and distribution shift are common. Meanwhile, CP calibrates the outputs of SNGP to ensure the statistical validity of model coverage. The distance-preserving data transformation by SNGP facilitates an effective identification of tiles that only add ambiguity to NSCLC subtyping. EAT can then be applied to enhance the supervisory signal for improved model reliability, while considerably reducing the computational load during inference (Fig. 1c). See Methods for the details of TRUECAM.

To assess TRUECAM’s effectiveness, generalizability and scalability, we used a diverse collection of datasets totalling over 20,000 WSIs, including two NSCLC datasets (referred to as TCGA (The Cancer Genome Atlas) and CPTAC (Clinical Proteomic Tumor Analysis Consortium); Fig. 1d) and several additional cancer subtyping or classification datasets, covering both specialized and pathology foundation models (see Methods for details). Our evaluation yielded several notable findings. First, TRUECAM significantly reduced NSCLC subtyping error rates (defined as the fraction of incorrect patient-level classifications) across all models, compared to their original versions, denoted as Deterministic (or D) (Fig. 1e and Extended Data Fig. 1). Defining α as the desired maximum error level (or significance level), applying TRUECAM to Inception-v3 (Incep-TRUECAM) with a target coverage of 1−α=0.95 and 1−α=0.99 reduced the error rate by 72.0% and 93.8%, respectively. These empirical error rates were nearly complementary to the desired coverage levels (that is, approximately 1−α). Second, TRUECAM showed an ability to identify and abstain from reasoning about challenging inputs, allowing such inputs to be deferred to pathologists. As shown in Fig. 1f, with coverage set at 1−α=0.95, Incep-TRUECAM abstained on approximately 15 patients, reducing the number of misclassified patients from 14 to fewer than 4. Third, the foundation model-based classifiers showed more promising results than Inception-v3, with inherently lower error rates and fewer abstentions to achieve the same desired coverage (Fig. 1e,f). Fourth, in simulated real-world clinical settings, where slides from non-cancerous lung tissue may be unknowingly submitted for NSCLC subtyping, directly deploying the original versions of the considered models can incorrectly classify non-cancerous slides as LUAD or LUSC. By contrast, TRUECAM reliably detected these OOD inputs before model inference, while maintaining comparable error rates as the scenarios involving only In-D inputs (Fig. 1g).

In the following sections, we systematically evaluate TRUECAM and its core modules using Inception-v3 as the primary example then extend the analysis to multiple foundation models (UNI, CONCH, Prov-GigaPath and TITAN) paired with multiple instance learning (MIL) mechanisms (ABMIL (attention-based multiple instance learning), CLAM and TransMIL) where applicable.

### Integration of distance-aware uncertainty estimation improves NSCLC subtyping performance

We trained three Inception-v3-based models to discriminate between LUSC and LUAD: (1) a deterministic model without UQ, referred to as Deterministic, (2) a Monte Carlo Dropout-based model (referred to as MC Dropout)^16^ and (3) a model that uses SNGP for UQ (referred to as SNGP). We deactivated TRUECAM’s CP functionality and EAT to assess the effectiveness of SNGP. We used TCGA for model training and internal validation and used CPTAC as an independent external validation dataset to evaluate model generalizability.

At the tile level, SNGP consistently outperformed Deterministic and MC Dropout on both TCGA and CPTAC in terms of accuracy and the area under receiver operator curves (AUROC) (*P* < 0.05; Fig. 2a,b). At the patient level, performance gains were more pronounced. On TCGA, Deterministic and MC Dropout performed similarly, with Deterministic averaging an accuracy of 0.843 and AUROC of 0.926, whereas SNGP significantly exceeded both with an accuracy of 0.875 and AUROC of 0.950 (*P* < 0.001; Fig. 2c). On CPTAC, SNGP outperformed MC Dropout and Deterministic by 4.9% and 7.2% in accuracy, and 4.5% and 5.7% in AUROC, respectively (*P* < 0.001; Fig. 2d). SNGP also outperformed variational Bayesian last layer (VBLL)^32^—a state-of-the-art neural network component for UQ—across both datasets (*P* < 0.001) (Supplementary Table 8).

### Conformalized SNGP establishes statistical coverage guarantee while enhancing NSCLC subtyping efficiency

We enabled CP in TRUECAM and assessed its effectiveness in calibrating SNGP-based NSCLC subtyping by generating a prediction set for each patient. A set size of 2 indicates that the model lacks sufficient confidence to definitively classify input data as either LUAD or LUSC, effectively resulting in abstention. Two broadly used measures in the literature, validity and efficiency^33–35^, were evaluated. A model is considered valid at error level α if its coverage (the fraction of prediction sets containing the true subtype) is at least 1−α. Efficiency is determined by the prediction set size: among valid models, those producing smaller sets are regarded as more efficient.

All three models achieved the validity requirements at both the tile and patient levels on TCGA and CPTAC for distinct error levels (Supplementary Tables 9 and 10). For all considered coverage, con- formalized SNGP consistently produced smaller prediction sets than conformalized MC Dropout and Deterministic on both datasets in a statistically significant manner (Fig. 2e-h). At α=0.05, conformalized SNGP yielded 41,570 more tiles with a single-subtype prediction (set size of 1) than conformalized MC Dropout, representing a 31.4% increase (Supplementary Tables 9 and 10). As expected, reducing *α* resulted in larger prediction sets and increased abstention (Fig. 2e-h).

We further stratified patient-level predictions into three categories: (1) single and correct, (2) single but incorrect and (3) uncertain between subtypes (abstention). Across all α, conformalized MC Dropout showed similar patient-level distributions across these categories as Deterministic (Fig. 2i,j). By contrast, conformalized SNGP yielded more single and correct classifications with a comparable number of single but incorrect classifications (Fig. 2i,j). At α=0.01, it assigned single correct subtype to 7 more patients from TCGA (23.4% increase) (Fig. 2i) and around 30 more patients from CPTAC (83.7% increase) than conformalized MC Dropout (Fig. 2j). The gain in correct singles of conformalized SNGP was mirrored by fewer abstentions, underscoring TRUECAM’s practical utility.

Next, we examined TRUECAM’s capability of managing errors when yielding single subtypes. We define the definitive-answer error rate (or DA error rate) as the proportion of patients with a single but incorrect subtype designation among all patients receiving a single-subtype prediction. We observed that before activating CP, TRUECAM already achieved the lowest error rate on both TCGA and CPTAC (Fig. 2k,l). Allowing abstention via CP further reduced DA error rates for all models, with the rates decreasing monotonically as α decreased. At α=0.01, CP reduced SNGP’s DA error rate on TCGA to 1.9% (an 84.8% reduction compared to SNGP alone), improving from roughly 1 error per 8 patients to 1 per 100. In addition, across all coverages, conformalized SNGP had the lowest DA error rates, indicating that TRUECAM delivered more accurate classifications than competing models.

### EAT concurrently augments classification and CP performance in NSCLC subtyping

In clinical inference using a WSI, not all regions provide diagnostic value (for example, areas with normal, non-cancerous cells). Nevertheless, the current practice in digital pathology typically uses all tiles—often hundreds to tens of thousands per WSI paired with coarse-grained slide-level labels for model training^36–40^. Such a weakly supervised learning process inherently introduces noise, as normal tiles might be mislabelled as tumours^41^, forcing models to learn from irrelevant or non-informative regions and thus diluting the signal necessary for accurate and efficient cancer subtyping.

To filter non-informative tiles, we applied *k*-means clustering to SNGP-derived tile representations from the TCGA training set. Based on the validation data performance, we determined *k* = 3 as the optimal number of clusters. Tiles in the cluster with the highest subtype heterogeneity (quantified by cluster label assignment entropy; Methods) were targeted for elimination. Among the three clusters, one cluster contained mostly tiles from WSIs with the true label of LUAD, another cluster contained mostly tiles with the true label of LUSC, and the cluster targeted for elimination was not dominated by either subtype (Fig. 3a). Using *t*-distributed stochastic neighbour embedding (*t*-SNE) visualization (Fig. 3b), we observed that the clustering pattern consistently aligned with the label distribution depicted in Fig. 3a.

We then quantified tile ambiguity using label assignment entropy and found statistically significantly higher ambiguity in the cluster lacking clear label dominance (referred to as ambiguous cluster) than in the other two clusters (Fig. 3c). This suggests that the tiles in the ambiguous cluster may only add ambiguity to NSCLC subtyping when aggregating tile-level classifications. We therefore hypothesized that eliminating ambiguous tiles via EAT would strengthen the supervisory signals for model training and improve subtyping performance. To test this, we removed the training tiles from the ambiguous cluster (66.7% of all training tiles) and trained a new model SNGP-EAT (Fig. 1c). During inference, tiles from a new slide with their latent representations falling within the ambiguous cluster were excluded from the subtyping task.

We further established a baseline, SNGP-RE (RE, random elimination), which eliminates an equal number of tiles as SNGP-EAT but uniformly at random. SNGP-EAT improved patient-level accuracy over SNGP by 2.83% on TCGA and 8.05% on CPTAC (*P* < 0.001; Fig. 3d and Supplementary Table 8), whereas SNGP-RE consistently underperformed compared with both SNGP and SNGP-EAT in terms of accuracy and AUROC (Extended Data Fig. 2a-d). Moreover, EAT improved CP efficiency by producing smaller prediction sets (Extended Data Fig. 2e-h and Supplementary Tables 9 and 10) and more single, correct classifications (Fig. 3e,f), except for the TCGA setting with α=0.01, while yielding a comparable number of single but incorrect classifications to the models without EAT. Consistently, DA error rate comparisons also favoured SNGP-EAT (Extended Data Fig. 2i,j and Supplementary Tables 11 and 12).

### TRUECAM achieves fairer NSCLC diagnosis compared to other methods

We investigated how TRUECAM affects fairness by comparing it to several baseline models. We first evaluated the classification performance gap without CP, defined as the maximum difference in accuracy among patient subgroups stratified by race and sex (Supplementary Table 1). We then assessed fairness in terms of average CP set size across these subgroups, where a larger difference in set sizes between two subgroups suggests greater uncertainty for one subgroup than the other.

SNGP and SNGP-EAT consistently showed higher overall classification accuracy than Deterministic and MC Dropout, while also improving fairness in accuracy across racial and sexual subgroups when Deterministic’s fairness gap was relatively high or preserving fairness when the gap was relatively low (Fig. 4a-d). SNGP-EAT attained the highest overall accuracy on both internal and external datasets and the smallest accuracy gaps across racial subgroups, reducing these gaps by 38.1% and 78.3% in TCGA and CPTAC, respectively, compared to Deterministic (Supplementary Tables 15 and 16). By contrast, randomly removing tiles through SNGP-RE did not substantially improve either accuracy or fairness, again highlighting the benefit of EAT.

The advantages of conformalized SNGP and SNGP-EAT were even more pronounced in terms of fairness in average CP set size (Fig. 4e-h). Remarkably, conformalized SNGP-EAT (that is, TRUECAM) consistently achieved the smallest set size gaps across sexual and racial subgroups. For example, at 1−α=0.90, compared to conformalized Deterministic, conformalized SNGP-EAT reduced the set size gap in race by 42.2% on TCGA and 27.4% on CPTAC (Fig. 4f,h and Supplementary Tables 17 and 18) and reduced the set size gap in sex by 51.6% and 73.0%, respectively, approaching a near-perfect value (Fig. 4e,g and Supplementary Tables 19 and 20).

### TRUECAM enables effective OOD detection and distribution shift control

In pathology AI deployment, WSIs that differ from the model’s training data in cohorts, tissue types, devices and other factors are prevalent yet difficult to detect. For CP-equipped models, the claimed model coverage may no longer hold under such conditions, which undermines reliability and poses risks to patient safety. We now show that TRUECAM can mitigate this issue by detecting data outside of the model’s scope (that is, OOD data).

We combined a constructed OOD dataset with an In-D dataset (comprising the TCGA calibration and testing datasets) and defined two OOD measures: (1) an uncertainty-based OOD score, which aggregates tile-level uncertainty from the model’s UQ module (for example, Gaussian layer for SNGP/SNGP-EAT, stochastic dropout layer for MC Dropout), and (2) a probability-based OOD score, which aggregates tile-level classification probabilities as $1-\frac{1}{N}\sum_{i=1}^{N}\max_{k}\;p\left({\hat{y}}_{k}|x_{i}\right)$, where $p\left({\hat{y}}_{k}|x_{i}\right)$ denotes the probability of tile i being classified as label ${\hat{y}}_{k}$. We observed that the probability-based OOD score from SNGP-EAT performed the best in separating In-D and OOD WSIs, achieving the highest AUROC (0.960) and outperforming its uncertainty-based counterpart (0.910) (Fig. 5a). At a precision of 0.9, SNGP-EAT reached a true positive rate (TPR) of 0.919, exceeding that of SNGP by 5.8% (Fig. 5b), whereas Deterministic failed to reach 0.9 precision. Subsequent OOD analyses for Inception-v3 used the best-performing OOD measure for each model.

We further examined model validity by assessing how removing OOD inputs identified by TRUECAM affects empirical coverage. When we randomly sampled an equal number of OOD cases to match the In-D dataset size, SNGP-EAT’s empirical coverage increased to 0.931 at a TPR of 0.98 (Fig. 5c). However, without OOD detection, the empirical coverage remained at 0.477, indicating a failure in the model’s reliability. The remaining gap from the desired coverage of 0.95 (α=0.05) was largely due to Inception-v3’s limited ability to distinguish OOD from In-D data. As shown later, applying TRUECAM to foundation models effectively closed this gap. However, even with the best-performing model and OOD score, identifying 98.0% OOD inputs required misclassifying 13.1% of In-D data as OOD (Fig. 5c), excluding them from model inference. Furthermore, the OOD threshold chosen to maximize TPR in testing might not guarantee comparable performance in deployment, where unexpected OOD data may fall beyond the calibrated range, compromising model coverage and trustworthiness. To address this, TRUECAM pairs conformal risk control (CRC) with OOD detection to preserve reliable coverage even when some OOD data go undetected. CRC dynamically adjusts model outputs based on residual OOD risk, thereby maintaining valid empirical coverage (Methods). We then evaluated TRUECAM across varying OOD-to-In-D ratios and found that combining OOD detection with CRC yielded a robust empirical coverage that closely matched the 0.95 target (α=0.05) at all ratios (Fig. 5d). As OOD proportion increased and more OOD data were misclassified as In-D, the prediction set size increased accordingly (that is, more abstention) to maintain a valid empirical coverage. By contrast, removing either CRC or OOD detection substantially degraded empirical coverage, with the decline worsening as the OOD fraction grew.

In addition, we investigated whether applying an inspection step (as above) before deploying a trained model on a new population (CPTAC in our scenario) could mitigate performance degradation (observed in Fig. 2a-d) due to distribution shift. We call this mechanism distribution shift control (DSC), designed to support safe model transfer, especially where fine-tuning is not feasible. We observed that excluding the CPTAC data at an OOD score threshold corresponding to a false positive rate (FPR) of 0.2 enhanced NSCLC subtyping performance (Fig. 5e,f) and CP efficiency by reducing set sizes across all settings (Fig. 5i,j). A sensitivity analysis showed that using an OOD score threshold corresponding to a higher FPR consistently enhanced NSCLC subtyping accuracy (Fig. 5g), AUROC (Fig. 5h), average set size (Extended Data Fig. 3a) and DA error rate (Extended Data Fig. 3b). Across all metrics, SNGP-EAT substantially outperformed competing models (Supplementary Tables 23 and 24).

### TRUECAM’s benefits extend to digital pathology foundation models

Beyond specialized models that perform tile-level inference and aggregation, TRUECAM also seamlessly integrates with general-purpose pathology foundation models to make slide-level inference directly (Fig. 1b). Specifically, TRUECAM uses a foundation model’s pretrained encoder to extract tile representations, assigns ambiguity scores to discard confusing tiles, and feeds the remaining tiles into either an MIL mechanism for tile-level models or a slide encoder for slide-level models.

We now demonstrate that TRUECAM established both data and model trustworthiness for NSCLC subtyping using pathology foundation models. First, without CP, UNI-TRUECAM and CONCH-TRUECAM-each discarding 60.0% of tiles per slide using the label assignment entropy-based ambiguity measure-improved patient-level subtyping accuracy (Fig. 6a), reducing the error rate by 10.04% and 8.48%, respectively, relative to their deterministic counterparts (Fig. 6i,j). These gains, reflecting the combined effect of SNGP and EAT, were consistent for other MIL architectures (Supplementary Table 27). Furthermore, Prov-GigaPath and TITAN preserved their subtyping performance using only 40.0% of the total tiles under TRUECAM (Supplementary Table 27). Second, with CP activated, TRUECAM achieved empirical coverage closely matching the desired level (Fig. 6b), whereas the original ABMIL-based UNI-D and CONCH-D reached only 0.931 and 0.921 and generally lacked the flexibility to adjust to customized error levels. Third, compared with conformalized UNI-D and CONCH-D (without SNGP-EAT), TRUECAM achieved smaller average prediction set sizes (Fig. 6c) and more single, correct classifications (Fig. 6d). Fourth, TRUECAM yielded fairer or comparable classification performance across subgroups for both foundation model-based ABMIL set-ups compared to scenarios without TRUECAM in most cases (Extended Data Figs. 5 and 6).

In the foundation model setting, TRUECAM provided a stronger OOD detection capability than in the specialized model setting. Using the same OOD study design, we observed that the uncertainty-based OOD score (AUROC of 0.983 for UNI-TRUECAM and 0.993 for CONCH-TRUECAM) consistently outperformed the probability-based score (AUROC of 0.967 for UNI-TRUECAM and 0.854 for CONCH-TRUECAM) in distinguishing between In-D and OOD WSIs (Fig. 6e and Supplementary Table 21). Thus, the uncertainty-based OOD score was adopted for OOD detection. Without OOD detection, UNI-TRUECAM and CONCH-TRUECAM showed empirical coverage far below the desired level (Fig. 6f); however, adjusting the OOD threshold to approach a near-perfect TPR restored model coverage. To manage undetected OOD inputs over varying OOD-to-In-D ratios, TRUECAM integrates CRC with OOD detection to preserve empirical coverage that aligned closely with the desired level across a broad ratio range for both UNI-TRUECAM and CONCH-TRUECAM (Fig. 6g,h). By contrast, neglecting either CRC or OOD detection led to worse empirical coverage.

We further compared ambiguity-based tile elimination in TRUECAM’s EAT with uniform random elimination and analysed the sensitivity of model accuracy to the tile retention rate. We observed that ambiguity-based tile elimination outperformed random tile elimination across a wide range of retaining rates for both foundation model-based ABMIL settings (Fig. 6k,l). Remarkably, even at a 0.1% tile retention rate, ambiguity-based EAT did not compromise classification accuracy, whereas random tile elimination at a comparable retention rate caused substantial accuracy loss. These results indicate that the ambiguity-based EAT effectively preserves the most informative tiles for NSCLC subtyping.

### TRUECAM delivers efficient interpretation and inference

Compared to traditional approaches that rely solely on attention for interpretation, TRUECAM offers a diagnostic basis for model inference through two sequential interpretability layers informed by its UQ module: (1) tile-level ambiguity, which assesses each tile’s potential to introduce confusion and helps eliminate corresponding regions, and (2) a global attention map that shows the reliance of slide-level inference on the remaining tiles after EAT. As a result, as ambiguous tiles are excluded by the first layer, interpretability is grounded in tiles showing high or low attention, all characterized by low ambiguity.

To validate the diagnosis basis of TRUECAM, we examined two WSIs from the TCGA testing cohort: one LUAD (Extended Data Fig. 7) and one LUSC (Extended Data Fig. 8). A pathologist (F.N.), blinded to all model outputs, annotated tumour regions with clear diagnostic relevance for NSCLC subtyping and assessed a random subset of tiles from representative regions to judge their utility in subtyping. We observed that regions with relatively low ambiguity closely matched the pathologist’s annotations and supported discrimination between LUAD and LUSC using established diagnostic criteria. Specifically, these low-ambiguity regions contained cancer epithelial areas critical for distinguishing NSCLC subtypes. By contrast, high-ambiguity regions lacked distinctive morphological features of either NSCLC subtype. For example, tiles with blue borders in both WSIs contained inflamed non-neoplastic lung parenchyma, areas of stroma, or necrosis, all lacking tumour-specific morphological hallmarks (Extended Data Figs. 7 and 8).

After EAT, the morphological features in regions with high attention weights became the primary basis for the model’s classification. As confirmed by the pathologist, the high-attention regions highlighted in blue were generally consistent with the diagnostic criteria for NSCLC subtyping. Tiles with red borders exemplified this alignment: LUAD-specific regions revealed glandular arrangements characteristic of adenocarcinoma, such as well-formed acinar patterns, while LUSC-specific regions showed intercellular bridges, focal keratinization and dense eosinophilic cytoplasm, all consistent with the morphological features for classification. However, not all regions contributed equally to subtype discrimination. Regions lacking clear indicators for either subtype were recognized by TRUECAM with correspondingly low attention weights. For example, tiles with purple borders included stroma, areas of inflammation, areas of necrosis and poorly differentiated regions, none of which provided clear discriminative clues for LUAD versus LUSC.

To quantitatively compare model interpretability, we introduce attention efficiency, defined as the fraction of high-attention tiles that pathologists also regard as highly relevant for subtyping. This metric captures how precisely a model’s attention highlights diagnostically important regions. TRUECAM achieved consistently higher attention efficiency than traditional attention mechanisms across all three model types (Extended Data Fig. 9a,b).

Beyond enhanced interpretability, TRUECAM also improved inference efficiency relative to traditional and baseline UQ methods. First, Inception-v3 with SNGP (Incep-SNGP) achieved nearly identical tile inference speed as its deterministic counterpart and was 5 times faster than MC Dropout models (Extended Data Fig. 9c). Second, SNGP added negligible computational overhead to foundation models, partially attributed to their inherently slower encoding process. Third, while foundation models achieved lower inference speeds compared to Inception-v3, they delivered superior classification performance (Supplementary Tables 8 and 25). Fourth, EAT substantially boosted slide inference speed by excluding a non-trivial proportion of ambiguous tiles, while simultaneously enhancing classification and CP performance (Extended Data Fig. 9d). This inference efficiency gain positioned TRUECAM closer to practical deployment, particularly in time-sensitive and resource-limited medical settings.

### TRUECAM shows strong scalability and generalizability in NSCLC subtyping and beyond

To assess generalizability in a real-world clinical setting, we evaluated TRUECAM on Queen Mary Hospital (QMH)-NSCLC, a geographically distinct cohort from TCGA and CPTAC. Computational results showed that the TRUECAM-wrapped specialized model substantially outperformed its non-TRUECAM counterpart in both balanced accuracy and weighted F1 score (Supplementary Table 26), while using 38% fewer tiles for inference. Similarly, TRUECAM-wrapped pathology foundation models consistently outperformed their counterparts in 15 out of 16 scenarios (Supplementary Table 29), with an average 40% reduction in tiles used at inference.

We further evaluated TRUECAM on five additional datasets spanning diverse non-lung cancer subtyping tasks that varied by organ site and the number of subtypes. Among them, the TCGA-OncoTree (TCGA-OT) dataset features a slide-level, 46-class pan-cancer classification task^31^, making it a particularly demanding evaluation scenario for testing model scalability; together, all datasets collectively serve to assess generalizability.

In the specialized model setting, TRUECAM improved balanced accuracy and weighted F1 score across all datasets, except for weight F1 score on BRACS-Coarse (Supplementary Table 26), yielding an average 21.6% reduction in balanced error rate and a 5.5% gain in weighted F1 score, along with an average 31.4% reduction in tiles processed at model inference. In the foundation model setting, performance improvements were consistent and independent of the MIL mechanism (Supplementary Tables 30-34). Overall, TRUECAM outperformed the original models in 65 out of 80 scenarios, with an average 6.8% reduction in balanced error rate, 1.2% increase in weighted F1 score and 23.6% reduction in inference cost.

In summary, TRUECAM scales and generalizes effectively to multi-class subtyping tasks, regardless of the underlying model architecture, MIL mechanisms, number of cancer subtypes and task complexity. On top of the elevated model performance with reduced inference cost, TRUECAM also provides a statistically guaranteed error rate through CP, DSC through OOD detection, and fine-grained interpretability, thereby supporting its reliable application in clinical cancer subtyping tasks that demand trustworthiness.

## Discussion

Trustworthiness of pathology AI is often compromised by model limitations in delivering reliable performance and quantifying uncertainty with statistical implication. Moreover, data challenges like noisy tiles and the complexity of model deployment environment, including OOD data and distribution shifts, further undermine trustworthiness of these systems. To address these issues, we developed TRUECAM, a framework designed to seamlessly integrate with diverse model architectures and use cases to enable trustworthy AI-driven cancer subtyping in pathology.

TRUECAM has direct implications for the real-world deployment of pathology AI. Its most immediate benefit is a substantial reduction in the likelihood of incorrect model results. Beyond boosting diagnostic accuracy, TRUECAM redefines how AI can be safely integrated into cancer diagnostic workflow. To manage the risk of incorrect patient-level diagnoses and ensure statistically guaranteed bounds in error rate, TRUECAM adaptively defers uncertain or OOD cases to expert pathologists, fostering a collaborative human-AI decision-making process. This directly tackles a fundamental challenge in pathology AI: inference data may experience distribution shifts, whether due to temporal changes at the same site, inter-site variability, or differences in patient populations or clinical practices, all of which undermine model trustworthiness. By explicitly accounting for these factors, TRUECAM helps ensure that AI functions as a reliable assistant rather than an inflexible tool, complementing clinical workflows while mitigating automation bias. Critically, TRUECAM’s deferral mechanism is proactive, customizable and strategic. It abstains less frequently than other models yet achieves higher accuracy, showing its ability to recognize and act on its own limitations. This confines AI usage within a trusted scope and reduces the cognitive load on pathologists, who can focus their expertise on the most challenging cases^42–44^. By integrating reliability, adaptability and collaboration, TRUECAM represents a transformative step towards making AI a trusted partner in clinical decision-making.

TRUECAM not only constrains diagnostic use to settings where the model is reliable; it also provides a principled pathway to expand that trusted scope. In a human-AI collaborative framework, pathologists can label slides flagged as OOD or abstained cases. These newly labelled data points can then be incorporated into the UQ pipelines, enabling iterative refinement of both the underlying model and its error bound guarantees. This progressive, feedback-driven process allows the model’s domain of validity to grow in a controlled and transparent way, aligning model evolution with real-world clinical use. As trustworthiness becomes central to medical AI, the ability to systematically address and update model limitations becomes critical^45,46^. By enabling pathology AI to adapt and expand their capabilities through iterative feedback and refinement, TRUECAM provides key insights that can guide the next generation of pathology foundation models towards scalable and trustworthy deployment.

While SNGP and EAT are designed to address data trustworthiness and CP (with CRC) to assure model trustworthiness, these components do not function independently but instead provide complementary benefits. SNGP-EAT establishes an environment that preserves data exchangeability for CP to function and ensures that the input data are within the scope of the trained models. Meanwhile, CP guarantees that classifications based on SNGP-EAT are statistically valid in containing the ground truth labels with a user-specified coverage guarantee. Removing either component substantially degrades reliability. For example, omitting SNGP-EAT-based OOD detection led to a drop in empirical coverage to 0.477 (versus a target of 0.95), resulting in 52.3% incorrect patient-level subtyping despite CP being applied (Fig. 5d). Conversely, using SNGP and EAT without CP improved accuracy over baseline (Fig. 3) but could not abstain on difficult cases or provide bounded error rates. The interplay among these components yields a robust framework capable of handling real-world variability in pathology workflows.

EAT, in particular, aligns with the data-centric AI paradigm by exemplifying a ‘less data, better performance’ effect^42–44^. While conceptually related to the top-K max-pooling operator used in MI-Zero^29,47^, EAT goes further by explicitly discarding highly ambiguous tiles and concentrating on high-value diagnostic regions. This produces a feedback loop in which model training is driven by more informative supervision, improving tile-level representations and amplifying both efficiency and accuracy. EAT delivers several tangible benefits. First, it substantially lowers computational burden for slide-level inference. For example, in the specialized model (Inception-v3), EAT reduced the number of tiles by 66% (Fig. 3a), while in the foundation model-based setting, this efficiency gain reached up to 1,000 times without compromising accuracy (Fig. 6k,l). This efficiency lowers barriers to deploying advanced pathology AI models in resource-constrained clinical settings. Second, EAT improves interpretability by aligning the model’s focus with diagnostically relevant areas that closely match pathologist’s annotations (Extended Data Figs. 7 and 8). This alignment makes AI outputs easier to review, supports clinical trust and reduces cognitive burden. Third, from a data annotation standpoint, EAT offers a scalable way to filter low-value tiles, alleviating the need for labour-intensive and costly pixel-level labels and better leveraging scarce expert time. Fourth, EAT promotes fairness by reducing demographic disparities in both classification performance and CP set size. By eliminating ambiguous tiles from each slide, EAT mitigates biases that could arise when pathology models process all tiles indiscriminately, and it does so without requiring explicit fairness constraints during training. Finally, EAT also has far-reaching implications for foundation model pretraining: current self-supervised (for example, DINOv2 (ref. 48)) or vision-language approaches treat all tiles as equally informative, even though many are clinically irrelevant. Using EAT to filter out non-diagnostic tiles during pretraining could focus model learning on clinically meaningful cancerous areas, improving representation quality and reducing training costs.

Collectively, the demonstrated benefits of TRUECAM’s components, both individually and in combination, contribute to a more reliable and trustworthy pathology AI system. By substantially reducing model error rates, controlling OOD inputs and distribution shifts, lowering inference costs, mitigating model failure risks, and proving generalizability and scalability, TRUECAM strengthens the foundation for real-world clinical translation.

Several limitations warrant further investigation. First, we evaluated TRUECAM using one specialized model and four state-of-the-art pathology foundation models that lack conversational capabilities. With the emergence of interactive, language-centred pathology models^49–51^, it is important to adapt TRUECAM to this new paradigm and assess its impact on reliability. Second, although DSC enhanced model performance on an external dataset (CPTAC) relative to a distinct training dataset (TCGA) (Fig. 5e,f,i,j), the root cause of the distribution shift remains unclear. Systematically characterizing these shifts could inform further model refinement. Third, we did not explore TRUECAM in hierarchical tissue subtyping tasks that follow multi-level decision trees from coarse tissue type to fine-grained histologic or molecular subtypes. Managing uncertainty at each decision node will be crucial to preventing error propagation in such settings. Finally, we did not assess TRUECAM in a fully human-in-the-loop clinical environment, particularly when abstentions trigger additional review. Future prospective studies will be essential to evaluate how pathologists use and potentially override TRUECAM’s outputs, and how these interactions affect patient outcomes. Designing effective collaboration strategies will be key before integrating TRUECAM into routine clinical workflows.

## Methods

### Dataset description

#### Dataset overview.

We used two publicly available and one private digital pathology datasets of NSCLC stained with haematoxylin and eosin in the binary lung cancer subtyping task: (1) 941 WSIs from TCGA, comprising 467 LUAD and 474 LUSC cases, (2) 1,306 WSIs from the CPTAC, comprising 644 LUAD and 662 LUSC cases, and (3) 3,123 WSIs from Queen Mary Hospital, comprising 2,645 LUAD and 478 LUSC cases. For simplicity, we refer to these three datasets as TCGA, CPTAC and QMH-NSCLC, respectively. QMH-NSCLC was collected under the approval of Institutional Review Board of The University of Hong Kong/Hospital Authority Hong Kong West Cluster (HKU/HA HKW IRB) (UW22–168) with a waiver of informed consent due to the retrospective nature of the study and the use of de-identified data.

Supplementary Tables 1 and 2 summarize the basic characteristics of these three datasets. In the TCGA dataset, each patient is represented by a single slide, whereas in the CPTAC and QMH-NSCLC datasets, each patient is associated with an average of 3.1 and 2.3 slides, respectively. To evaluate TRUECAM’s ability in detecting OOD inputs, we constructed a clinically meaningful dataset consisting of 218 cancer-adjacent normal tissues (defined as solid tissue normal in TCGA) from the TCGA-NSCLC project^52^, which we refer to as OOD in this paper. Because non-cancerous lung tissues share many biological and morphological characteristics with NSCLC slides, distinguishing them poses a particularly challenging and clinically relevant task for OOD detection.

In addition to the binary NSCLC subtyping task, we used five additional datasets totalling 14,932 WSIs, which encompass diverse subtyping tasks across different organs and varying number of cancer subtypes to further evaluate TRUECAM’s generalizability and scalability (Supplementary Table 2). These datasets include the following: (1) Brain tumour subtyping from TCGA (referred to as TCGA-BRTS)—1,225 WSIs for a three-class brain tumour subtyping task across 617 patients, (2) BRACS—547 WSIs for a three-class breast cancer subtyping task across 189 patients^53^, (3) TCGA-BRCA—1,049 WSIs for breast cancer subtyping across 984 patients^54^, (4) TCGA-RCC—925 WSIs for three-class kidney cancer subtyping across 895 patients^38^ and (5) TCGA-OT—11,186 WSIs from 9,149 patients for a 46-class pan-cancer slide-level classification task defined based on the OncoTree classification system^31^.

#### Image preprocessing.

Regardless of distinct model input requirements, all WSIs were segmented and split into appropriate tiles using a standard preprocessing pipeline implemented in Slideflow (version 2.1.0)^55^. Directly feeding large-gigapixel WSIs into a neural network is computationally impractical, so image tiles of manageable sizes were extracted to align with the model’s specifications. For example, foundation models like UNI, CONCH and Prov-GigaPath accept image tiles of size 256 × 256 pixels at ×20 magnification; as we follow the Inception-v3 model developed in ref. 16, it requires image tiles of 299 × 299 pixels at ×10 magnification; unlike UNI, CONCH and Prov-GigaPath, the latest foundation model TITAN accepts image tiles with a size of 512 × 512 pixels at ×20 magnification. The preprocessing pipeline also includes several standard steps to create deep learning-ready datasets. Otsu’s thresholding^56^ was applied to differentiate the foreground (tissue) from the background (empty slide), followed by greyspace filtering to remove background tiles. All extracted tiles were then stain-normalized and saved in TFRecord format for model training, inference and performance evaluation. No pathologist-annotated regions of interest were used; instead, we relied solely on slide-level diagnoses and assigned a uniform weak label to all tiles extracted from a given WSI when needed for model training. Regarding the five additional datasets, we adopted the same data preprocessing approach as previous studies, such as tissue segmentation and tiling^28,29,31^.

#### Dataset configuration.

In the set-up for the specialized model based on Inception-v3, we randomly partitioned the TCGA dataset, allocating 65% of the patients’ data for training, 15% for validation and 20% for calibration and testing. We repeated the random partitioning procedure 20 times, producing 20 independently trained models for evaluation. Within the 20% allocated for calibration and testing, data from 100 patients were used for calibration to establish CP (referred to as the TCGA calibration dataset), while data from 89 patients was set aside for testing (referred to as the TCGA testing dataset). To assess the variability of CP performance, the 20% split was randomized 500 times, generating distinct calibration and testing datasets for each model. As a result, we report model performance based on 20 models × 500 evaluations per model. The CPTAC dataset was fully used for external validation to assess the effectiveness of TRUECAM in scenarios that need transferability, with data from 100 patients designated for calibration to establish CP (referred to as the external CPTAC calibration dataset) and the remaining 316 patients reserved for testing (referred to as the external CPTAC testing dataset). Similarly, the CPTAC dataset was randomly partitioned 500 times to evaluate the variability of CP performance.

In the scenario of using foundation models for NSCLC subtyping, which involves training a separate model using latent tile representations generated by foundation models, the TCGA and CPTAC datasets were used independently for the entire model training and evaluation process. Each dataset was partitioned into three subsets: 65% for training, 15% for validation and 20% for calibration and testing. Consistent with the strategy used for Inception-v3 evaluation, this partitioning and model training process was repeated 20 times, with each involving 500 random splits to establish and evaluate CP.

For all non-lung cancer subtyping datasets except TCGA-OT, we applied the same procedure as for TCGA (described above), partitioning each dataset into training (65%), validation (15%) and testing (20%). This random split was repeated five times, resulting in five independently trained models for evaluation. We report the mean with 95% confidence interval (95% CI) of performance metrics across these five folds. For TCGA-OT, we followed the data split configuration from ref. 31 and used non-parametric bootstrapping with 1,000 samples to estimate the mean and standard deviation of model performance. For the additional NSCLC subtyping dataset QMH-NSCLC, we used the same 65%/15%/20% split for training, validation and calibration/testing but performed the partitioning only once and applied non-parametric bootstrapping with 150 samples to estimate the mean and standard deviation of model performance.

### SNGP

We leveraged SNGP ^57,58^ to provide a principled estimation of data uncertainty in deep learning models for NSCLC subtyping. SNGP introduces two important modifications to a regular neural network: (1) incorporating spectral normalization into the hidden layers and (2) replacing the conventional dense output layer with a Gaussian process. Incorporating spectral normalization ensures that the transformation from the input x in the original input space to the latent representation in the penultimate layer h(x) preserves the relative distances. In other words, the distance $\|h\left(x_{1}\right)-h\left(x_{2}\right){\|}_{\text{H}}$ between representations of any two image data points $\left(x_{1},x_{2}\right)$ in the latent space meaningfully reflects their distance $\|x_{1}-x_{2}{\|}_{\text{X}}$ in the original data space, relative to other data pairs. In practice, adding spectral normalization addresses a problem in training neural networks known as feature collapse^59^. This issue occurs when OOD data or data from different classes, which are geometrically distant from remaining data in the input space, are unexpectedly mapped to nearby points in the latent space, thus leading to unreasonable uncertainty estimations. Mathematically, the spectral normalization operation is equivalent to requiring the mapping function h(⋅) to satisfy the following bi-Lipschitz condition for any pair of inputs $x_{1}$ and $x_{2}$^58,60^:

(1) $$L_{1}\times \|x_{1}-x_{2}{\|}_{\text{X}}\leq \|h\left(x_{1}\right)-h\left(x_{2}\right){\|}_{\text{H}}\leq L_{2}\times \|x_{1}-x_{2}{\|}_{\text{X}},$$

where $L_{1}$ and $L_{2}$ are the positive lower and upper Lipschitz bounds $(0\;<L_{1}<1<L_{2}\text{)}$ of feature extractor h(⋅), and $\|\cdot {\|}_{\text{X}}$ and $\|\cdot {\|}_{\text{H}}$ correspond to the distance metrics chosen for the original and latent space, respectively. The lower Lipschitz bound $L_{1}$ serves to preserve distances in the latent space and the upper Lipschitz bound $L_{2}$ helps enforce the smoothness and robustness of the neural network by limiting over-sensitive transformations to perturbations in the input space of x.

In SNGP, the bi-Lipschitz constraint is enforced by applying spectral normalization to neural network weights ${\left\{W_{l}\right\}}_{l=1}^{l-1}$ (ref. 61), where l represents the l th hidden layer of the neural network. During each training step, the spectral normalization method first estimates the spectral norm $\hat{\lambda }\approx \|W_{l}{\|}_{2}$ using the power iteration method^62^ and then normalizes the neural network weights as follows:

(2) $${\hat{W}}_{l}=\left\{\begin{array}{l} cW_{l}/\hat{\lambda },\;\text{if}\;c<\hat{\lambda }\\ W_{l},\;\text{otherwise}\; \end{array},\right.$$

where c is a hyperparameter used to adjust the exact spectral norm upper bound on ${\left\|W_{l}\right\|}_{2}$ (that is, ${\left\|W_{l}\right\|}_{2}\leq c$).

SNGP then estimates data uncertainty by leveraging the preserved distances in the latent space, achieved by replacing the dense output layer with a Gaussian process. Consider a dataset consisting of N training points $𝒟={\left\{x_{i},y_{i}\right\}}_{i=1}^{N}$, and let $h_{i}\left(x_{i}\right)$ denote the representation of $x_{i}$ in the second-to-last layer of neural network. The output $f_{N\times 1}={\left[f\left(h_{1}\right),f\left(h_{2}\right),\cdots ,f\left(h_{N}\right)\right]}^{T}$ of Gaussian process follows a multivariate Gaussian distribution:

(3) $$f_{N\times 1}∼N\left(0_{N\times 1},K_{N\times N}\right),\;\text{where}\;K_{i,j}=\exp\left(\frac{-{\left\|h_{i}\;-\;h_{j}\right\|}_{2}^{2}}{2}\right).$$

Conditional on the learned latent representation $h_{i}$, SNGP approximates Gaussian process using the Laplace approximation with the random Fourier feature (RFF) expansion^63^. Specifically, SNGP approximates the Gaussian process prior in equation (3) with a low-rank approximation to the kernel matrix $K={\text{Φ}\Phi }^{T}$ using random features^64^:

(4) $$f_{N\times 1}∼N\left(0_{N\times 1},{\text{ΦΦ}}_{N\times N}^{T}\right){,\;\text{where}\;\text{Φ}}_{i}=\sqrt{\frac{2}{D_{L}}}\cos\left(-W_{L}h_{i}+b_{L}\right)\text{,}\;$$

where ${\text{Φ}}_{i}$, is the last layer of the neural network with a dimension $D_{L}$, $W_{L}$ is a fixed weight matrix randomly generated from 𝒩(0,1) and $b_{L}$ is a fixed bias vector randomly generated from a uniform distribution u(0,2π). As $W_{L}$ and $b_{L}$ are fixed, the RFF approximation to the Gaussian process prior for the *k*th logit of the classification problem (in equation (3)) can be reformulated as follows:

(5) $$g_{k}\left(h_{i}\right)=\sqrt{\frac{2}{D_{L}}}\cos{\left(-W_{L}h_{i}+b_{L}\right)}^{T}\times \beta_{k},k=1,\cdots ,K,$$

where $\beta_{k}∼𝒩(0,I)$, K denotes the number of classes in the classification problem, and $\beta ={\left\{\beta_{k}\right\}}_{k=1}^{K}$ are the only learnable parameters in the output layer.

Overall, the trainable parameters consist of β along with the weights and biases $\alpha ={\left\{W_{l},b_{l}\right\}}_{l=1}^{l-1}$ in the first L−1 layers of the neural network. The RFF approximation to Gaussian process reduces the high-dimensional Gaussian process to a standard Bayesian linear model while also producing a closed-form posterior that is end-to-end trainable alongside the rest of the neural network using stochastic gradient descent. After inferring the MAP estimate of $\hat{\beta_{k}}$, for an unobserved point $x^{*}$, the first L−1 layers of SNGP serve as a feature extractor to derive its latent representation ${\text{Φ}}^{*}=\sqrt{\frac{2}{D_{L}}}\cos\left(-W_{L}h\left(x^{*}\right)+b_{L}\right)$ Next, the mean and variance associated with the *k*th logit in the classification problem are computed as follows:

(6) $$\mu_{k}\left(x^{*}\right)={\left[{\text{Φ}}^{*}\right]}^{T}\hat{\beta_{k}},\sigma_{k}\left(x^{*}\right)={\left[{\text{Φ}}^{*}\right]}^{T}{\text{Σ}}_{k}{\text{Φ}}^{*}.$$

Finally, the predictive distribution for $x^{*}$ is calculated as follows:

(7) $$p\left(y^{*}|x^{*}\right)=\int_{s∼𝒩\left(\mu_{k}\left(x^{*}\right),\sigma_{k}\left(x^{*}\right)\right)}\gamma (s),$$

where γ denotes the softmax activation function.

### CP

CP is a distribution-free approach for constructing prediction sets for arbitrary prediction or classification algorithm. It provides statistical guarantees of a bounded error rate for model outputs, rigorously aligned with the error level specified by developers or service providers under data exchangeability assumption^65^. Consider a classification problem comprising K classes. Let $𝒟={\left\{x_{i},y_{i}\right\}}_{i=1}^{T}$ be independently and identically distributed (i.i.d.) data points sampled from distribution 𝒳×𝒴, where $x_{i}∼𝒳,y_{i}∼𝒴$, $y_{i}\in \{1,\cdots ,K\}$, and α∈[0,1] denotes a specified error level (also referred to as significance level). Then the objective of CP is to construct a prediction set for each data point, denoted as

(8) Γ:𝒳→{subsetof{1,2,⋯,K}},

such that for a new i.i.d. data point $\left(x^{*},y^{*}\right)∼P(𝒳,𝒴)$, the probability that the prediction set fails to contain the truth label is α bounded. This is formally expressed as follows^66^:

(9) $$\left.1-\alpha \leq \mathbb{P}\left(y^{*}\in \text{Γ}\left(x^{*}\right)|x^{*}\right)\right)\leq 1-\alpha +\frac{1}{T\;+\;1},$$

where the probability is taken over the data ${\left(x_{i},y_{i}\right)}_{i=1}^{T}\cup \left(x^{*},y^{*}\right)$.

By design, CP guarantees that the prediction set $\Gamma (x^{*})$ contains the true label $y^{*}$ with a probability of at least 1−α. The bounded error rate is achieved by generating a set of likely class labels for the inputs with insufficient certainty in a systematic way, in contrast to the single-value (in regression) or single-label (in classification) outputs produced by regular machine learning models.

Using two separate and disjoint datasets for model training and calibration, the development of an inductive conformal predictor consists of three key steps:

1. Define a nonconformity score. Conformity reflects how similar or conformal a new data point is to the training data^67^ and is typically quantified using a nonconformity score. In classification tasks, the nonconformity score is oftentimes defined as $s\left(x_{i},y_{i}\right)=1-\hat{f}{\left(x_{i}\right)}_{y_{i}}$, where $\hat{f}{\left(x_{i}\right)}_{y_{i}}$ denotes the probability assigned by the trained model $\hat{f}$ to the true class $y_{i}$, for the input $x_{i}$. Thus, the lower the model’s confidence in correctly classifying $x_{i}$ as $y_{i}$, the higher the corresponding nonconformity score. We used this nonconformity score throughout the study.
2. Determine the nonconformity threshold. We then computed the nonconformity score $s\left(x_{i},y_{i}\right)$ for each data point $x_{i}(i=T+1\text{,}\;\cdots ,T+R)$ in the calibration set and obtain a set of nonconformity scores: $\left\{s\left(x_{T\;+\;1},y_{T\;+\;1}\right),s\left(x_{T\;+\;2},y_{T\;+\;2}\right),\cdots ,s\left(x_{T\;+\;R},y_{T\;+\;R}\right)\right\}$. We ranked these scores in ascending order and determine the threshold $\hat{q}$ as the $\frac{\left[(R+1)(1-\alpha )\right]}{R}$ quantile of these ordered scores. This threshold ensures that the resulting prediction sets have a coverage rate of at least 1−α on new i.i.d. datasets.
3. Generate prediction sets. For a new data point $x^{*}$, the prediction set is formed by including all class labels k∈{1,2,…,K} whose corresponding scores $s\left(x^{*},k\right)$ are smaller than or equal to $\hat{q}$. Mathematically, we have the following:

(10) $$\text{Γ}\left(x^{*}\right)=\left\{k:s\left(x^{*},k\right)\leq \hat{q}\right\},k=1,\;\ldots \;,K.$$

Unlike regular UQ methods, CP converts the traditional heuristic concept of uncertainty, often represented by softmax outputs, into a statistically meaningful measure in the form of a prediction set. By following the steps described above, CP ensures to generate statistically valid prediction sets that contain the true label with a pre-specified coverage of 1−α. This approach offers a standardized framework for constructing reliable confidence intervals that enables machine learning systems to deliver robust outputs while upholding responsible and trustworthy scientific inferences.

### Deep learning models

To assess the effectiveness of TRUECAM, we evaluated two types of AI architecture: task-specific models (referred to as specialized models) and models based on pathology foundation models (referred to as foundation models). We trained Inception-v3 from scratch, a widely used convolutional neural network architecture for image inference^26,27^, to serve as a representative classifier for specialized medical imaging tasks. All Inception-v3 models were trained at the tile level, with slide-level classifications obtained by averaging tile-level outputs across each WSI^16^. For patients with multiple WSIs, the final patient-level diagnosis was derived by averaging the slide-level classifications across all WSIs for that patient. In this context, we used TCGA for model training and internal validation, while we used CPTAC as an independent external validation dataset to assess TRUECAM’s effectiveness in scenarios requiring model transferability. We further investigated four digital pathology foundation models that exemplify the recent advancements in large, general-purpose models in the field: (1) UNI^28^, (2) CONCH^29^, (3) Prov-GigaPath^30^ and (4) TITAN^31^. We paired these models with multiple cutting-edge MIL mechanisms (that is, ABMIL^68^, CLAM^38^ and TransMIL^69^) where applicable. As foundation models are pretrained on diverse datasets, offering strong knowledge transferability as an image encoder, and downstream task-specific classifiers are typically lightweight and easy to train, our evaluation focused solely on the setting where each healthcare organization trains and evaluates its own site-specific model using its data. All considered datasets were used to mimic this setting such that each dataset represents a distinct healthcare organization and independently supports the required model training and validation. Our evaluation primarily focused on UNI and CONCH, with additional results provided for Prov-GigaPath and TITAN to showcase the generalizability of TRUECAM’s advantages.

### Specialized models based on Inception-v3.

We trained three deep learning models based on the Inception-v3 architecture^26,27^. The first is the original deterministic version, which does not include UQ. The other two models do incorporate UQ techniques: (1) MC Dropout^11^ and (2) SNGP. Inception-v3 is a 48-layer deep convolutional neural network designed to capture spatial features at varying scales by using parallel convolution operations with different filter sizes within the same layer. To address the vanishing gradient problem and provide additional supervision signals, Inception-v3 integrates an auxiliary classifier to propagate label information to earlier network layers. In addition, Inception-v3 uses bottleneck layers to reduce dimensionality and computational costs. These design features collectively enhance performance and resource efficiency, rendering Inception-v3 widely adopted for a diverse range of general computer vision and medical imaging applications. Supplementary Table 3 provides parameter and training details.

MC Dropout was used as the primary baseline approach for UQ^11^. By assuming that the prior of neural network weights follows a standard normal distribution, MC Dropout is mathematically equivalent to sampling from a variational posterior consisting of two independent Gaussians with fixed covariance. To implement MC Dropout in Inception-v3, we followed the design adopted by ref. 16. Specifically, we appended two fully connected layers, each containing 1,024 neurons, to the end of the neural network and applied dropout with a rate of 0.1 after each fully connected layer. During inference, each image tile was forward-passed through the dropout-enabled network five times. The standard deviation of the model’s predictions across these five passes was calculated as the measure of tile-level uncertainty. The tile-level predictions and their corresponding uncertainty were then aggregated to produce slide- and patient-level predictions, along with the associated uncertainty estimates.

In addition, we included VBLL as another baseline for UQ. We adopted a post-training approach to optimize a variational last layer atop features extracted from a pretrained neural network. Both the discriminative and generative classifiers were implemented as a two-layer dense neural network with a hidden dimension of 128 and diagonal parameterization. All other hyperparameters for VBLL training followed the configuration described in ref. 32. Performance results for VBLL, MC Dropout and SNGP in the NSCLC subtyping task are summarized in Supplementary Table 8.

We trained specialized models from scratch using a 24 GB NVIDIA RTX 4090 GPU with a batch size of 64, where image tiles were resized to 299 × 299 pixels. We adopted a learning rate scheduler with an initial learning rate of 0.0003 and a decay rate of 0.98 every 512 steps. Each model was trained for four epochs using the Adam optimizer with the first and second moment exponential decay rate setting to 0.9 and 0.999, respectively. During model training, we monitored model performance on the validation dataset and saved the best-performing model for evaluation on the testing data. Standard data augmentation techniques, including random horizontal and vertical flips, 90° rotations, JPEG compression with a 50% probability (quality range, 50–100) and Gaussian blur with a 10% probability (sigma, 0.5–2.0), were used to augment the training process of each model.

#### Foundation models.

TRUECAM seamlessly integrates with pathology foundation models for trustworthy cancer subtyping. In this study, we explored integrating TRUECAM with two types of foundation model: tile-level (or patch-level) models, including UNI^28^ and CONCH^29^, and slide-level models, including Prov-GigaPath^30^ and TITAN^31^, both using the vision transformer-based architecture as image encoders. All of them are general-purpose models systematically pretrained on diverse sources of histopathology images using self-supervised learning paradigms. Among them, CONCH, Prov-GigaPath and TITAN are visual-language models designed to align image and text modalities within their representation space, whereas UNI is exclusively image-pretrained. These foundation models show exceptional adaptability and knowledge transferability through image encoding, making them highly effective for a wide range of downstream tasks in histopathology, including image classification, region-of-interest retrieval and cell type segmentation.

To adapt tile-level foundation models for cancer subtyping, we used the image encoders of UNI and CONCH to extract tile embedding (that is, latent representations), concatenated the resulting representations for each slide and applied the widely used MIL-based mechanisms, including ABMIL, CLAM^38^ and TransMIL^69^, for weakly supervised classification using slide-level diagnostic labels. The ABMIL model architecture and training configuration followed those established in UNI^28^ and CONCH^29^. Specifically, ABMIL includes a fully connected layer with a rectified linear unit activation function to transform tile-level features into 512-dimensional embedding, followed by an intermediate layer with size 384. Finally, another fully connected layer maps the attention-pooled slide-level representations to logits and class probabilities through softmax normalization. Dropout was applied at multiple stages: a rate of 0.1 for the input features and 0.25 after each intermediate layer. In addition, we used the CLAM-SB (single-attention branch) and TransMIL models, with parameter configurations following ref. 38 and ref. 69, respectively. To incorporate SNGP, we added spectral normalization to all fully connected layers and replaced the final layer with a RFF-approximated Gaussian process. All MIL-based models were trained via a maximum of 20 epochs using the AdamW optimizer^70^ with a cosine learning rate scheduler, an initial learning rate of 1 × 10^−4^ and cross-entropy loss.

For slide-level foundation models, we fine-tuned the slide encoder of Prov-GigaPath and performed linear probing on the slide representations of TITAN for downstream classification tasks as suggested by refs. 30,31. For both models, we appended a Gaussian process layer to the slide-level representations without applying spectral normalization, as the representations extracted from Prov-GigaPath and TITAN inherently preserve distances effectively, due to their dedicated self-supervised training strategy.

### EAT

Regardless of the number of subtypes *5* in a given subtyping task, ambiguous tiles in a WSI were identified and eliminated using an entropy-based metric. While the underlying concept of entropy is shared, specialized models (for example, Inception-v3) and foundation models (for example, UNI, CONCH, Prov-GigaPath and TITAN) differ slightly in their strategies to leverage it.

For the setting of specialized models (for example, Inception-v3), we performed *k*-means clustering for each tested k(k>S) using the latent representations of all training-set tiles obtained from SNGP. For each resultant cluster i(i={1,2,⋯,k}), we define the cluster label assignment entropy as $ℍ(k,i)=-\sum_{j=1}^{S}p(k,i,j)\log p(k,i,j)$, where p(k,i,j) denotes the fraction of tiles labelled as subtype j in cluster i. This measure quantifies the degree of subtype heterogeneity within a cluster, with higher values indicating greater ambiguity. The set of clusters were then ranked in descending order based on ℍ(k,i), and the top m clusters were designated as ambiguous clusters. A new specialized model was then trained on tiles that are not from these ambiguous clusters. In the inference stage, tiles from a new WSI that were assigned to ambiguous clusters were eliminated and the remaining tiles were processed by the newly trained model for inference (Fig. 1c). The values for k and m were determined based on the validation performance of the retrained model.

In the foundation model setting, estimating data ambiguity for EAT requires a tile-level classifier aligned with the target subtyping task. As the tile-level features extracted from foundation model encoders are not compatible with the input of the specialized model, we used AutoGluon (version 1.1.1)^71^ to train a lightweight proxy classifier from scratch. AutoGluon treats the extracted tile-level representations as tabular data and searches the design space, which is composed of multiple state-of-the-art models (for example, LightGBM, NeuralNet, XGBoost and ExtraTrees) for cancer subtyping. It then automatically ensembles the weights of these base models to maximize validation performance. The tile label assignment entropy of a tile x is estimated as $ℍ(x)=-\sum_{j=1}^{S}p(j,x)\log p(j,x)$, where p(j,x) denotes the probability of assigning x to subtype j by the proxy classifier. Those tiles with their label assignment entropy greater than a threshold were excluded from inference. This threshold was determined based on the validation performance of the MIL model that was used.

### UQ

To derive data uncertainty in the context of SNGP, once the model is properly trained, the RFFs for $x^{*}$ can be calculated as ${\text{Φ}}^{*}=\sqrt{\frac{2}{D_{L}}}\cos\left(-W_{L}h_{i}\left(x^{*}\right)+b_{L}\right)$. With this $D_{L}$-dimensional latent representation of $x^{*}$, the tile-level uncertainty estimates can be derived as $\sqrt{\tau {\left[\Phi^{*}\right]}^{T}{\left(\Phi_{N\times D_{L}}^{T}\Phi_{N\times D_{L}}+\tau I\right)}^{-1}\Phi^{*}}$, where τ denotes the ridge factor. SNGP requires only a single forward pass of an image tile through the neural network to extract the corresponding RFF features for uncertainty estimation. By contrast, MC Dropout requires multiple forward passes of the same image tile to quantify prediction uncertainty. This makes SNGP more computationally efficient during inference, which is particularly important for large-scale, safety-critical applications with stringent real-time inference requirements like cancer diagnostics.

It is important to note that SNGP and CP differ in their approaches for UQ. Beyond the regular classification probability of $x^{*}$ produced by a deep learning model, SNGP generates an estimated standard deviation $\sigma \left(x^{*}\right)$ as the uncertainty measure for $x^{*}$. By contrast, CP does not produce any explicit uncertainty measure. Instead, it operates within the class probability space established by the deep learning model and constructs a prediction set $\Gamma \left(x^{*}\right)$ for $x^{*}$ by including labels with the corresponding prediction probabilities beyond a threshold. Thus, SNGP and CP fundamentally differ in how they quantify uncertainty.

For OOD detection and DSC, two scores were considered: (1) a probability-based OOD score and (2) an uncertainty-based OOD score. The probability-based OOD score aggregates tile-level class probabilities and measures the degree of OOD as $1-\frac{1}{N}\sum_{i=1}^{N}\max_{k}\;p\left({\hat{y}}_{k}|x_{i}\right)$, where $p\left({\hat{y}}_{k}|x_{i}\right)$ denotes the probability of tile i being classified as ${\hat{y}}_{k}$. Unlike the probability-based OOD score, the uncertainty-based OOD score quantifies tile-level prediction uncertainty using the standard deviation estimated by MC Dropout or SNGP. It then uses the first δ tiles with the lowest uncertainty for each patient to represent the patient-level prediction uncertainty. Clearly, the uncertainty-based OOD score offers additional insight beyond what deterministic models can provide, effectively highlighting the inherent decision limitations of deep learning models, as shown by its superior performance in identifying OOD images in the foundation model-based approach.

### CRC

CRC extends regular CP to accommodate a broader range of loss functions that assess different aspects of prediction quality beyond miscoverage, such as accuracy, calibration and other task-specific performance metrics^72^. More concretely, we define the prediction set for x by model $\hat{f}$ as ${\text{Γ}}_{\rho }(x)=\{k:\hat{f}{\left(x\right)}_{k}\geq 1-\rho \}$, where k∈{1,…,K} denotes all possible class labels and ρ is a threshold that controls the conservativeness of the prediction set. Then, for any bounded loss function l(⋅) that is non-increasing as the prediction set ${\text{Γ}}_{\rho }(x)$ gets larger, CRC provides a statistical guarantee on the loss in the following form:

(11) $$E\left[l\left({\text{Γ}}_{\rho }(x),y\right)\right]\leq \alpha .$$

Compared to regular CP, CRC extends CP to control the expected value of any monotone loss function instead of only coverage. The goal of CRC is to determine the lowest possible $\hat{\rho }$ based on the calibration dataset, such that the risk control defined in equation (11) holds for unseen data. In the deployment of pathology AI where In-D and OOD data coexist, the observed coverage over the calibration data guides the identification of $\hat{\rho }$ via binary search. The threshold $\hat{\rho }$ can be iteratively adjusted until the target coverage 1−α is satisfied in the presence of OOD data. After the optimal $\hat{\rho }$ is identified, CRC then applies it to the unseen data such that their coverage is 1−α guaranteed.

### Model fairness evaluation

For model fairness evaluation, we examined the performance gap between the best- and worst-performing subgroups based on sex and race^73^. Two performance dimensions were assessed: (1) accuracy of model predictions and (2) size of CP prediction sets. Group-level average values of these metrics were calculated to represent the performance of each corresponding subgroup. As the gap value is always non-negative, a smaller gap indicates a fairer model. Sex and race information is available in both the TCGA and CPTAC datasets. TCGA includes 569 male and 372 female patients, while CPTAC comprises 288 male and 127 female patients, with one patient’s sex not reported. For race, categories with fewer than 20 patients are grouped as ‘Others’. Specifically, in TCGA, patients are grouped into Others (*n* = 98), White (*n* = 678) and Not reported (*n* = 165). In CPTAC, patients are grouped into Asian (*n* = 123), Others (*n* = 49) and White (*n* = 244). Demographic information is summarized in Supplementary Table 1.

## Extended Data

**Extended Data Fig. 1 |:**
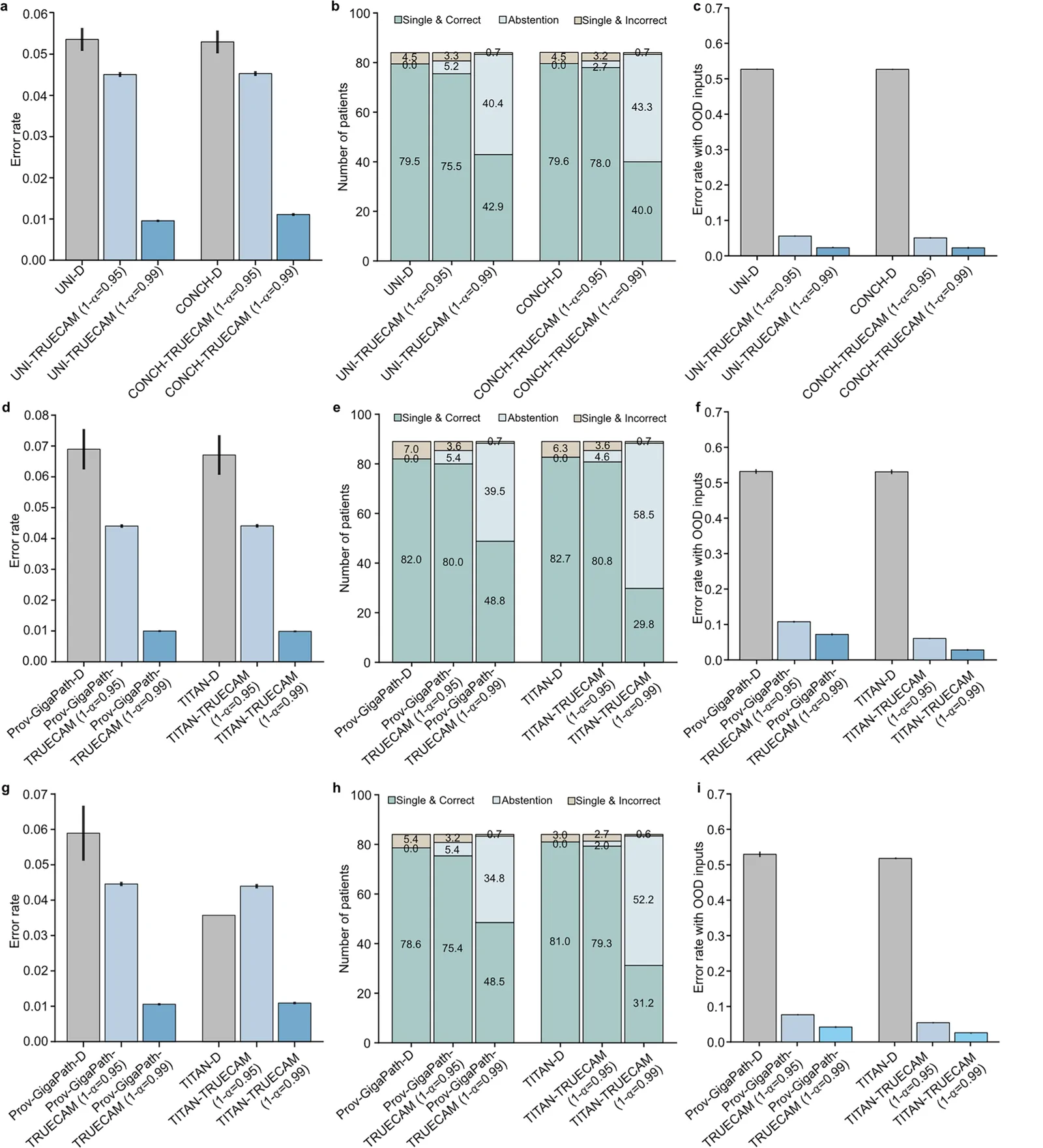
Evaluation of TRUECAM’s performance across four foundation models for NSCLC subtyping: UNI, CONCH, Prov-GigaPath, and TITAN. **a, d, g**, TRUECAM significantly reduced NSCLC subtyping error rates across model types (denoted using suffix “-TRUECAM”) compared to their original deterministic versions (denoted using suffix “-D”), adhering to the pre-specified true label coverage 1−α. The only exception is TITAN-TRUECAM with 1−α=0.95, where TITAN-D achieved an error rate below 0.05. As a result, CP produced a zero-prediction set size (no prediction) to maintain the desired coverage of 0.95. **b, e, h**, Patient-level classification breakdown for models with and without TRUECAM. **c**, **f**, **i**, TRUECAM achieved significantly lower error rates in real-world NSCLC subtyping scenarios involving a 1:1 mix of in-domain and out-of-domain inputs. Evaluations in a-c and g-i are based on the CPTAC testing data, whereas those in d-f are based on the TCGA testing data. For **a**, **c-d, f-g, i**, data are presented as mean ± 95% confidence intervals across n = 20 independently trained models, each with 500 conformal prediction evaluations. All foundation models were paired with ABMIL for cancer subtyping. NSCLC: non-small-cell lung cancer; OOD, out-of-domain; D, Deterministic.

**Extended Data Fig. 2 |:**
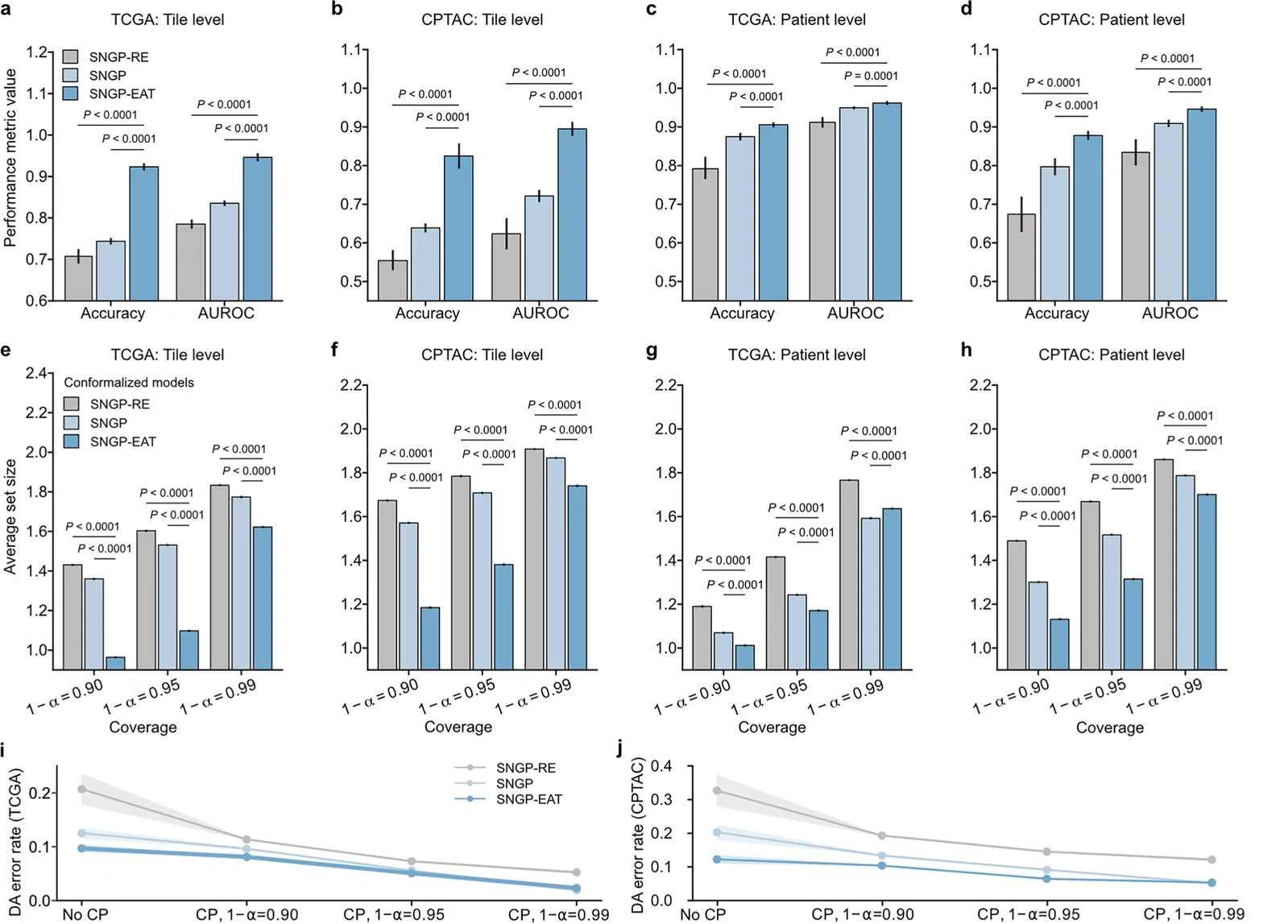
Impact assessment of EAT on classification and CP performance for NSCLC subtyping using Inception-v3 as the backbone. **a**, Tile-level classification performance on TCGA. **b**, Tile-level classification performance on CPTAC. **c**, Patient-level classification performance on TCGA. **d**, Patient-level classification performance on CPTAC. **e**, Tile-level prediction set size on TCGA for three distinct values of significance level α. **f**, Tile-level prediction set size on CPTAC. **g**, Patient-level prediction set size on TCGA. **h**, Patient-level prediction set size on CPTAC. **i**, Patient-level DA error rate on TCGA before and after activating CP. **j**, Patient-level DA error rate on CPTAC before and after activating CP. For **a-h**, data are presented as mean ± 95% confidence intervals across n=20 independently trained models. For **e-h**, each trained model is paired with 500 CP evaluations (See details in the Method), with statistical significance assessed using a one-sided Wilcoxon signed-rank test **a-h**. SNGP, spectral-normalized neural Gaussian process; RE, random elimination; EAT, elimination of ambiguous tiles; DA, definitive-answer.

**Extended Data Fig. 3 |:**
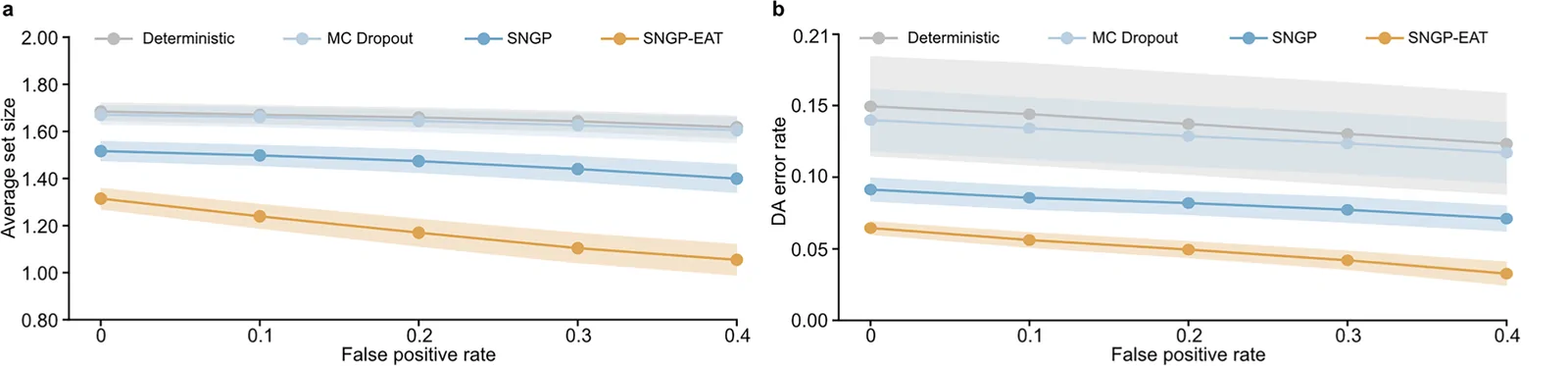
Impact of OOD detection-based distribution shift control on the average set size of CP and the definitive-answer error rate in the CPTAC testing dataset. **a**, Average set size as a function of false positive rate for OOD detection after applying distribution shift control. **b**, Definitive-answer error rate as a function of false positive rate for OOD detection after applying distribution shift control. Mean values and the corresponding 95% confidence intervals are based on n=20 independently trained models, each with 500 CP evaluations (See details in the Method). SNGP, spectral-normalized neural Gaussian process; EAT, elimination of ambiguous tiles; DA, definitive-answer.

**Extended Data Fig. 4 |:**
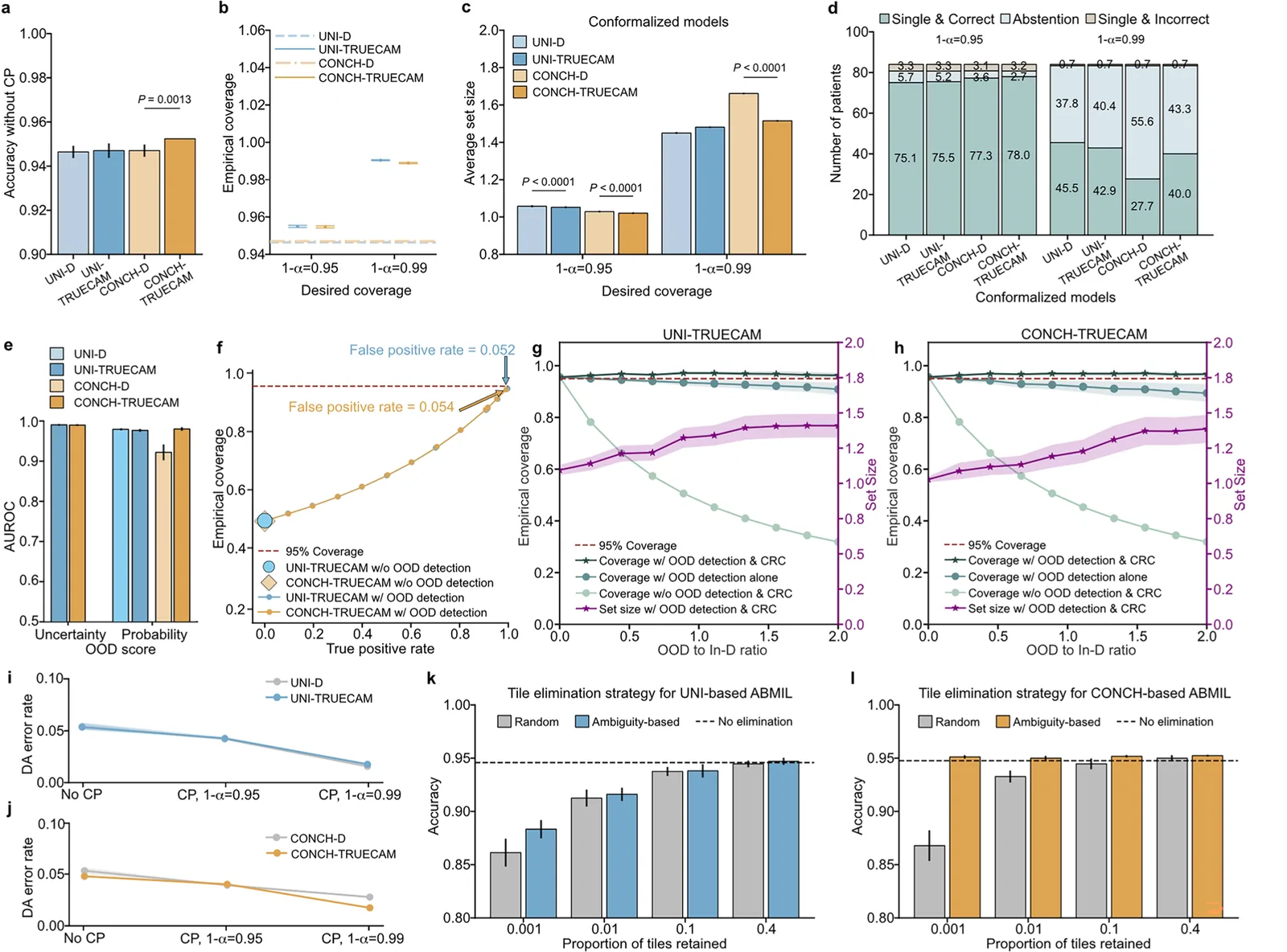
Assessment of TRUECAM using the foundation models UNI and CONCH. Results on CPTAC are reported. **a**, Classification accuracy of foundation model-based ABMIL with (denoted using suffix “-TRUECAM”) and without TRUECAM (that is, the original version of foundation model-based ABMIL, denoted using suffix “-D”), evaluated without CP (n=20 independent runs). **b**, Comparison of empirical coverage with and without TRUECAM (n=20). **c**, Comparison of average set size of CP with and without TRUECAM(n=20). **d**, Comparison of patient classification breakdown with and without TRUECAM. **e**, Performance of two distinct OOD scores in identifying slides outside a model’s scope, which was established using the CPTAC training data. The uncertainty-based score is not applicable to the original versions of UNI and CONCH (n=20). **f**, Empirical coverage with α=0.05 as a function of the true positive rate for OOD detection. **g, h**, Empirical coverage and set size of CP for UNI-TRUECAM and CONCH-TRUECAM under CRC with a false positive rate of 0.052 for UNI and 0.054 for CONCH, shown as a function of OOD-to-In-D ratio. **i,j**, Comparison of patient-level DA error rate with and without TRUECAM in varying contexts of CP. **k, l**, Comparison of classification accuracy for UNI-TRUECAM and CONCH-TRUECAM using TRUECAM’s ambiguity-based tile elimination strategy versus random tile elimination, shown as a function of the proportion of tiles retained per slide. See Supplementary Table 28 for an impact assessment of MIL mechanisms on the performance of UNI and CONCH (n=20). For **a-c, e, k-l**, data are presented as mean ± 95% confidence intervals across n=20 independently trained models, with statistical significance assessed using a one-sided Wilcoxon signed-rank test **a, c**. For **g, l**, error bands are presented as mean ± 95% confidence intervals across n=20 independently trained models. ABMIL, attention-based multiple instance learning; CP, conformal prediction; SNGP, spectral-normalized neural Gaussian process; EAT, elimination of ambiguous tiles; DA, definitive-answer.

**Extended Data Fig. 5 |:**
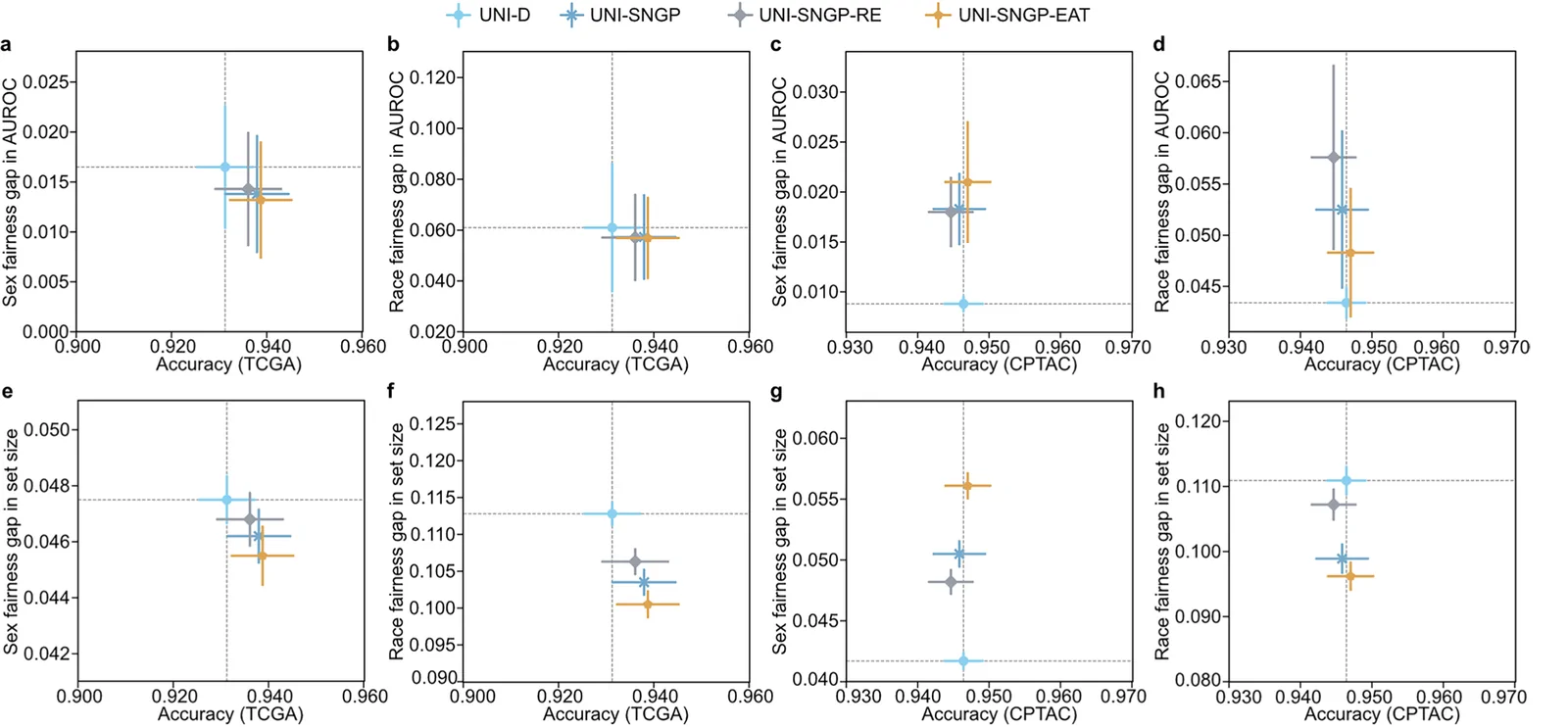
Fairness evaluation of UNI-based ABMIL, measured by the metric value differences between the best- and worst-performing demographic subgroups against the overall accuracy of NSCLC subtyping. **a-d**, Sex- and race-wise AUROC gap versus overall accuracy for discriminating between LUAD and LUSC on TCGA (n=89) and CPTAC (n=84) with CP deactivated. e-h, At α=0.05, sex- and race-wise set size gap versus overall accuracy on TCGA and CPTAC with CP activated. For **a-h**, data are presented as mean ± 95% confidence intervals based on 20 independently trained models, each with 500 CP evaluations (**e-h**). Evaluations on UNI-D and its conformalized version are marked as an overall baseline.

**Extended Data Fig. 6 |:**
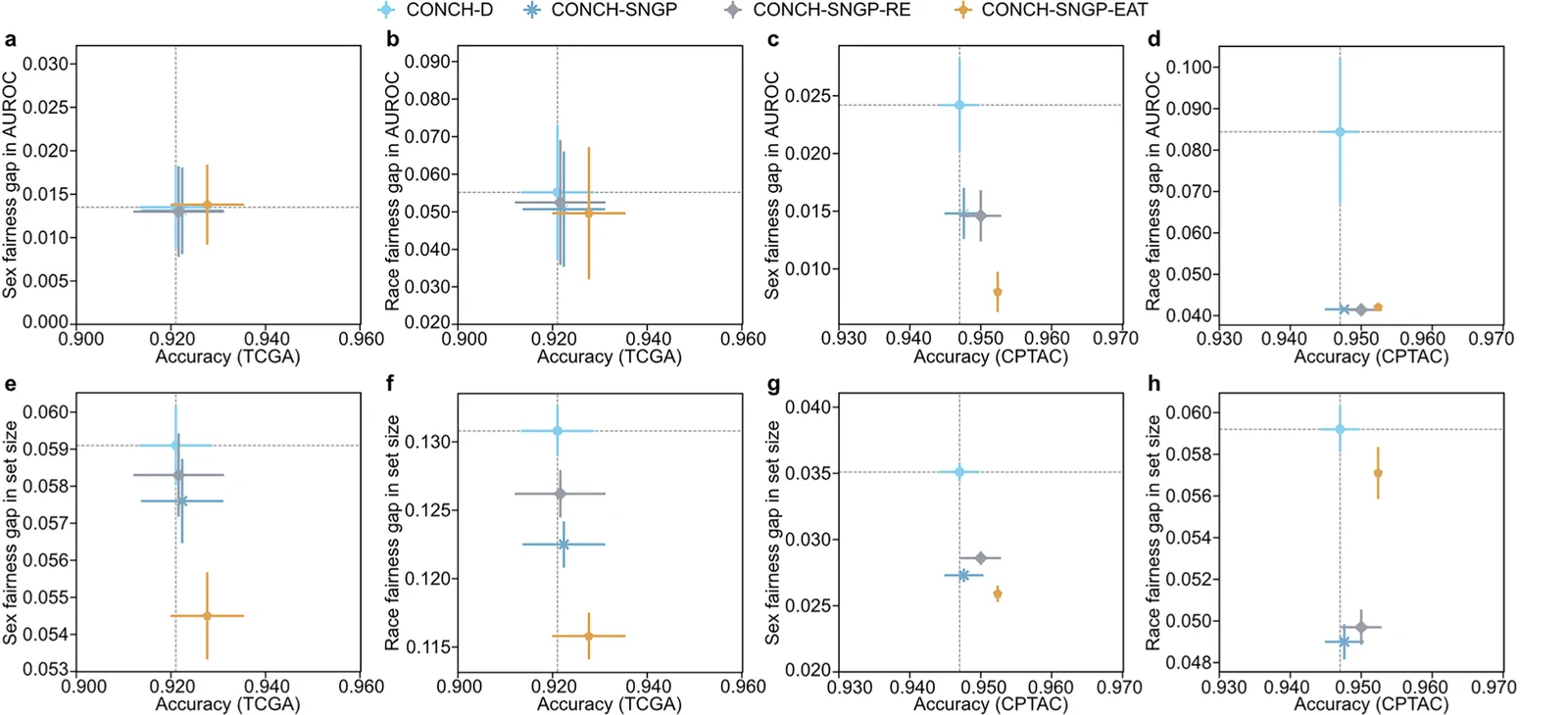
Fairness evaluation of CONCH-based ABMIL, measured by the metric value differences between the best- and worst-performing demographic subgroups against the overall accuracy of NSCLC subtyping. **a-d**, Sex- and race-wise AUROC gap versus overall accuracy for discriminating between LUAD and LUSC on TCGA (n=89) and CPTAC (n=84) with CP deactivated. **e-h**, At α=0.05, sex- and race-wise set size gap versus overall accuracy on TCGA and CPTAC with CP activated. For **a-h**, data are presented as mean ± 95% confidence intervals based on 20 independently trained models, each with 500 CP evaluations (**e-h**). Evaluations on CONCH-D and its conformalized version are marked as an overall baseline.

**Extended Data Fig. 7 |:**
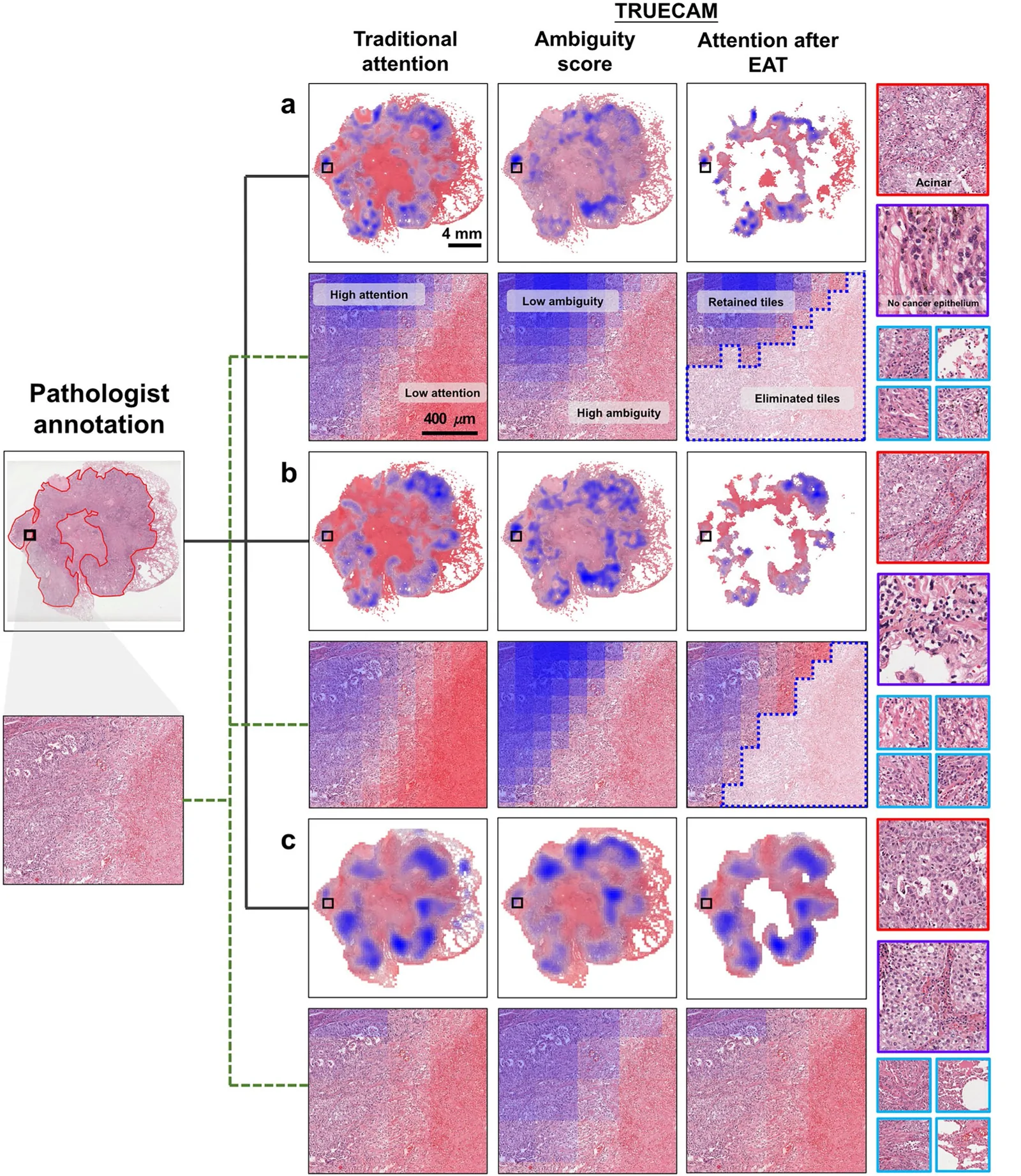
Visualization of a correctly classified LUAD slide randomly selected from the TCGA testing dataset, accompanied by a pathologist’s annotations and model-derived information supporting the inference. The whole-slide image (WSI) and a selected patch (with a zoomed-in view) are visualized in the context of **a**, UNI, **b**, CONCH, and **c**, Inception-v3. The red-highlighted region in the original WSI (first column) indicates the pathologist’s annotation used to guide NSCLC subtyping. Traditional interpretability (second column) was generated from the attention weights of the ABMIL module in foundation models or from the predicted tile-level subtype probabilities in Inception-v3. Tile-level ambiguity (third column) was derived from the UQ module or UQ-informed subtype probabilities in TRUECAM. After applying ambiguity-guided EAT, the attention map (fourth column) was calculated for inference using the SNGP-EAT version of each model with white regions indicating the eliminated tiles. Example tiles (all from the selected patch) visualized in the fifth column include: a randomly selected tile with low ambiguity and a high-attention weight (red border), a tile with low ambiguity and a low-attention weight (purple border), and four randomly selected tiles with high ambiguity (removed by EAT, blue border). The pathologist annotated these tiles as “good indication”, “cannot determine”, and “not relevant”, respectively, demonstrating strong alignment with the TRUECAM-derived interpretability.

**Extended Data Fig. 8 |:**
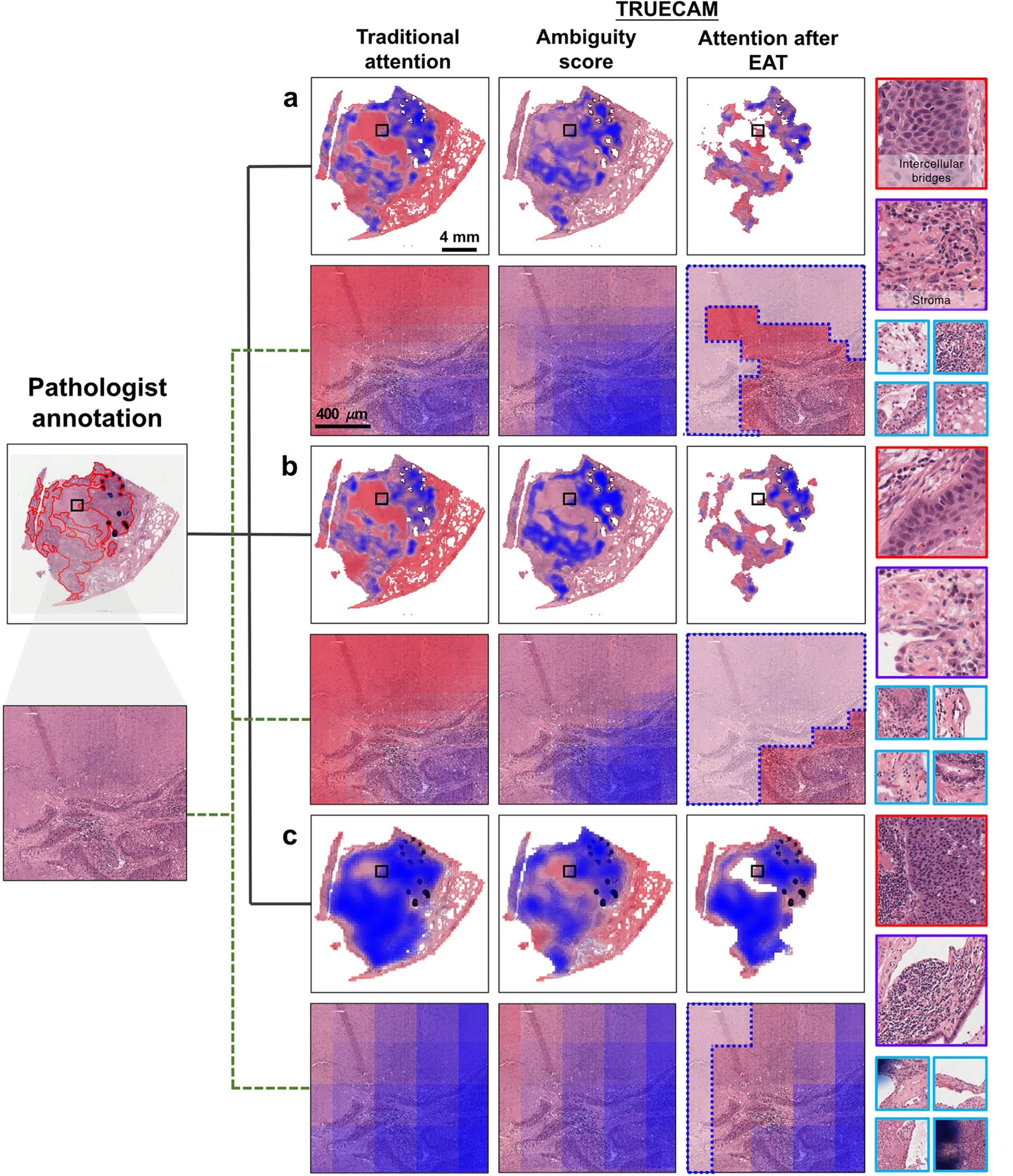
Visualization of a correctly classified LUSC slide randomly selected from the TCGA testing dataset, accompanied by a pathologist’s annotations and model-derived information supporting the inference. The whole-slide image (WSI) and a selected patch (with a zoomed-in view) are visualized in the context of **a**, UNI, **b**, CONCH, and **c**, Inception-v3. The red-highlighted region in the original WSI (first column) indicates the pathologist’s annotation used to guide NSCLC subtyping. Traditional interpretability (second column) was generated from the attention weights of the ABMIL module in foundation models or from the predicted tile-level subtype probabilities in Inception-v3. Tile-level ambiguity (third column) was derived from the UQ module or UQ-informed subtype probabilities in TRUECAM. After applying ambiguity-guided EAT, the attention map (fourth column) was calculated for inference using the SNGP-EAT version of each model with white regions indicating the eliminated tiles. Example tiles (all from the selected patch) visualized in the fifth column include: a randomly selected tile with low ambiguity and a high-attention weight (red border), a tile with low ambiguity and a low-attention weight (purple border), and four randomly selected tiles with high ambiguity (removed by EAT, blue border). The pathologist annotated these tiles as “good indication”, “cannot determine”, and “not relevant”, respectively, demonstrating strong alignment with the TRUECAM-derived interpretability.

**Extended Data Fig. 9 |:**
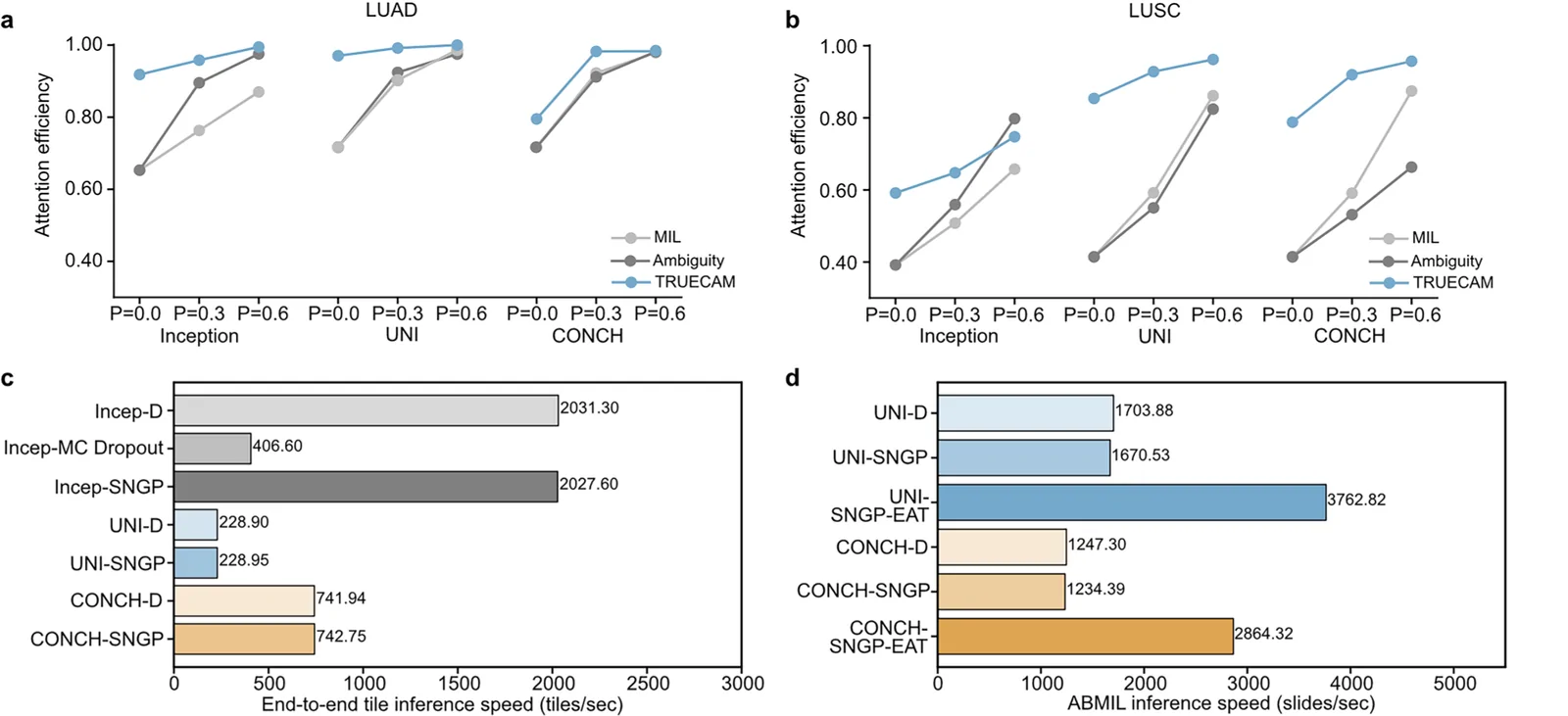
Comparison of attention efficiency and inference speed across different model settings. **a**, Comparison of attention efficiency for traditional attention (that is, MIL), ambiguity, and TRUECAM (that is, attention after EAT) with respect to the LUAD slide shown in Extended Data Fig. 7. P represents the proportion of tiles with the lowest attention scores removed. In the “MIL” setting, attention scores for Inception-v3 and two foundation models correspond to the tile probability and corresponding ABMIL attention scores, respectively, whereas scores in the setting of “Ambiguity” for Inception-v3 and two foundation models correspond to the ambiguity from their own models and AutoGluon models, respectively. **b**, Comparison of attention efficiency for traditional attention (that is, MIL), ambiguity, and TRUECAM (that is, attention after EAT) with respect to the LUAD slide shown in Extended Data Fig. 8. **c**, Comparison of end-to-end tile inference speed. **d**, Comparison of slide-level inference speed for ABMIL-based classification models using pre-extracted tile-level representations from foundation models. Inference speed was measured using an NVIDIA GeForce 4090 (24GB) with a batch size of 512 and 32-bit floating-point precision. Benchmarking excluded the preprocessing and load time with 10 warm-up iterations followed by 30 benchmark iterations. D, Deterministic; MIL, multiple instance learning; ABMIL, attention-based multiple instance learning; SNGP, spectral-normalized neural Gaussian process; EAT, elimination of ambiguous tiles.

## Supplementary Material

supplementary Material

**Supplementary information** The online version contains supplementary material available at https://doi.org/10.1038/s41551-026-01694-8.

## Figures and Tables

**Fig. 1 |:**
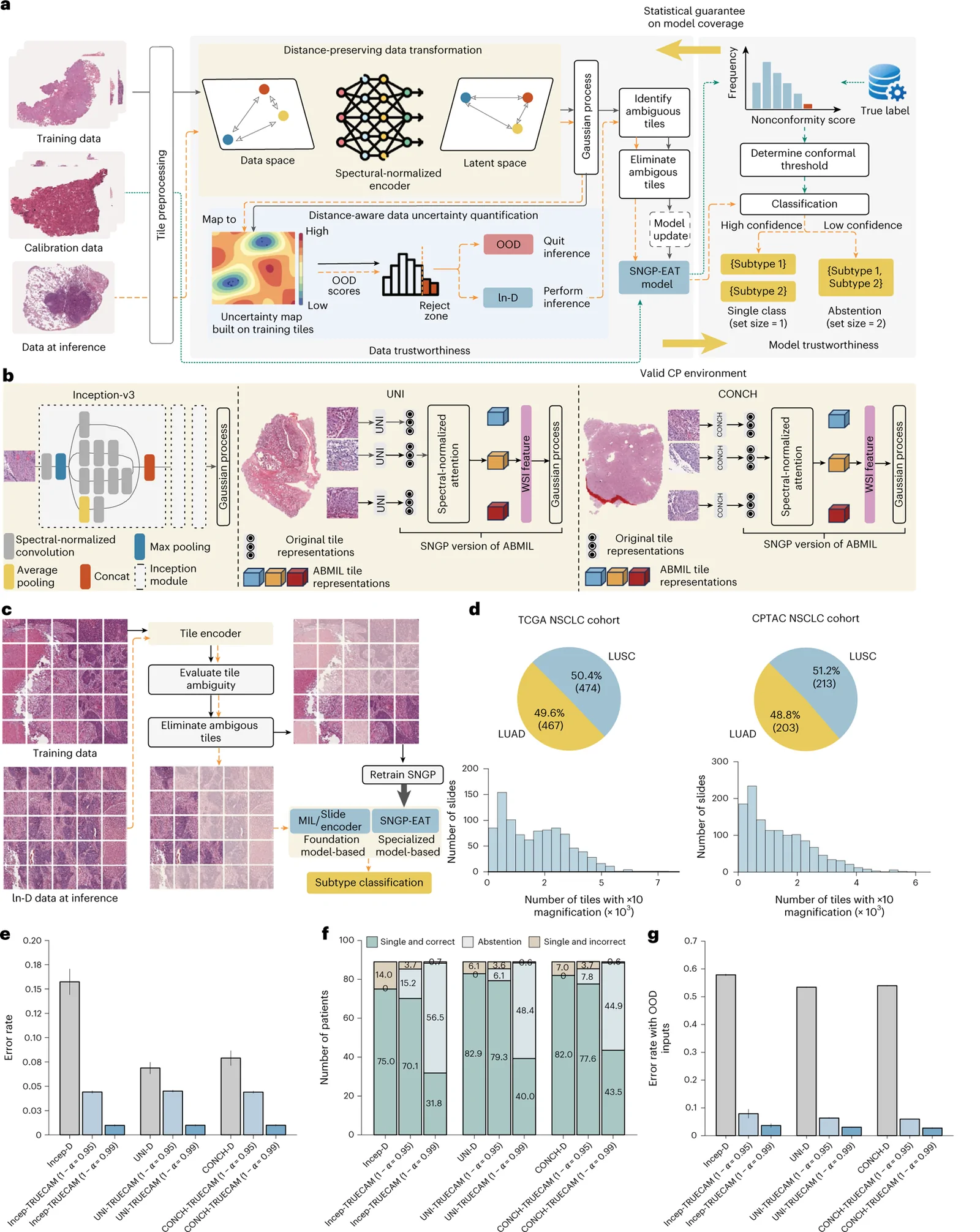
Overview of TRUECAM. TRUECAM is a versatile, model-agnostic digital pathology AI framework, achieving trustworthiness by substantially reducing errors, detecting OOD data and controlling distribution shifts pre-inference, identifying and eliminating ambiguous slide regions and ensuring true label coverage with statistical guarantees via abstention on uncertain inputs. **a**, The architecture of TRUECAM designed to ensure both data and model trustworthiness. **b**, Customization illustration of TRUECAM for deep learning models of varying architecture, complexity and purpose, including Inception-v3, UNI and CONCH. **c**, Illustration of eliminating ambiguous tiles in TRUECAM for slide inference. **d**, Overview of the TCGA and CPTAC NSCLC datasets. **e**, TRUECAM heavily reduced NSCLC subtyping error rates across all model types (denoted using suffix ’-TRUECAM’) compared to their original deterministic versions (denoted using suffix ’-D’), adhering to the pre-specified true label coverage 1−α. **f**, Patient-level NSCLC subtyping breakdown for models with and without TRUECAM. **g**, TRUECAM achieved substantially lower error rates in real-world NSCLC subtyping scenarios involving a 1:1 mix of In-D and OOD inputs. Results shown in **e–g** are based on the TCGA dataset (*n* = 20). UNI and CONCH were paired with ABMIL for inference. See Extended Data Fig. 1 for evaluations based on the CPTAC dataset and two other foundation models (Prov-GigaPath and TITAN). For **e** and **g**, data are presented as mean ± 95% CI based on *n*=20 independently trained models, each with 500 CP evaluations. Incep, Inception-v3.

**Fig. 2 |:**
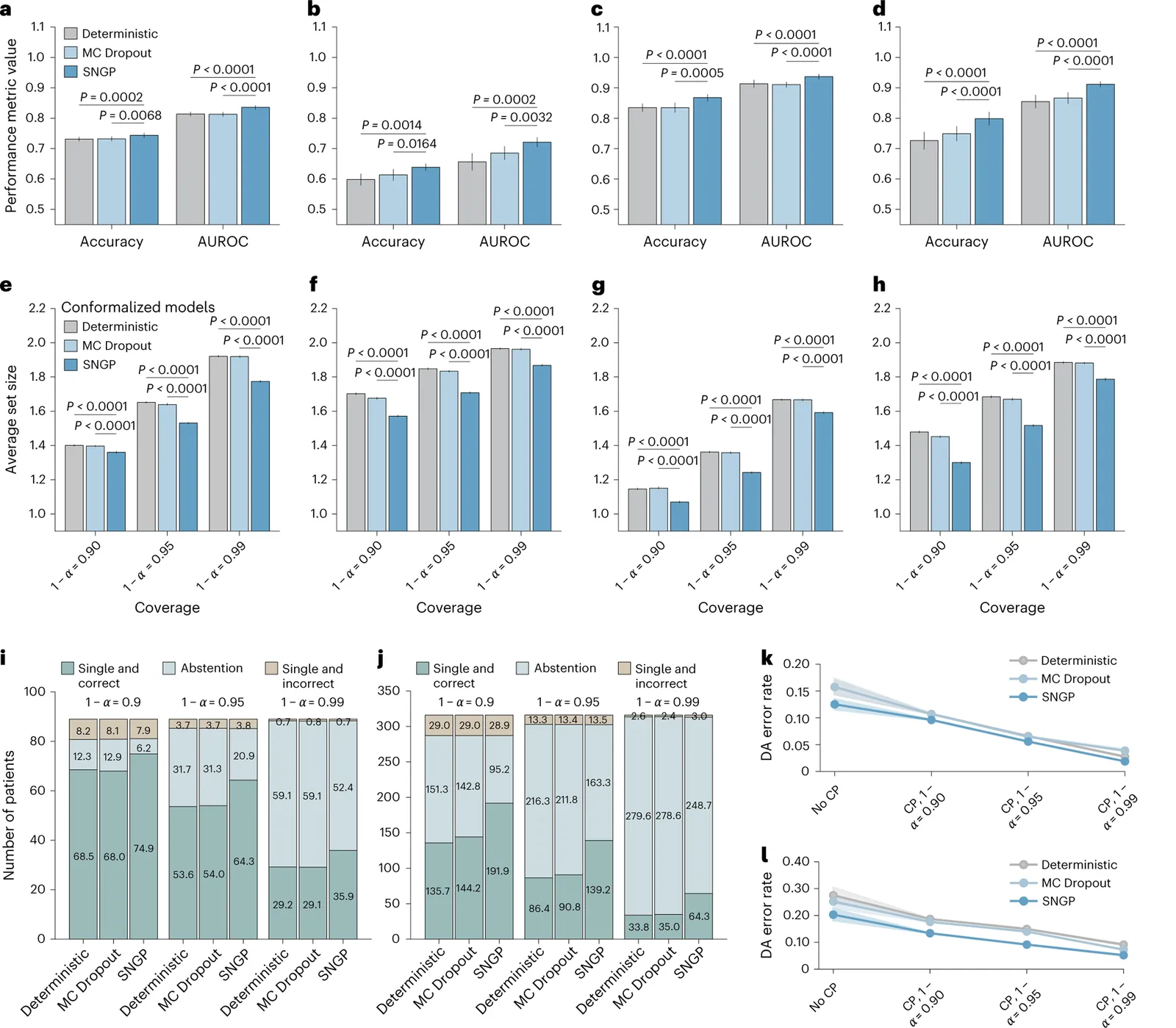
NSCLC subtyping performance of three Inception-v3-based deep neural network models and their conformalized counterparts. **a**, Tile-level performance with respect to the TCGA dataset in terms of classification accuracy and AUROC. **b**, Tile-level performance evaluated on the CPTAC dataset. **c**, Patient-level performance evaluated on the TCGA dataset. **d**, Patient-level performance evaluated on the CPTAC dataset. **e**, Tile-level prediction set size on TCGA for three distinct values of error level α. **f**, Tile-level prediction set size on CPTAC. **g**, Patient-level prediction set size on TCGA. **h**, Patient-level prediction set size on CPTAC. **i**, Patient-level classification breakdown for three conformalized models on the TCGA dataset. **j**, Patient-level classification breakdown on CPTAC. **k**, Patient-level DA error rate on TCGA before and after activating CP. **l**, Patient-level DA error rate on CPTAC before and after activating CP. For **a–h**, **k** and **l**, data are presented as mean ± 95% CI (error bands for **k**, **l**) across *n* = 20 independently trained models, with statistical significance assessed using a one-sided Wilcoxon signed-rank test (**a**-**h**). Models were evaluated using a combination of calibration and testing data, with panels **e**-**l** specifically incorporating 500 CP evaluations per model (see Methods for details). DA, definitive answer.

**Fig. 3 |:**
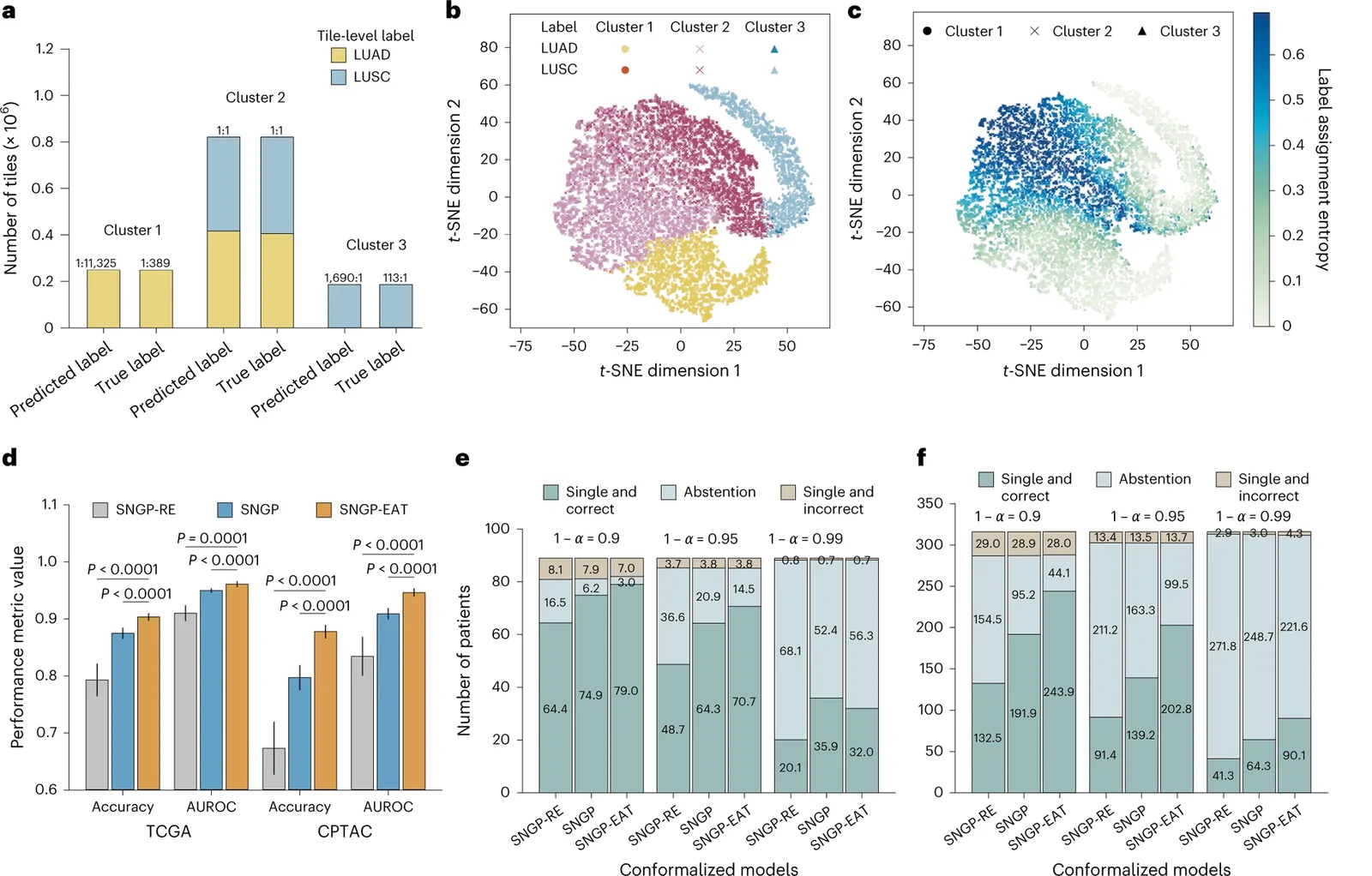
Evaluation of EAT on NSCLC subtyping using Inception-v3 as the backbone. **a**, The count and ratio of tiles predicted by SNGP as LUSC versus LUAD within each tile cluster identified using *k*-means on SNGP-based tile representations, along with the corresponding true labels derived from slide-level diagnosis. **b**, *t*-SNE-based illustration of tile clusters and true labels. **c**, Tile-wise ambiguity measured by label assignment entropy on top of the *t*-SNE visualization. For the sake of illustration, we randomly sampled 10,000 tiles to visualize the *t*-SNE distributions in **b** and **c**. **d**, Patient-level classification performance on the TCGA and CPTAC testing datasets. **e**, Patient-level classification breakdown of three conformalized SNGP-based models on TCGA. **f**, Patient-level classification breakdown on CPTAC. For **d**, data are presented as mean ± 95% CI based on *n*=20 independently trained models, each with 500 CP evaluations (see details in Methods), with statistical significance assessed using a one-sided Wilcoxon signed-rank test. RE, random elimination.

**Fig. 4 |:**
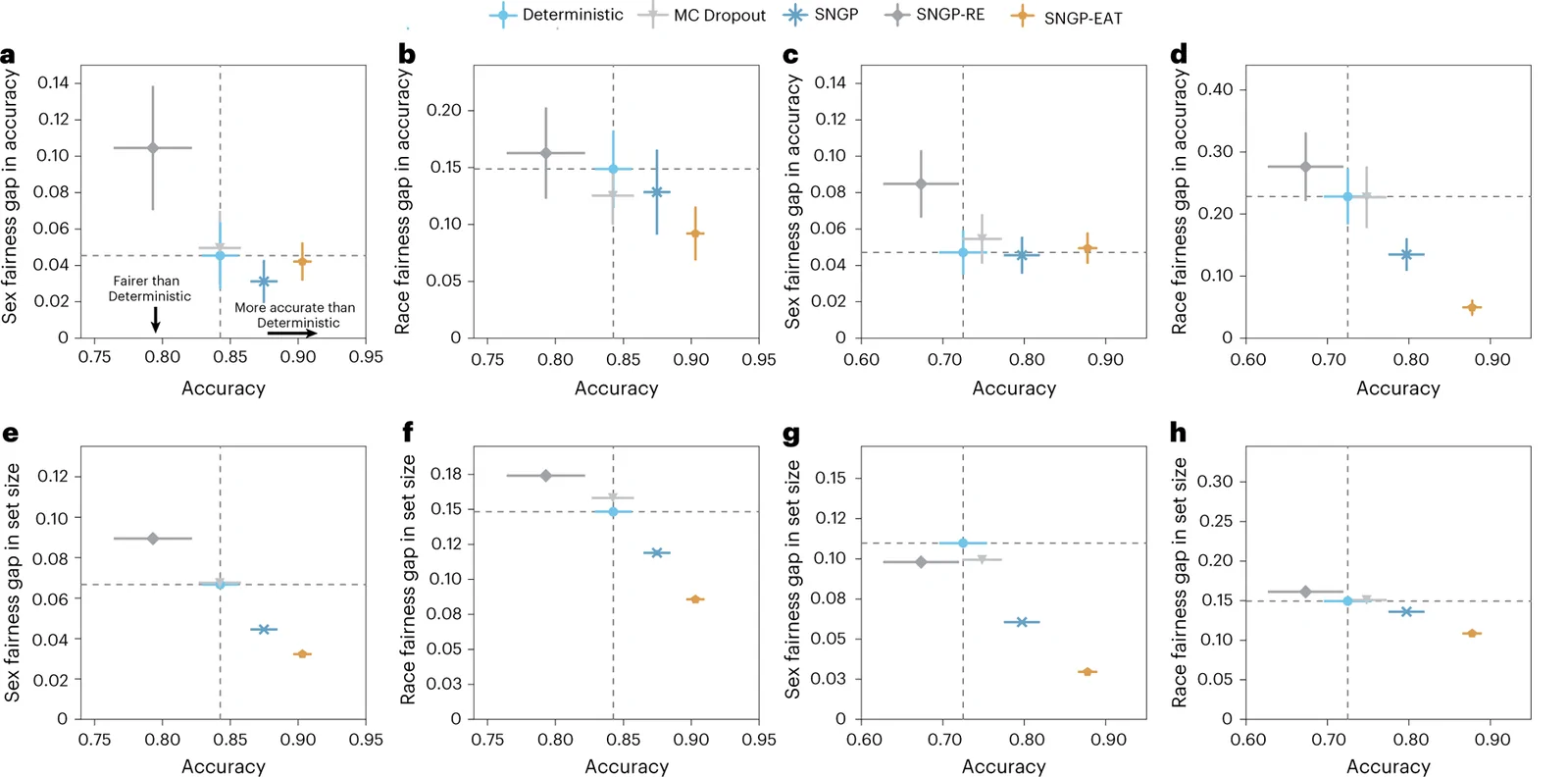
Fairness evaluation of TRUECAM based on metric value differences between the best- and worst-performing demographic subgroups against overall accuracy of NSCLC subtyping. **a**-**d**, Sex-wise (**a**,**c**) and race-wise (**b**,**d**) accuracy gap versus overall accuracy for discriminating between LUAD and LUSC on TCGA (**a**,**b**; *n* = 189) and CPTAC (**c**,**d**; *n* = 416) with CP deactivated. Data are presented as mean ± 95% CI based on 20 independently trained models. **e**-**h**, At α=0.1, sex-wise (**e**,**g**) and race-wise (**f**,**h**) set size gap versus overall accuracy on TCGA (**e**,**f**) and CPTAC (**g**,**h**) with CP activated. For **a**-**h**, data are presented as mean ± 95% CI based on 20 independently trained models, each with 500 CP evaluations. Evaluations on Deterministic or its conformalized version are marked as an overall baseline. Additional results are provided in Supplementary Tables 13-20.

**Fig. 5 |:**
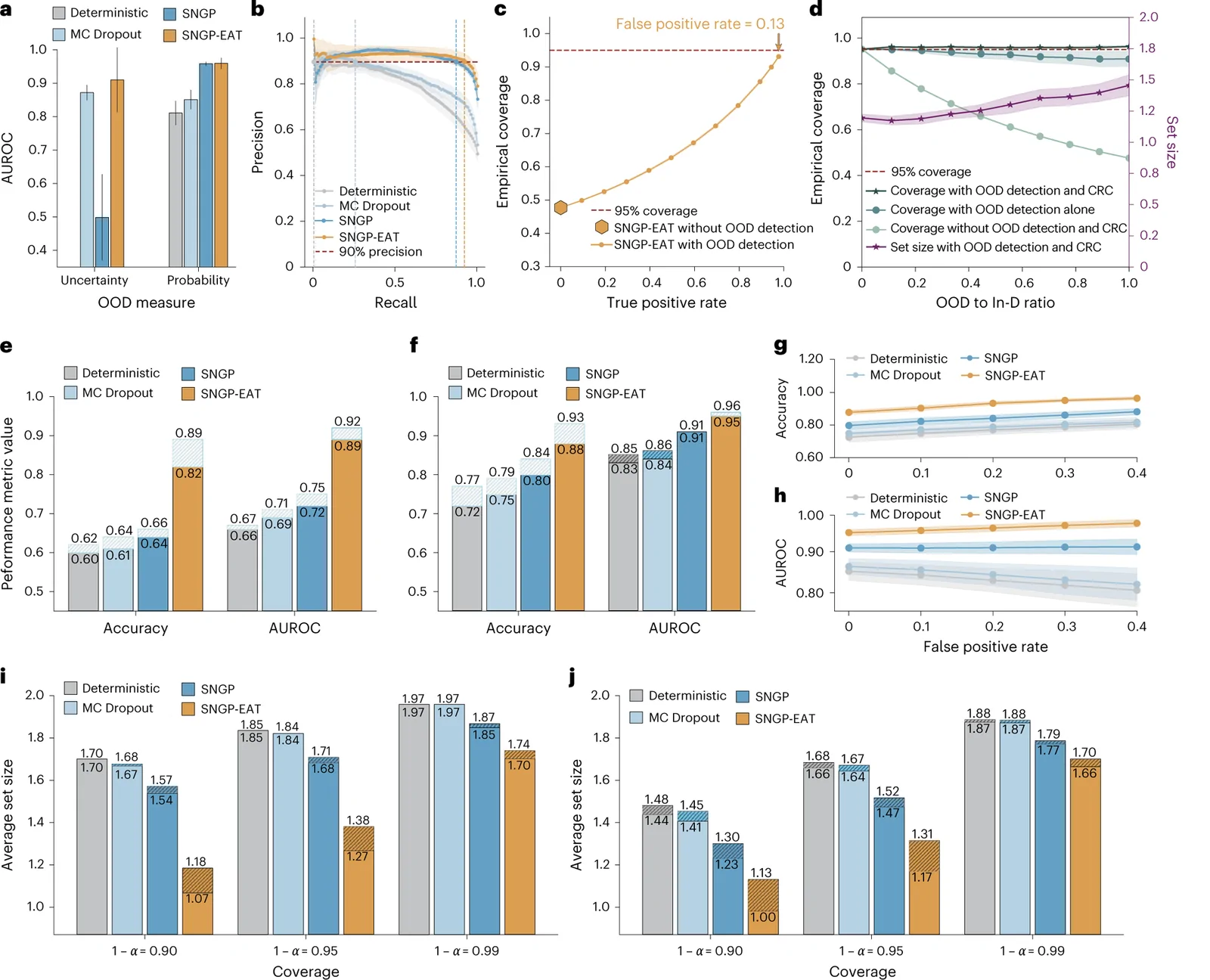
Evaluation of OOD detection enabled by TRUECAM. **a**, Performance of two distinct OOD scores in identifying slides outside a model’s scope, using the top δ=20 tiles in a slide with the lowest tile-level values for patient-level OOD score calculation. Data are presented as mean ± 95% CI based on *n* = 20 independent runs. The bar for Deterministic is not shown as it does not measure uncertainty. See Supplementary Table 22 for sensitivity analyses for varying δ values. **b**, Precision-recall curves for OOD detection using the OOD measure that performs best for each method shown in **a**. **c**, Empirical model coverage with α=0.05 as a function of the TPR for OOD detection. **d**, Empirical coverage and prediction set size of CP for TRUECAM, compared to the scenario where CRC and OOD detection were deactivated, shown as a function of the ratio of OOD to In-D data. **e**, Impact assessment of DSC on the tile-level classification performance. Striped boxes in blue and black indicate performance increase and decrease, respectively, compared to the scenario without DSC. **f**, Impact assessment of DSC on the patient-level classification performance. **g**,**h**, Accuracy (**g**) and AUROC (**h**) for evaluation on CPTAC as a function of FPR in OOD detection. **i**, Impact assessment of DSC on the tile-level average set size. Striped boxes in black indicate improvement in set size, compared to the scenario without DSC. **j**, Impact assessment of DSC on the patient-level average set size.

**Fig. 6 |:**
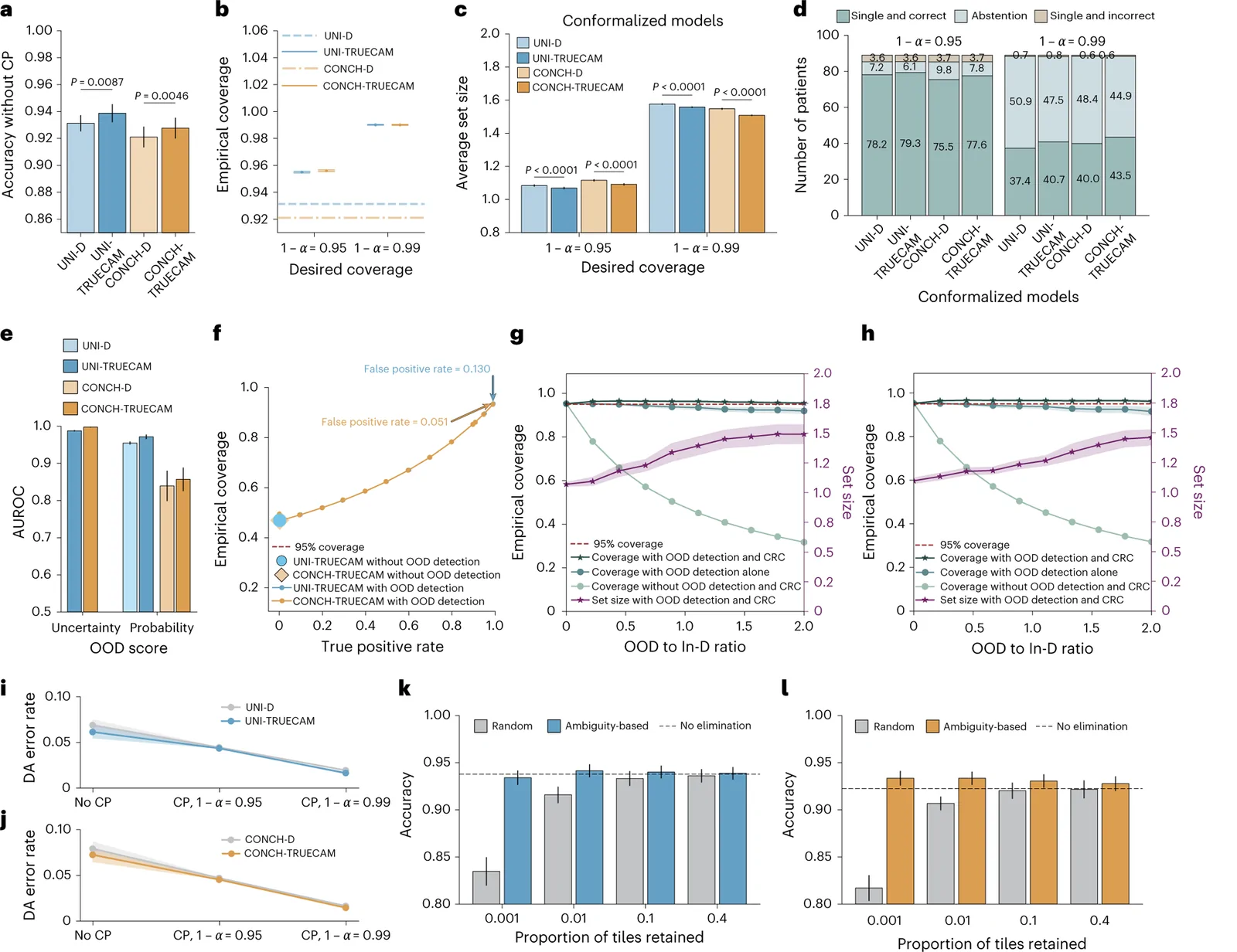
Assessment of TRUECAM using the foundation models UNI and CONCH. Results on TCGA (*n* = 189) are reported. See Extended Data Fig. 4 for results on CPTAC, which produced similar observations. **a**, Classification accuracy of foundation model-based ABMIL with (denoted using suffix ’-TRUECAM’) and without TRUECAM (that is, the original version of foundation model-based ABMIL, denoted using suffix ’-D’), evaluated without CP (*n* = 20 independent runs). **b**, Comparison of empirical coverage with and without TRUECAM (*n* = 20). **c**, Comparison of average set size of CP with and without TRUECAM (*n* = 20). **d**, Comparison of patient classification breakdown with and without TRUECAM. **e**, Performance of two distinct OOD scores in identifying slides outside a model’s scope. The uncertainty-based score is not applicable to the original versions of UNI and CONCH (*n*=20). **f**, Empirical coverage with α=0.05 as a function of the TPR for OOD detection. **g**,**h**, Empirical coverage and set size of CP for UNI-TRUECAM (**g**) and CONCH-TRUECAM (**h**) under CRC with a FPR of 0.130 for UNI and 0.051 for CONCH, shown as a function of OOD-to-In-D ratio. **i**, **j**, Comparison of patient-level DA error rate with and without TRUECAM for UNI (**i**) and CONCH (**j**) across varying contexts of CP. **k**,**l**, Comparison of classification accuracy for UNI-TRUECAM (**k**) and CONCH-TRUECAM (**l**) using TRUECAM’s ambiguity-based tile elimination strategy versus random tile elimination, shown as a function of the proportion of tiles retained per slide (*n* = 20). For **a**-**c**, **e**, **k** and **l**, data are presented as mean ± 95% CI across *n* = 20 independently trained models, with statistical significance assessed using a one-sided Wilcoxon signed-rank test for **a** and **c**. For **g** and **l**, error bands are presented as mean ± 95% CI across *n* = 20 independently trained models.

## Data Availability

The TCGA diagnostic whole-slide data and corresponding labels are available from the NIH genomic data commons (https://portal.gdc.cancer.gov). The CPTAC whole-slide data and the corresponding labels are available from the NIH cancer imaging archive (https://cancerim-agingarchive.net/datascope/cptac). Non-cancerous lung tissue WSIs for the OOD detection task are available from the TCGA project portal (https://portal.gdc.cancer.gov). The BRACS data can be downloaded from the Institute for High Performance Computing and Networking (ICAR) (https://bracs.icar.cnr.it/). We summarized the links to all the data used in this paper in Supplementary Table 47. The in-house QMH dataset is not publicly available owing to patient privacy regulations and institutional review board requirements. Source data are provided with this paper.

## Code availability

All code was implemented in Python using PyTorch as the primary deep-learning library. The complete pipeline for processing WSIs as well as training and evaluating models is available under the MIT License at https://github.com/iamownt/TRUECAM and can be used to reproduce the experiments of this paper.
