# Supplementary material for: Implementing trust in non-small cell lung cancer diagnosis with a conformalized uncertainty-aware AI framework
Source: Nat Biomed Eng. Author manuscript; Available in PMC 2026 Oct 29. (PMC13717466; doi:10.1038/s41551-026-01694-8)
Supplement: Supplementary file 1 [file NIHMS2209222-supplement-supplementary_Material.pdf]

nature biomedical engineering

Supplementary information https://doi.org/10.1038/s41551-026-01694-8

Implementing trust in non-small cell lung cancer diagnosis with a conformalized uncertainty-aware AI framework

In the format provided by the authors and unedited

Dataset	Category	Subcategories	Num. Slides	Num. Patients
TCGA (n=941)	Race	White	678	678
		Not reported	165	165
		Black or African American	80	80
		Asian	17	17
		American Indian or Alaska Native	1	1
	Sex	Male	569	569
		Female	372	372
CPTAC (n=416)	Race	White	768	244
		Asian	390	123
		Other	120	37
		Black or African American	10	6
		Unknown	9	3
		Not reported	8	2
		American Indian or Alaska Native	1	1
	Sex	Male	959	288
		Female	344	127
		Not reported	3	1

Supplementary Table 1: **An overview of the public NSCLC datasets used in this study along with a summary of the associated patient demographic information.**

Dataset	Tissue site	Target	Label level	Num. Classes	Num. Patients	Num. Slides
QMH-NSCLC	Lung	Subtype	Patient	2	1,337	3,123
TCGA-OT	Pan-cancer	OncoTree	WSI	46	9,149	11,186
TCGA-BRTS	Brain	Subtype	Patient	3	617	1,225
BRACS	Breast	Subtype (coarse)	WSI	3	189	547
TCGA-BRCA	Breast	Subtype	Patient	2	984	1,049
TCGA-RCC	Kidney	OncoTree	Patient	3	895	925

Supplementary Table 2: **An overview of the cancer subtyping datasets used to evaluate the generalizability and scalability of TRUECAM.**

Hyperparameter	Value
Hidden layers (top)	2
Hidden layer dimension (top)	1024
Pooling (top)	Average
Dropout	0.1
Tile px	299
Effective magnification	10X
Random horizontal flip prob	0.5
Random vertical flip prob	0.5
Random 90-degree rotation prob	0.5
Random JPEG compression (quality 50-100) prob	0.5
Random Gaussian blur (sigma 0.5-2.0) prob	0.1
Adam, β	(0.9, 0.999)
Batch size	64
Max epochs	4
Weight decay	None
Early stopping monitor	Accuracy
Learning rate scheduler	ExponentialDecay
Initial learning rate	0.0003
Learning rate decay steps	512
Learning rate decay rate	0.98
Automatic mixed precision	tf32

Supplementary Table 3: **Hyperparameters used in training deep learning models built upon the Inception-v3 architecture.** Deterministic, MC Dropout, SNGP share the same training protocols and pre-processing steps. For VBLL, SN-VBLL utilizes features from the trained SNGP model, while VBLL uses features from the trained deterministic model's final layer.

Hyperparameter	Value
Tile px	256
Effective magnification	20X
Batch size	1
Weight decay	1e-2
AdamW β	(0.9, 0.999)
Initial learning rate	1e-4
Learning rate schedule	CosineAnnealingLR
Epochs	Up to 20

Supplementary Table 4: **Hyperparameters used in training MIL models on top of pathology foundation models.**

Hyperparameter	Value
Num inducing	1024
GP scale	1.0
GP bias	0.
GP kernel type	Gaussian
GP input normalization	False
GP cov discount factor	-1
GP cov ridge penalty	1.0
GP output bias trainable	False
GP scale random features	False
GP random feature type	ORF

Supplementary Table 5: **Hyperparameters used in the random Fourier feature approximated Gaussian process in SNGP for the Inception-v3 architecture**. The implementation is based on `tfm.nlp.layers.RandomFeatureGaussianProcess`.

Hyperparameter	Value
Num inducing	1024
GP scale	1.0
GP bias	0.
GP kernel type	Gaussian
GP input normalization	True
GP cov discount factor	-1
GP cov ridge penalty	1.0
GP output bias trainable	False
GP scale random features	False
GP random feature type	ORF

Supplementary Table 6: **Hyperparameters used in the random Fourier feature approximated Gaussian process in UNI-SNGP**. The implementation is based on `tfm.nlp.layers.RandomFeatureGaussianProcess`.

Hyperparameter	Value
Num inducing	512 (CONCH), 768 (TITAN) 1024 (Prov-GigaPath)
GP scale	1.0
GP bias	0.
GP kernel type	Linear
GP input normalization	False
GP cov discount factor	-1
GP cov ridge penalty	1.0
GP output bias trainable	True
GP scale random features	False
GP random feature type	ORF

Supplementary Table 7: **Hyperparameters used in the random Fourier feature approximated Gaussian process in CONCH-SNGP, Prov-GigaPath, TITAN**. The implementation is based on `tfm.nlp.layers.RandomFeatureGaussianProcess`.

Model	Dataset	Tile level		Patient level	
		Accuracy	AUROC	Accuracy	AUROC
Deterministic	TCGA	0.7312 ± 0.0069	0.8138 ± 0.0072	0.8426 ± 0.0133	0.9263 ± 0.0096
MC Dropout		0.7321 ± 0.0076	0.8130 ± 0.0079	0.8423 ± 0.0154	0.9234 ± 0.0089
VBLL (Generative)		0.7340 ± 0.0077	0.8221 ± 0.0085	0.8580 ± 0.0094	0.9346 ± 0.0088
VBLL (Discriminative)		0.7339 ± 0.0081	0.8220 ± 0.0085	0.8606 ± 0.0104	0.9347 ± 0.0087
SN-VBLL (Generative)		0.7482 ± 0.0061	0.8376 ± 0.0067	0.8687 ± 0.0091	0.9425 ± 0.0048
SN-VBLL (Discriminative)		0.7469 ± 0.0064	0.8373 ± 0.0067	0.8682 ± 0.0092	0.9424 ± 0.0048
SNGP		0.7437 ± 0.0079	0.8356 ± 0.0068	0.8748 ± 0.0098	0.9496 ± 0.0041
SNGP-RE		0.7056 ± 0.0175	0.7838 ± 0.0112	0.7930 ± 0.0287	0.9099 ± 0.0138
SNGP-EAT		**0.9234 ± 0.0086**	**0.9462 ± 0.0098**	**0.9031 ± 0.0066**	**0.9608 ± 0.0053**
Deterministic	CPTAC	0.5981 ± 0.0192	0.6563 ± 0.0282	0.7249 ± 0.0291	0.8522 ± 0.0220
MC Dropout		0.6134 ± 0.0188	0.6852 ± 0.0221	0.7481 ± 0.0241	0.8640 ± 0.0187
VBLL (Generative)		0.6018 ± 0.0201	0.6773 ± 0.0293	0.7600 ± 0.0215	0.8659 ± 0.0231
VBLL (Discriminative)		0.6013 ± 0.0201	0.6761 ± 0.0294	0.7583 ± 0.0225	0.8652 ± 0.0231
SN-VBLL (Generative)		0.6424 ± 0.0110	0.7247 ± 0.0146	0.7916 ± 0.0167	0.8985 ± 0.0081
SN-VBLL (Discriminative)		0.6420 ± 0.0113	0.7242 ± 0.0148	0.7917 ± 0.0171	0.8981 ± 0.0080
SNGP		0.6386 ± 0.0117	0.7214 ± 0.0158	0.7971 ± 0.0219	0.9090 ± 0.0096
SNGP-RE		0.5556 ± 0.0260	0.6236 ± 0.0402	0.6732 ± 0.0463	0.8344 ± 0.0342
SNGP-EAT		**0.8250 ± 0.0328**	**0.8949 ± 0.0182**	**0.8776 ± 0.0117**	**0.9461 ± 0.0070**

Supplementary Table 8: **Performance comparison of Inception-v3-based models developed using TCGA training data and evaluated on TCGA test data and external CPTAC data**. The best-performing model for each dataset is highlighted in bold. Results are presented as the mean ± 95% confidence intervals, derived from 20 random data splits and corresponding model training sessions.

Model	Evaluation dataset	Average set size		
		$\alpha = 0.10$	$\alpha = 0.05$	$\alpha = 0.01$
Deterministic	TCGA	1.4008 ± 0.0007*	1.6529 ± 0.0010*	1.9246 ± 0.0003*
MC Dropout		1.3965 ± 0.0008*	1.6432 ± 0.0011*	1.9218 ± 0.0003*
SNGP		1.3600 ± 0.0007*	1.5313 ± 0.0006*	1.7731 ± 0.0006*
SNGP-RE		1.4314 ± 0.0011*	1.6039 ± 0.0010*	1.8332 ± 0.0007*
SNGP-EAT		**0.9640 ± 0.0005***	**1.0982 ± 0.0017***	**1.6234 ± 0.0020***
Deterministic	CPTAC	1.7025 ± 0.0011*	1.8495 ± 0.0006*	1.9695 ± 0.0001*
MC Dropout		1.6764 ± 0.0011*	1.8364 ± 0.0006*	1.9668 ± 0.0001*
SNGP		1.5711 ± 0.0010*	1.7084 ± 0.0009*	1.8678 ± 0.0006*
SNGP-RE		1.6742 ± 0.0015*	1.7849 ± 0.0013*	1.9079 ± 0.0009*
SNGP-EAT		**1.1841 ± 0.0030***	**1.3806 ± 0.0031***	**1.7410 ± 0.0014***

Supplementary Table 9: **Tile-level average set size with CP applied to Inception-v3-based models across various error levels (α).** In few cases, the average set size is less than 1. This is because the DA error rate of the baseline model is lower than the desired value. In this situation, the model rejects and outputs a zero set to achieve exact coverage. When employing CP in the real-world setting, this can be turned off. The best-performing model for each setting is highlighted in bold. Results are presented as the mean ± 95% confidence intervals, derived from 20 random data splits and corresponding model training sessions, with each including 500 conformal prediction evaluations based on random partitions of the calibration and testing datasets. * indicates that the corresponding model achieves the target coverage (e.g., $1 - \alpha$).

Model	Evaluation dataset	Average set size		
		$\alpha = 0.10$	$\alpha = 0.05$	$\alpha = 0.01$
Deterministic	TCGA	1.1456 ± 0.0017*	1.3620 ± 0.0024*	1.6676 ± 0.0033*
MC Dropout		1.1506 ± 0.0017*	1.3582 ± 0.0021*	1.6671 ± 0.0032
SNGP		1.0698 ± 0.0013*	1.2424 ± 0.0018*	1.5919 ± 0.0034*
SNGP-RE		1.1900 ± 0.0023*	1.4156 ± 0.0031*	1.7661 ± 0.0035*
SNGP-EAT		**1.0119 ± 0.0011***	**1.1709 ± 0.0020***	**1.6364 ± 0.0042***
Deterministic	CPTAC	1.4789 ± 0.0029*	1.6843 ± 0.0023*	1.8848 ± 0.0015
MC Dropout		1.4521 ± 0.0030*	1.6702 ± 0.0025*	1.8815 ± 0.0016*
SNGP		1.3007 ± 0.0026*	1.5166 ± 0.0026*	1.7869 ± 0.0024*
SNGP-RE		1.4891 ± 0.0030*	1.6685 ± 0.0027*	1.8603 ± 0.0020*
SNGP-EAT		**1.1312 ± 0.0021***	**1.3148 ± 0.0027***	**1.7011 ± 0.0038***

Supplementary Table 10: **Patient-level average set size with CP applied to Inception-v3-based models across various error levels (α).** The best-performing model for each setting is highlighted in bold. Results are presented as the mean ± 95% confidence intervals, derived from 20 random data splits and corresponding model training sessions, with each including 500 conformal prediction evaluations based on random partitions of the calibration and testing datasets. * indicates that the corresponding model achieves the target coverage (e.g., $1 - \alpha$).

Model	Evaluation dataset	DA error rate			
		No CP	$\alpha = 0.10$	$\alpha = 0.05$	$\alpha = 0.01$
Deterministic	TCGA	0.2688 ± 0.0069	0.1673 ± 0.0002	0.1468 ± 0.0004	0.1372 ± 0.0005
MC Dropout		0.2679 ± 0.0076	0.1663 ± 0.0002	0.1432 ± 0.0004	0.1328 ± 0.0005
SNGP		0.2563 ± 0.0079	0.1566 ± 0.0002	0.1071 ± 0.0002	0.0446 ± 0.0001
SNGP-RE		0.2944 ± 0.0175	0.1775 ± 0.0003	0.1281 ± 0.0003	0.0628 ± 0.0003
SNGP-EAT		**0.0766 ± 0.0086**	**0.0632 ± 0.0004**	**0.0553 ± 0.0001**	**0.0287 ± 0.0002**
Deterministic	CPTAC	0.4019 ± 0.0192	0.3488 ± 0.0014	0.3462 ± 0.0014	0.3431 ± 0.0015
MC Dropout		0.3866 ± 0.0188	0.3189 ± 0.0012	0.3154 ± 0.0012	0.3113 ± 0.0011
SNGP		0.3614 ± 0.0116	0.2369 ± 0.0006	0.1765 ± 0.0007	0.0819 ± 0.0006
SNGP-RE		0.4444 ± 0.0260	0.3259 ± 0.0017	0.2592 ± 0.0019	0.1577 ± 0.0022
SNGP-EAT		**0.1750 ± 0.0328**	**0.1219 ± 0.0006**	**0.0858 ± 0.0004**	**0.0448 ± 0.0005**

Supplementary Table 11: **Tile-level DA error rate before and after applying CP to Inception-v3-based models across various error levels (α).** The best-performing model for each setting is highlighted in bold. Results are presented as the mean ± 95% confidence intervals, derived from 20 random data splits and corresponding model training sessions, with each including 500 conformal prediction evaluations based on random partitions of the calibration and testing datasets. In the "No CP" settings, only one evaluation is performed for each trained model.

Model	Evaluation dataset	DA error rate			
		No CP	$\alpha = 0.10$	$\alpha = 0.05$	$\alpha = 0.01$
Deterministic	TCGA	0.1574 ± 0.0133	0.1070 ± 0.0008	0.0659 ± 0.0007	0.0277 ± 0.0009
MC Dropout		0.1577 ± 0.0154	0.1070 ± 0.0008	0.0647 ± 0.0007	0.0389 ± 0.0024
SNGP		0.1252 ± 0.0098	0.0961 ± 0.0007	0.0558 ± 0.0007	**0.0190 ± 0.0005**
SNGP-RE		0.2070 ± 0.0288	0.1135 ± 0.0009	0.0730 ± 0.0008	0.0524 ± 0.0027
SNGP-EAT		0.0968 ± 0.0067	0.0810 ± 0.0006	**0.0508 ± 0.0006**	0.0232 ± 0.0012
Deterministic	CPTAC	0.2751 ± 0.0291	0.1866 ± 0.0013	0.1495 ± 0.0016	0.0913 ± 0.0026
MC Dropout		0.2519 ± 0.0241	0.1760 ± 0.0010	0.1399 ± 0.0011	0.0725 ± 0.0012
SNGP		0.2029 ± 0.0219	0.1337 ± 0.0007	0.0914 ± 0.0007	**0.0518 ± 0.0021**
SNGP-RE		0.3268 ± 0.0463	0.1931 ± 0.0013	0.1454 ± 0.0014	0.1214 ± 0.0037
SNGP-EAT		**0.1224 ± 0.0117**	**0.1039 ± 0.0006**	**0.0645 ± 0.0005**	0.0535 ± 0.0029

Supplementary Table 12: **Patient-level DA error rate before and after applying CP to Inception-v3-based models across various error levels (α).** The best-performing model for each setting is highlighted in bold. Results are presented as the mean ± 95% confidence intervals, derived from 20 random data splits and corresponding model training sessions, with each including 500 conformal prediction evaluations based on random partitions of the calibration and testing datasets. In the "No CP" settings, only one evaluation is performed for each trained model.

Model	Metric	Female	Male	Fairness gap
Deterministic	Accuracy	0.8347 ± 0.0228	0.8472 ± 0.0158	0.0454 ± 0.0183
MC Dropout		0.8268 ± 0.0240	0.8517 ± 0.0184	0.0496 ± 0.0203
SNGP		0.8753 ± 0.0148	0.8746 ± 0.0125	**0.0312 ± 0.0117**
SNGP-RE		0.7629 ± 0.0474	0.8114 ± 0.0325	0.1045 ± 0.0341
SNGP-EAT		0.9278 ± 0.0127	0.8895 ± 0.0065	0.0421 ± 0.0104
Deterministic	AUROC	0.9240 ± 0.0171	0.9168 ± 0.0110	0.0286 ± 0.0099
MC Dropout		0.9209 ± 0.0148	0.9144 ± 0.0119	0.0358 ± 0.0089
SNGP		0.9535 ± 0.0088	0.9400 ± 0.0073	**0.0231 ± 0.0081**
SNGP-RE		0.9199 ± 0.0200	0.8959 ± 0.0154	0.0388 ± 0.0141
SNGP-EAT		0.9731 ± 0.0064	0.9483 ± 0.0087	0.0270 ± 0.0090

Supplementary Table 13: **Sexual fairness gaps of Inception-v3-based models in classification performance on the TCGA testing set.** The best-performing model for each dataset is highlighted in bold. Results are presented as the mean ± 95% confidence intervals, derived from 20 random data splits and corresponding model training sessions.

Model	Metric	Female	Male	Fairness gap
Deterministic	Accuracy	0.7413 ± 0.0417	0.7167 ± 0.0248	0.0471 ± 0.0120
MC Dropout		0.7693 ± 0.0381	0.7378 ± 0.0198	0.0545 ± 0.0136
SNGP		0.8232 ± 0.0289	0.7849 ± 0.0200	**0.0456 ± 0.0101**
SNGP-RE		0.6614 ± 0.0732	0.6776 ± 0.0363	0.0848 ± 0.0184
SNGP-EAT		0.9122 ± 0.0139	0.8627 ± 0.0118	0.0495 ± 0.0086
Deterministic	AUROC	0.8865 ± 0.0180	0.8330 ± 0.0247	0.0536 ± 0.0094
MC Dropout		0.8954 ± 0.0158	0.8459 ± 0.0219	0.0495 ± 0.0126
SNGP		0.9371 ± 0.0095	0.8941 ± 0.0108	0.0430 ± 0.0068
SNGP-RE		0.8699 ± 0.0306	0.8137 ± 0.0376	0.0562 ± 0.0138
SNGP-EAT		0.9693 ± 0.0041	0.9358 ± 0.0088	**0.0336 ± 0.0074**

Supplementary Table 14: **Sexual fairness gaps of Inception-v3-based models in classification performance on the CPTAC testing set.** The best-performing model for each dataset is highlighted in bold. Results are presented as the mean ± 95% confidence intervals, derived from 20 random data splits and corresponding model training sessions.

Model	Metric	Not reported	Others	White	Fairness gap
Deterministic	Accuracy	0.9057 ± 0.0266	0.7906 ± 0.0433	0.8350 ± 0.0129	0.1487 ± 0.0338
MC Dropout		0.9012 ± 0.0210	0.7879 ± 0.0329	0.8345 ± 0.0167	0.1253 ± 0.0254
SNGP		0.9302 ± 0.0192	0.8117 ± 0.0315	0.8691 ± 0.0117	0.1284 ± 0.0372
SNGP-RE		0.8738 ± 0.0294	0.7602 ± 0.0411	0.7776 ± 0.0372	0.1627 ± 0.0400
SNGP-EAT		0.9340 ± 0.0183	0.8505 ± 0.0203	0.9025 ± 0.0075	**0.0921 ± 0.0235**
Deterministic	AUROC	0.9414 ± 0.0240	0.8805 ± 0.0312	0.9271 ± 0.0098	0.1018 ± 0.0235
MC Dropout		0.9343 ± 0.0269	0.8863 ± 0.0293	0.9232 ± 0.0096	0.0941 ± 0.0263
SNGP		0.9557 ± 0.0186	0.9129 ± 0.0230	0.9522 ± 0.0054	**0.0753 ± 0.0235**
SNGP-RE		0.9165 ± 0.0347	0.8765 ± 0.0263	0.9160 ± 0.0140	0.1023 ± 0.0220
SNGP-EAT		0.9752 ± 0.0092	0.8987 ± 0.0411	0.9672 ± 0.0066	0.0859 ± 0.0408

Supplementary Table 15: **Racial fairness gaps of Inception-v3-based models in classification performance on the TCGA testing set.** The best-performing model for each dataset is highlighted in bold. Results are presented as the mean ± 95% confidence intervals, derived from 20 random data splits and corresponding model training sessions.

Model	Metric	Asian	Others	White	Fairness gap
Deterministic	Accuracy	0.5756 ± 0.0572	0.7224 ± 0.0359	0.8006 ± 0.0182	0.2282 ± 0.0444
MC Dropout		0.6012 ± 0.0571	0.7531 ± 0.0358	0.8211 ± 0.0132	0.2272 ± 0.0494
SNGP		0.7126 ± 0.0386	0.8061 ± 0.0331	0.8379 ± 0.0150	0.1348 ± 0.0260
SNGP-RE		0.5276 ± 0.0938	0.6520 ± 0.0659	0.7508 ± 0.0363	0.2763 ± 0.0551
SNGP-EAT		0.8707 ± 0.0218	0.8673 ± 0.0167	0.8832 ± 0.0107	**0.0496 ± 0.0121**
Deterministic	AUROC	0.9286 ± 0.0162	0.8793 ± 0.0213	0.8568 ± 0.0133	0.0795 ± 0.0138
MC Dropout		0.9311 ± 0.0139	0.9003 ± 0.0192	0.8652 ± 0.0110	0.0733 ± 0.0111
SNGP		0.9468 ± 0.0097	0.9398 ± 0.0139	0.8950 ± 0.0113	0.0622 ± 0.0099
SNGP-RE		0.9253 ± 0.0185	0.8546 ± 0.0407	0.8519 ± 0.0279	0.0956 ± 0.0247
SNGP-EAT		0.9629 ± 0.0074	0.9492 ± 0.0103	0.9321 ± 0.0089	**0.0360 ± 0.0059**

Supplementary Table 16: **Racial fairness gaps of Inception-v3-based models in classification performance on the CPTAC testing set.** The best-performing model for each dataset is highlighted in bold. Results are presented as the mean ± 95% confidence intervals, derived from 20 random data splits and corresponding model training sessions.

**Model**	α	**Average set size across racial groups**			
		Not reported	Others	White	Fairness gap
Deterministic	0.10	1.0871 ± 0.0017	1.1790 ± 0.0030	1.1565 ± 0.0018	0.1483 ± 0.0019
MC Dropout		1.0800 ± 0.0015	1.1795 ± 0.0029	1.1660 ± 0.0019	0.1582 ± 0.0020
SNGP		1.0252 ± 0.0009	1.0970 ± 0.0026	1.0775 ± 0.0015	0.1190 ± 0.0018
SNGP-RE		1.1059 ± 0.0021	1.1964 ± 0.0032	1.2116 ± 0.0026	0.1741 ± 0.0022
SNGP-EAT		1.0048 ± 0.0009	1.0187 ± 0.0022	1.0132 ± 0.0012	**0.0857 ± 0.0015**
Deterministic	0.05	1.2273 ± 0.0026	1.4198 ± 0.0040	1.3890 ± 0.0026	0.2636 ± 0.0027
MC Dropout		1.2135 ± 0.0025	1.4152 ± 0.0034	1.3886 ± 0.0023	0.2716 ± 0.0027
SNGP		1.1151 ± 0.0017	1.3280 ± 0.0033	1.2631 ± 0.0020	0.2528 ± 0.0026
SNGP-RE		1.2471 ± 0.0033	1.4367 ± 0.0039	1.4573 ± 0.0033	0.2809 ± 0.0025
SNGP-EAT		1.0879 ± 0.0019	1.2307 ± 0.0032	1.1842 ± 0.0021	**0.1896 ± 0.0022**
Deterministic	0.01	1.4889 ± 0.0042	1.7339 ± 0.0041	1.7060 ± 0.0033	0.2950 ± 0.0027
MC Dropout		1.4916 ± 0.0041	1.7193 ± 0.0039	1.7074 ± 0.0031	0.2870 ± 0.0027
SNGP		1.3776 ± 0.0040	1.6422 ± 0.0039	1.6418 ± 0.0035	0.3339 ± 0.0027
SNGP-RE		1.6244 ± 0.0047	1.8083 ± 0.0039	1.7972 ± 0.0034	0.2353 ± 0.0028
SNGP-EAT		1.5327 ± 0.0051	1.6675 ± 0.0049	1.6600 ± 0.0042	**0.2282 ± 0.0026**

Supplementary Table 17: **Racial fairness gaps of Inception-v3-based models in average set size on the TCGA testing set with respect to three different error levels (α).** The best-performing model for each setting is highlighted in bold. Results are presented as the mean ± 95% confidence intervals, derived from 20 random data splits and corresponding model training sessions, with each including 500 conformal prediction evaluations based on random partitions of the calibration and testing datasets.

**Model**	α	**Average set size across racial groups**			
		Asian	Others	Whites	Fairness gap
Deterministic	0.10	1.5424 ± 0.0026	1.5343 ± 0.0032	1.4357 ± 0.0032	0.1495 ± 0.0011
MC Dropout		1.5190 ± 0.0028	1.5084 ± 0.0034	1.4071 ± 0.0032	0.1509 ± 0.0013
SNGP		1.3659 ± 0.0031	1.3590 ± 0.0034	1.2561 ± 0.0023	0.1361 ± 0.0013
SNGP-RE		1.5155 ± 0.0029	1.5880 ± 0.0032	1.4559 ± 0.0033	0.1613 ± 0.0015
SNGP-EAT		1.1891 ± 0.0029	1.1444 ± 0.0026	1.0993 ± 0.0016	**0.1086 ± 0.0013**
Deterministic	0.05	1.7269 ± 0.0018	1.7492 ± 0.0026	1.6498 ± 0.0027	**0.1320 ± 0.0011**
MC Dropout		1.7236 ± 0.0019	1.7401 ± 0.0027	1.6293 ± 0.0029	0.1465 ± 0.0012
SNGP		1.5813 ± 0.0026	1.6113 ± 0.0032	1.4650 ± 0.0026	0.1664 ± 0.0012
SNGP-RE		1.6834 ± 0.0025	1.7766 ± 0.0025	1.6393 ± 0.0031	0.1649 ± 0.0015
SNGP-EAT		1.3958 ± 0.0034	1.3739 ± 0.0034	1.2621 ± 0.0025	0.1638 ± 0.0015
Deterministic	0.01	1.8942 ± 0.0013	1.9482 ± 0.0014	1.8673 ± 0.0017	**0.0934 ± 0.0009**
MC Dropout		1.8890 ± 0.0012	1.9452 ± 0.0014	1.8650 ± 0.0019	0.0942 ± 0.0010
SNGP		1.7961 ± 0.0021	1.9032 ± 0.0021	1.7590 ± 0.0027	0.1531 ± 0.0012
SNGP-RE		1.8629 ± 0.0019	1.9397 ± 0.0014	1.8431 ± 0.0024	0.1111 ± 0.0014
SNGP-EAT		1.7445 ± 0.0036	1.7739 ± 0.0037	1.6646 ± 0.0041	0.1387 ± 0.0015

Supplementary Table 18: **Racial fairness gaps of Inception-v3-based models in average set size on the CPTAC testing set with respect to three different error levels (α).** The best-performing model for each setting is highlighted in bold. Results are presented as the mean ± 95% confidence intervals, derived from 20 random data splits and corresponding model training sessions, with each including 500 conformal prediction evaluations based on random partitions of the calibration and testing datasets.

**Model**	α	**Average set size across sexual groups**		
		Female	Male	Fairness gap
Deterministic	0.10	1.1509 ± 0.0020	1.1424 ± 0.0018	0.0667 ± 0.0011
MC Dropout		1.1588 ± 0.0021	1.1453 ± 0.0018	0.0676 ± 0.0012
SNGP		1.0644 ± 0.0015	1.0730 ± 0.0014	0.0445 ± 0.0008
SNGP-RE		1.2215 ± 0.0030	1.1715 ± 0.0022	0.0894 ± 0.0015
SNGP-EAT		1.0118 ± 0.0013	1.0120 ± 0.0011	**0.0323 ± 0.0006**
Deterministic	0.05	1.3772 ± 0.0029	1.3533 ± 0.0026	0.1011 ± 0.0015
MC Dropout		1.3774 ± 0.0027	1.3455 ± 0.0023	0.1031 ± 0.0015
SNGP		1.2445 ± 0.0023	1.2410 ± 0.0019	0.0797 ± 0.0012
SNGP-RE		1.4525 ± 0.0036	1.3933 ± 0.0031	0.1128 ± 0.0016
SNGP-EAT		1.1764 ± 0.0021	1.1676 ± 0.0021	**0.0613 ± 0.0010**
Deterministic	0.01	1.7011 ± 0.0035	1.6489 ± 0.0034	0.0978 ± 0.0014
MC Dropout		1.6920 ± 0.0036	1.6526 ± 0.0032	0.0888 ± 0.0013
SNGP		1.6168 ± 0.0037	1.5773 ± 0.0035	0.0872 ± 0.0013
SNGP-RE		1.7777 ± 0.0036	1.7584 ± 0.0037	**0.0767 ± 0.0013**
SNGP-EAT		1.6168 ± 0.0042	1.6480 ± 0.0044	0.0880 ± 0.0013

Supplementary Table 19: **Sexual fairness gaps of Inception-v3-based models in average set size on the TCGA testing set with respect to three different error levels (α).** The best-performing model for each setting is highlighted in bold. Results are presented as the mean ± 95% confidence intervals, derived from 20 random data splits and corresponding model training sessions, with each including 500 conformal prediction evaluations based on random partitions of the calibration and testing datasets.

**Model**	α	**Average set size across sexual groups**		
		Female	Male	Fairness gap
Deterministic	0.10	1.5483 ± 0.0035	1.4497 ± 0.0027	0.1098 ± 0.0012
MC Dropout		1.5188 ± 0.0035	1.4242 ± 0.0028	0.0994 ± 0.0012
SNGP		1.3279 ± 0.0033	1.2893 ± 0.0024	0.0606 ± 0.0009
SNGP-RE		1.5405 ± 0.0036	1.4667 ± 0.0029	0.0980 ± 0.0012
SNGP-EAT		1.1277 ± 0.0021	1.1327 ± 0.0021	**0.0296 ± 0.0005**
Deterministic	0.05	1.7508 ± 0.0024	1.6562 ± 0.0024	0.1002 ± 0.0010
MC Dropout		1.7382 ± 0.0025	1.6414 ± 0.0025	0.0980 ± 0.0010
SNGP		1.5687 ± 0.0032	1.4942 ± 0.0025	0.0881 ± 0.0010
SNGP-RE		1.7060 ± 0.0029	1.6515 ± 0.0027	0.0786 ± 0.0010
SNGP-EAT		1.3076 ± 0.0031	1.3169 ± 0.0026	**0.0451 ± 0.0006**
Deterministic	0.01	1.9135 ± 0.0012	1.8721 ± 0.0017	0.0463 ± 0.0008
MC Dropout		1.9120 ± 0.0012	1.8682 ± 0.0017	0.0464 ± 0.0008
SNGP		1.8179 ± 0.0022	1.7733 ± 0.0025	0.0525 ± 0.0008
SNGP-RE		1.8784 ± 0.0019	1.8519 ± 0.0022	**0.0368 ± 0.0007**
SNGP-EAT		1.6981 ± 0.0041	1.7014 ± 0.0038	0.0488 ± 0.0007

Supplementary Table 20: **Sexual fairness gaps of Inception-v3-based models in terms of average set size on the CPTAC testing set with respect to three different error levels (α).** The best-performing model for each setting is highlighted in bold. Results are presented as the mean ± 95% confidence intervals, derived from 20 random data splits and corresponding model training sessions, with each including 500 conformal prediction evaluations based on random partitions of the calibration and testing datasets.

Model	Architecture	Uncertainty-based OOD score		Probability-based OOD score	
		AUROC	AUPR	AUROC	AUPR
**Patch-Level Models: Cancerous Lung vs. Non-cancerous Lung (TCGA-NSCLC vs. OOD)**					
Deterministic	Inception-v3	-	-	0.8109 ± 0.0361	0.7948 ± 0.0307
MC Dropout	Inception-v3	0.8721 ± 0.0232	0.8322 ± 0.0223	0.8510 ± 0.0290	0.8209 ± 0.0241
SNGP	Inception-v3	0.4985 ± 0.1290	0.4638 ± 0.0673	0.9584 ± 0.0061	0.9192 ± 0.0129
SNGP-EAT	Inception-v3	0.9102 ± 0.0973	0.8610 ± 0.0790	**0.9596 ± 0.0167**	**0.9253 ± 0.0337**
**Foundation Models: Cancerous Lung vs. Non-cancerous Lung (TCGA-NSCLC vs. OOD)**					
Deterministic	UNI	-	-	0.9507 ± 0.0041	0.9071 ± 0.0093
SNGP	UNI	**0.9859 ± 0.0015**	**0.9790 ± 0.0048**	0.9755 ± 0.0038	0.9561 ± 0.0077
SNGP-EAT	UNI	0.9834 ± 0.0015	0.9754 ± 0.0048	0.9673 ± 0.0060	0.9408 ± 0.0139
Deterministic	CONCH	-	-	0.8359 ± 0.0405	0.7629 ± 0.0389
SNGP	CONCH	**0.9936 ± 0.0013**	**0.9861 ± 0.0047**	0.8529 ± 0.0307	0.7696 ± 0.0356
SNGP-EAT	CONCH	0.9933 ± 0.0013	0.9855 ± 0.0045	0.8535 ± 0.0315	0.7744 ± 0.0365
Deterministic	Prov-GigaPath	-	-	0.8833 ± 0.0223	0.8296 ± 0.0324
SNGP	Prov-GigaPath	**0.9597 ± 0.0156**	**0.9544 ± 0.0222**	0.8833 ± 0.0223	0.8296 ± 0.0324
SNGP-EAT	Prov-GigaPath	0.9398 ± 0.0214	0.9317 ± 0.0276	0.8983 ± 0.0191	0.8506 ± 0.0252
Deterministic	TITAN	-	-	0.8799 ± 0.0163	0.7593 ± 0.0299
SNGP	TITAN	0.9921 ± 0.0016	**0.9885 ± 0.0030**	0.8799 ± 0.0163	0.7593 ± 0.0299
SNGP-EAT	TITAN	**0.9932 ± 0.0017**	0.9876 ± 0.0042	0.8883 ± 0.0125	0.7599 ± 0.0218
**Foundation Models: Cancerous Lung vs. Non-cancerous Lung (CPTAC-NSCLC vs. OOD)**					
Deterministic	UNI	-	-	0.9853 ± 0.0015	0.9861 ± 0.0032
SNGP	UNI	0.9943 ± 0.0021	0.9961 ± 0.0018	0.9834 ± 0.0034	0.9857 ± 0.0044
SNGP-EAT	UNI	**0.9967 ± 0.0011**	**0.9981 ± 0.0007**	0.9827 ± 0.0037	0.9834 ± 0.0051
Deterministic	CONCH	-	-	0.9272 ± 0.0201	0.9441 ± 0.0146
SNGP	CONCH	**0.9971 ± 0.0007**	**0.9984 ± 0.0004**	0.9765 ± 0.0056	0.9791 ± 0.0041
SNGP-EAT	CONCH	0.9961 ± 0.0008	0.9978 ± 0.0005	0.9862 ± 0.0048	0.9892 ± 0.0032
Deterministic	Prov-GigaPath	-	-	0.9333 ± 0.0141	0.9567 ± 0.0110
SNGP	Prov-GigaPath	**0.9748 ± 0.0102**	**0.9831 ± 0.0080**	0.9333 ± 0.0141	0.9567 ± 0.0110
SNGP-EAT	Prov-GigaPath	0.9729 ± 0.0105	0.9819 ± 0.0082	0.9339 ± 0.0144	0.9571 ± 0.0122
Deterministic	TITAN	-	-	0.9503 ± 0.0000	0.9621 ± 0.0000
SNGP	TITAN	**0.9961 ± 0.0000**	**0.9975 ± 0.0000**	0.9503 ± 0.0000	0.9621 ± 0.0000
SNGP-EAT	TITAN	0.9960 ± 0.0001	**0.9975 ± 0.0001**	0.9496 ± 0.0008	0.9527 ± 0.0007

Supplementary Table 21: **Patient-level OOD detection performance using two distinct OOD scores, evaluated on non-cancerous lung tissue slides from the TCGA project.** UNI and CONCH are paired with attention-based multi-instance learning (ABMIL) mechanism for slide inference, whereas Prov-GigaPath and TITAN utilize their own slide encoders, as suggested by [30] and [31]. For Prov-GigaPath and TITAN, we appended a Gaussian process layer to the slide-level representations without applying spectral normalization, as the representations extracted from these models inherently preserve distances effectively, due to their dedicated self-supervised training strategy. For reference convenience, SNGP-EAT for UNI, CONCH, Prov-GigaPath, and TITAN in the OOD detection setting are referenced as UNI-TRUECAM, CONCH-TRUECAM, Prov-GigaPath-TRUECAM, and TITAN-TRUECAM in the main text, respectively. Deterministic models (i.e., those without TRUECAM) can only use probability-based OOD scores, given the absence of dedicated uncertainty components. The best-performing model for each dataset is highlighted in bold. Results are presented as the mean ± 95% confidence intervals, derived from 20 random data splits and corresponding model training sessions.

Model	δ	Uncertainty-based OOD score		Probability-based OOD score	
		AUROC	AUPR	AUROC	AUPR
Deterministic	20	-	-	0.8109 ± 0.0361	0.7948 ± 0.0307
MC Dropout	20	0.8721 ± 0.0232	0.8322 ± 0.0223	0.8510 ± 0.0290	0.8209 ± 0.0241
SNGP	20	0.4985 ± 0.1290	0.4638 ± 0.0673	0.9584 ± 0.0061	0.9192 ± 0.0129
SNGP-EAT	20	0.9102 ± 0.0973	0.8610 ± 0.0790	**0.9596 ± 0.0167**	**0.9253 ± 0.0337**
Deterministic	40	-	-	0.8214 ± 0.0271	0.7867 ± 0.0276
MC Dropout	40	0.8682 ± 0.0213	0.8177 ± 0.0235	0.8525 ± 0.0236	0.8076 ± 0.0247
SNGP	40	0.5028 ± 0.1167	0.4573 ± 0.0594	0.9509 ± 0.0068	0.9039 ± 0.0142
SNGP-EAT	40	0.9019 ± 0.0956	0.8421 ± 0.0794	**0.9570 ± 0.0163**	**0.9287 ± 0.0311**
Deterministic	60	-	-	0.8248 ± 0.0227	0.7791 ± 0.0256
MC Dropout	60	0.8626 ± 0.0209	0.8064 ± 0.0242	0.8501 ± 0.0226	0.7973 ± 0.0255
SNGP	60	0.5058 ± 0.1087	0.4538 ± 0.0545	0.9451 ± 0.0073	0.8917 ± 0.0150
SNGP-EAT	60	0.8959 ± 0.0940	0.8282 ± 0.0789	**0.9539 ± 0.0159**	**0.9277 ± 0.0290**
Deterministic	80	-	-	0.8247 ± 0.0214	0.7719 ± 0.0243
MC Dropout	80	0.8575 ± 0.0203	0.7972 ± 0.0245	0.8478 ± 0.0213	0.7895 ± 0.0254
SNGP	80	0.5086 ± 0.1025	0.4515 ± 0.0507	0.9401 ± 0.0076	0.8808 ± 0.0153
SNGP-EAT	80	0.8905 ± 0.0920	0.8149 ± 0.0780	**0.9513 ± 0.0154**	**0.9272 ± 0.0273**
Deterministic	100	-	-	0.8242 ± 0.0207	0.7639 ± 0.0231
MC Dropout	100	0.8542 ± 0.0199	0.7884 ± 0.0246	0.8451 ± 0.0209	0.7805 ± 0.0254
SNGP	100	0.5110 ± 0.0976	0.4501 ± 0.0478	0.9353 ± 0.0080	0.8720 ± 0.0152
SNGP-EAT	100	0.8860 ± 0.0899	0.8027 ± 0.0767	**0.9484 ± 0.0155**	**0.9253 ± 0.0270**

Supplementary Table 22: **Impact assessment of the number of top δ tiles on the OOD detection performance of Inception-v3-based models, measured by AUROC and AUPR.** The TCGAtesting datasets was used as the In-D data, while TCGA-NSCLC-STN was used as the OOD data. The best-performing model for each setting is highlighted in bold. SNGP-EAT with δ=20 achieved the highest performance in OOD detection.

Model	Classification level	Without DSC control		With DSC control	
		Accuracy	AUROC	Accuracy	AUROC
Deterministic	Tile	0.5981 ± 0.0192	0.6563 ± 0.0282	0.6188 ± 0.0235	0.6744 ± 0.0311
MC Dropout	Tile	0.6134 ± 0.0188	0.6852 ± 0.0221	0.6363 ± 0.0239	0.7078 ± 0.0238
SNGP	Tile	0.6386 ± 0.0117	0.7214 ± 0.0158	0.6643 ± 0.0150	0.7490 ± 0.0176
SNGP-EAT	Tile	0.8250 ± 0.0328	0.8949 ± 0.0182	**0.8873 ± 0.0221**	**0.9198 ± 0.0137**
Deterministic	Patient	0.7249 ± 0.0291	0.8522 ± 0.0220	0.7703 ± 0.0270	0.8303 ± 0.0278
MC Dropout	Patient	0.7481 ± 0.0241	0.8640 ± 0.0187	0.7878 ± 0.0307	0.8441 ± 0.0263
SNGP	Patient	0.7971 ± 0.0219	0.9090 ± 0.0096	0.8411 ± 0.0199	0.9095 ± 0.0137
SNGP-EAT	Patient	0.8776 ± 0.0117	0.9461 ± 0.0070	**0.9337 ± 0.0109**	**0.9572 ± 0.0081**

Supplementary Table 23: **Impact assessment of OOD detection-based distribution shift control (DSC) on the classification performance in terms of accuracy and AUROC with respect to the external CPTAC dataset when the FPR for OOD detection was set at 0.2.** The results in this table correspond to Fig. 5e,f. Distribution shift control achieved a significant boost in the NSCLC subtyping accuracy and AUROC when applying models trained on TCGA to CPTAC, and bridged the performance gap due to distribution shift. See Supplementary Table 8 for model performance evaluated on TCGA. The best-performing model for each setting is highlighted in bold, and results are presented with mean ± 95% CI from 20 experimental replicates.

Model	Classification level	α	Without DSC control	With DSC control
			Average set size	Average set size
Deterministic	Tile	0.10	1.7025 ± 0.0011	1.7011 ± 0.0011
MC Dropout		0.10	1.6764 ± 0.0011	1.6747 ± 0.0011
SNGP		0.10	1.5711 ± 0.0010	1.5378 ± 0.0012
SNGP-EAT		0.10	1.1841 ± 0.0030	**1.0676 ± 0.0022**
Deterministic		0.05	1.8495 ± 0.0006	1.8493 ± 0.0006
MC Dropout		0.05	1.8364 ± 0.0006	1.8357 ± 0.0006
SNGP		0.05	1.7084 ± 0.0009	1.6825 ± 0.0011
SNGP-EAT		0.05	1.3806 ± 0.0031	**1.2670 ± 0.0031**
Deterministic		0.01	1.9695 ± 0.0001	1.9696 ± 0.0001
MC Dropout		0.01	1.9668 ± 0.0001	1.9668 ± 0.0001
SNGP		0.01	1.8678 ± 0.0006	1.8549 ± 0.0007
SNGP-EAT		0.01	1.7410 ± 0.0014	**1.7016 ± 0.0018**
Deterministic	Patient	0.10	1.4789 ± 0.0029	1.4377 ± 0.0032
MC Dropout		0.10	1.4521 ± 0.0030	1.4062 ± 0.0033
SNGP		0.10	1.3007 ± 0.0026	1.2307 ± 0.0029
SNGP-EAT		0.10	1.1312 ± 0.0021	**0.9812 ± 0.0015**
Deterministic		0.05	1.6843 ± 0.0023	1.6590 ± 0.0025
MC Dropout		0.05	1.6702 ± 0.0025	1.6443 ± 0.0027
SNGP		0.05	1.5166 ± 0.0026	1.4741 ± 0.0029
SNGP-EAT		0.05	1.3148 ± 0.0027	**1.1702 ± 0.0033**
Deterministic		0.01	1.8848 ± 0.0015	1.8748 ± 0.0016
MC Dropout		0.01	1.8815 ± 0.0016	1.8713 ± 0.0017
SNGP		0.01	1.7869 ± 0.0024	1.7728 ± 0.0026
SNGP-EAT		0.01	1.7011 ± 0.0038	**1.6638 ± 0.0047**

Supplementary Table 24: **Impact assessment of OOD detection-based distribution shift control (DSC) on the CP performance in terms of average set size with respect to the external CPTAC dataset when the FPR for OOD detection was set at 0.2.** The results in this table correspond to Fig. 5i,j. Distribution shift control achieved a significant boost in the NSCLC subtyping average set size when applying models trained on TCGA to CPTAC, and bridged the performance gap due to distribution shift. See Supplementary Table 9 for the tile-level set size of the trained Inception-v3 model on the TCGA test data. The best-performing model for each setting is highlighted in bold, and results are presented with mean ± 95% CI from 20 experimental replicates, with each including 500 conformal prediction evaluations based on random partitions of the calibration and testing datasets.

Model	Evaluation dataset	SN	Accuracy	AUROC
UNI	TCGA	None	0.9313 ± 0.0060	**0.9747 ± 0.0034**
UNI-SNGP	TCGA	2.0	0.9377 ± 0.0003	0.9734 ± 0.0001
UNI-SNGP-EAT	TCGA	2.0	**0.9382 ± 0.0003**	0.9737 ± 0.0001
CONCH	TCGA	None	0.9210 ± 0.0077	0.9729 ± 0.0041
CONCH-SNGP	TCGA	2.0	0.9224 ± 0.0089	0.9747 ± 0.0038
CONCH-SNGP-EAT	TCGA	2.0	**0.9277 ± 0.0003**	**0.9762 ± 0.0001**
Prov-GigaPath	TCGA	None	0.9310 ± 0.0065	0.9791 ± 0.0028
Prov-GigaPath-EAT	TCGA	None	**0.9342 ± 0.0072**	**0.9797 ± 0.0029**
TITAN	TCGA	None	0.9329 ± 0.0064	**0.9767 ± 0.0028**
TITAN-EAT	TCGA	None	**0.9376 ± 0.0056**	0.9766 ± 0.0030
UNI	CPTAC	None	0.9464 ± 0.0028	0.9753 ± 0.0006
UNI-SNGP	CPTAC	2.0	0.9458 ± 0.0038	0.9774 ± 0.0013
UNI-SNGP-EAT	CPTAC	2.0	**0.9470 ± 0.0001**	**0.9786 ± 0.0001**
CONCH	CPTAC	None	0.9470 ± 0.0028	0.9732 ± 0.0041
CONCH-SNGP	CPTAC	2.0	0.9476 ± 0.0028	**0.9836 ± 0.0013**
CONCH-SNGP-EAT	CPTAC	2.0	**0.9524 ± 0.0000**	0.9821 ± 0.0001
Prov-GigaPath	CPTAC	None	**0.9411 ± 0.0078**	0.9789 ± 0.0026
Prov-GigaPath-EAT	CPTAC	None	**0.9411 ± 0.0072**	**0.9800 ± 0.0025**
TITAN	CPTAC	None	**0.9643 ± 0.0000**	0.9747 ± 0.0000
TITAN-EAT	CPTAC	None	**0.9643 ± 0.0000**	**0.9765 ± 0.0004**

Supplementary Table 25: **Main classification results of foundation models for the NSCLC subtyping task**. For Prov-GigaPath and TITAN, we do not enforce spectral normalization due to the following reasons: 1) Prov-GigaPath employs a two-stage pretraining strategy, and enforcing spectral normalization in the slide encoder (LongNet) could negatively impact the pretrained weights; 2) TITAN leverages linear probing on the slide embeddings without further fine-tuning the slide encoder, making the impose of spectral normalization unnecessary. These observations suggest that TRUECAM is well modularized and its modules can be effectively integrated into various models. For simplicity, we set the mask tile threshold to 0.4 for all models in TCGA and CPTAC. The exception is the CPTAC cohort of Prov-GigaPath and TITAN, where the threshold is set to 0.8 in our experiments. More results on ablation study of mask tile threshold can be seen in Supplementary Table 35- 36. Note that TITAN-preview is abbreviated as TITAN throughout this paper. SN: spectral norm.

Model	Subtyping Task	Balanced Accuracy	Weighted F1
Inception-v3	TCGA-OT	0.3333 ± 0.0124	0.4176 ± 0.0133
Inception-v3-TRUECAM	TCGA-OT	**0.4052 ± 0.0180**	**0.4483 ± 0.0141**
Inception-v3	QMH-NSCLC	0.8140 ± 0.0368	0.8931 ± 0.0200
Inception-v3-TRUECAM	QMH-NSCLC	**0.8859 ± 0.0321**	**0.9263 ± 0.0171**
Inception-v3	TCGA-BRTS	0.6940 ± 0.0447	0.8090 ± 0.0338
Inception-v3-TRUECAM	TCGA-BRTS	**0.7375 ± 0.0358**	**0.8351 ± 0.0190**
Inception-v3	BRACS-Coarse	0.5176 ± 0.0247	**0.5785 ± 0.0606**
Inception-v3-TRUECAM	BRACS-Coarse	**0.5198 ± 0.0159**	0.5727 ± 0.0612
Inception-v3	BRCA	0.6162 ± 0.0341	0.7958 ± 0.0255
Inception-v3-TRUECAM	BRCA	**0.8262 ± 0.0328**	**0.8827 ± 0.0151**
Inception-v3	TCGA-RCC	0.9180 ± 0.0250	0.9353 ± 0.0210
Inception-v3-TRUECAM	TCGA-RCC	**0.9408 ± 0.0268**	**0.9412 ± 0.0186**

Supplementary Table 26: **Performance evaluation of Inception-v3 with and without TRUECAM on five public and one private (QMH-NSCLC) benchmarks.** The best-performing model for each dataset is highlighted in bold. Results are presented as the mean ± 95% confidence intervals, derived from five random data splits and corresponding model training sessions. Performance on QMH-NSCLC and TCGA-OT are presented as the mean ± standard deviation of 150 and 1000 bootstrap trials, respectively.

Model	MIL Type	Accuracy	AUROC
UNI	ABMIL	0.9313 ± 0.0060	**0.9747 ± 0.0034**
UNI-TRUECAM	ABMIL	**0.9382 ± 0.0003**	0.9737 ± 0.0001
UNI	CLAM	**0.9347 ± 0.0061**	**0.9735 ± 0.0042**
UNI-TRUECAM	CLAM	**0.9347 ± 0.0056**	0.9712 ± 0.0043
UNI	TransMIL	0.9236 ± 0.0077	0.9740 ± 0.0034
UNI-TRUECAM	TransMIL	**0.9271 ± 0.0088**	**0.9741 ± 0.0048**
CONCH	ABMIL	0.9210 ± 0.0077	0.9729 ± 0.0041
CONCH-TRUECAM	ABMIL	**0.9277 ± 0.0003**	**0.9762 ± 0.0001**
CONCH	CLAM	0.9239 ± 0.0076	0.9745 ± 0.0041
CONCH-TRUECAM	CLAM	**0.9298 ± 0.0089**	**0.9760 ± 0.0039**
CONCH	TransMIL	0.9145 ± 0.0089	0.9707 ± 0.0047
CONCH-TRUECAM	TransMIL	**0.9371 ± 0.0075**	**0.9711 ± 0.0048**
Prov-GigaPath	N/A	0.9310 ± 0.0065	0.9791 ± 0.0028
Prov-GigaPath-TRUECAM	N/A	**0.9342 ± 0.0072**	**0.9797 ± 0.0029**
TITAN	N/A	0.9329 ± 0.0064	**0.9767 ± 0.0028**
TITAN-TRUECAM	N/A	**0.9376 ± 0.0056**	0.9766 ± 0.0030

Supplementary Table 27: **Performance evaluation of pathology foundation models with and without TRUECAM on the TCGA-NSCLC subtyping task.** The best-performing model for each setting is highlighted in bold, and results are presented with mean ± 95% CI from 20 experimental replicates. For the slide-level foundation model setting, we fine-tuned the slide encoder of Prov-GigaPath and performed linear probing on the slide representations of TITAN for downstream classification tasks as suggested by [30] and [31].

Model	MIL Type	Accuracy	AUROC
UNI	ABMIL	0.9464 ± 0.0028	0.9753 ± 0.0006
UNI-TRUECAM	ABMIL	**0.9470 ± 0.0001**	**0.9786 ± 0.0001**
UNI	CLAM	**0.9494 ± 0.0025**	0.9768 ± 0.0013
UNI-TRUECAM	CLAM	0.9464 ± 0.0043	**0.9820 ± 0.0015**
UNI	TransMIL	0.9405 ± 0.0031	**0.9721 ± 0.0021**
UNI-TRUECAM	TransMIL	**0.9458 ± 0.0038**	0.9672 ± 0.0043
CONCH	ABMIL	0.9470 ± 0.0028	0.9732 ± 0.0041
CONCH-TRUECAM	ABMIL	**0.9524 ± 0.0000**	**0.9821 ± 0.0001**
CONCH	CLAM	0.9464 ± 0.0034	0.9702 ± 0.0043
CONCH-TRUECAM	CLAM	**0.9482 ± 0.0033**	**0.9746 ± 0.0027**
CONCH	TransMIL	0.9411 ± 0.0038	**0.9798 ± 0.0019**
CONCH-TRUECAM	TransMIL	**0.9458 ± 0.0029**	0.9769 ± 0.0026
Prov-GigaPath	N/A	**0.9411 ± 0.0078**	0.9789 ± 0.0026
Prov-GigaPath-TRUECAM	N/A	**0.9411 ± 0.0072**	**0.9800 ± 0.0025**
TITAN	N/A	**0.9643 ± 0.0000**	0.9747 ± 0.0000
TITAN-TRUECAM	N/A	**0.9643 ± 0.0000**	**0.9765 ± 0.0004**

Supplementary Table 28: **Performance evaluation of pathology foundation models with and without TRUECAM on the CPTAC-NSCLC subtyping task.** The best-performing model for each setting is highlighted in bold, and results are presented with mean ± 95% CI from 20 experimental replicates. For the slide-level foundation model setting, we fine-tuned the slide encoder of Prov-GigaPath and performed linear probing on the slide representations of TITAN for downstream classification tasks as suggested by [30] and [31].

Model	MIL Type	Balanced Accuracy	Weighted F1
UNI	ABMIL	0.9955 ± 0.0032	0.9927 ± 0.0052
UNI-TRUECAM	ABMIL	**0.9977 ± 0.0023**	**0.9963 ± 0.0037**
UNI	CLAM	0.9932 ± 0.0042	0.9889 ± 0.0068
UNI-TRUECAM	CLAM	**0.9979 ± 0.0022**	**0.9965 ± 0.0036**
UNI	TransMIL	0.9911 ± 0.0043	0.9855 ± 0.0069
UNI-TRUECAM	TransMIL	**0.9933 ± 0.0035**	**0.9891 ± 0.0057**
CONCH	ABMIL	0.9606 ± 0.0190	0.9782 ± 0.0091
CONCH-TRUECAM	ABMIL	**0.9716 ± 0.0167**	**0.9818 ± 0.0085**
CONCH	CLAM	0.9761 ± 0.0161	**0.9891 ± 0.0065**
CONCH-TRUECAM	CLAM	**0.9769 ± 0.0125**	0.9774 ± 0.0093
CONCH	TransMIL	0.9553 ± 0.0220	0.9820 ± 0.0084
CONCH-TRUECAM	TransMIL	**0.9815 ± 0.0121**	**0.9847 ± 0.0077**
Prov-GigaPath	N/A	0.9956 ± 0.0032	0.9929 ± 0.0052
Prov-GigaPath-TRUECAM	N/A	**0.9979 ± 0.0022**	**0.9965 ± 0.0036**
TITAN	N/A	0.9743 ± 0.0157	0.9856 ± 0.0076
TITAN-TRUECAM	N/A	**0.9842 ± 0.0113**	**0.9891 ± 0.0064**

Supplementary Table 29: **Performance evaluation of pathology foundation models with and without TRUECAM on the QMH-NSCLC subtyping task.** The best-performing model for each model is highlighted in bold. Results are presented as the mean ± standard deviation of 150 bootstrap trials. For the slide-level foundation model setting, we fine-tuned the slide encoder of Prov-GigaPath and performed linear probing on the slide representations of TITAN for downstream classification tasks as suggested by [30] and [31].

Model	MIL Type	Balanced Accuracy	Weighted F1
UNI	ABMIL	0.6332 ± 0.0157	0.6974 ± 0.0135
UNI-TRUECAM	ABMIL	**0.6334 ± 0.0201**	**0.7175 ± 0.0130**
UNI	CLAM	0.5792 ± 0.0165	0.7012 ± 0.0130
UNI-TRUECAM	CLAM	**0.5929 ± 0.0167**	**0.7128 ± 0.0127**
UNI	TransMIL	0.6233 ± 0.0175	**0.7354 ± 0.0128**
UNI-TRUECAM	TransMIL	**0.6342 ± 0.0160**	0.7207 ± 0.0129
CONCH	ABMIL	0.6205 ± 0.0204	0.7260 ± 0.0131
CONCH-TRUECAM	ABMIL	**0.6277 ± 0.0191**	**0.7278 ± 0.0130**
CONCH	CLAM	0.6270 ± 0.0188	0.7224 ± 0.0130
CONCH-TRUECAM	CLAM	**0.6338 ± 0.0165**	**0.7302 ± 0.0130**
CONCH	TransMIL	0.6228 ± 0.0162	0.7332 ± 0.0126
CONCH-TRUECAM	TransMIL	**0.6442 ± 0.0189**	**0.7418 ± 0.0129**
Prov-GigaPath	N/A	**0.6696 ± 0.0169**	0.7496 ± 0.0126
Prov-GigaPath-TRUECAM	N/A	0.6585 ± 0.0206	**0.7609 ± 0.0125**
TITAN	N/A	0.6924 ± 0.0204	0.7584 ± 0.0121
TITAN-TRUECAM	N/A	**0.6973 ± 0.0207**	**0.7620 ± 0.0121**

Supplementary Table 30: **Performance comparison of pathology foundation models on the TCGA-OT pan-cancer slide-level classification task with 46 classes.** The best-performing model for each model is highlighted in bold. Results are presented as the mean ± standard deviation of 1000 bootstrap trials. For the slide-level foundation model setting, we fine-tuned the slide encoder of Prov-GigaPath and performed linear probing on the slide representations of TITAN for downstream classification tasks as suggested by [30] and [31].

Model	MIL Type	Balanced Accuracy	Weighted F1
UNI	ABMIL	0.8080 ± 0.0672	0.8756 ± 0.0416
UNI-TRUECAM	ABMIL	**0.8213 ± 0.0694**	**0.8865 ± 0.0413**
UNI	CLAM	**0.7948 ± 0.0353**	0.8686 ± 0.0257
UNI-TRUECAM	CLAM	0.7946 ± 0.0617	**0.8703 ± 0.0363**
UNI	TransMIL	0.7728 ± 0.0468	0.8558 ± 0.0325
UNI-TRUECAM	TransMIL	**0.7915 ± 0.0596**	**0.8720 ± 0.0329**
CONCH	ABMIL	0.7736 ± 0.0417	0.8405 ± 0.0287
CONCH-TRUECAM	ABMIL	**0.7850 ± 0.0107**	**0.8524 ± 0.0067**
CONCH	CLAM	**0.7505 ± 0.0468**	0.8269 ± 0.0220
CONCH-TRUECAM	CLAM	**0.7505 ± 0.0335**	**0.8270 ± 0.0102**
CONCH	TransMIL	0.7331 ± 0.0225	0.8219 ± 0.0233
CONCH-TRUECAM	TransMIL	**0.7567 ± 0.0334**	**0.8376 ± 0.0252**
Prov-GigaPath	N/A	**0.8202 ± 0.0565**	**0.8859 ± 0.0353**
Prov-GigaPath-TRUECAM	N/A	0.8034 ± 0.0277	0.8789 ± 0.0157
TITAN	N/A	**0.8125 ± 0.0207**	0.8720 ± 0.0123
TITAN-TRUECAM	N/A	0.8083 ± 0.0148	**0.8735 ± 0.0045**

Supplementary Table 31: **Performance comparison of pathology foundation models on the TCGA-BRTS brain tumor subtyping task.** The best-performing model for each dataset is highlighted in bold. Results are presented as the mean ± 95% confidence intervals, derived from five random data splits and corresponding model training sessions. For the slide-level foundation model setting, we fine-tuned the slide encoder of Prov-GigaPath and performed linear probing on the slide representations of TITAN for downstream classification tasks as suggested by [30] and [31].

Model	MIL Type	Balanced Accuracy	Weighted F1
UNI	ABMIL	0.6488 ± 0.0521	0.7105 ± 0.0603
UNI-TRUECAM	ABMIL	**0.6572 ± 0.0631**	**0.7184 ± 0.0682**
UNI	CLAM	0.6713 ± 0.0639	0.7275 ± 0.0586
UNI-TRUECAM	CLAM	**0.6814 ± 0.0879**	**0.7353 ± 0.0729**
UNI	TransMIL	0.5862 ± 0.0437	0.6609 ± 0.0808
UNI-TRUECAM	TransMIL	**0.6200 ± 0.0181**	**0.6800 ± 0.0764**
CONCH	ABMIL	**0.7130 ± 0.0365**	**0.7693 ± 0.0405**
CONCH-TRUECAM	ABMIL	0.7108 ± 0.0435	0.7610 ± 0.0602
CONCH	CLAM	**0.7142 ± 0.0595**	**0.7745 ± 0.0531**
CONCH-TRUECAM	CLAM	0.7128 ± 0.0564	0.7722 ± 0.0497
CONCH	TransMIL	0.6674 ± 0.0422	0.7293 ± 0.0271
CONCH-TRUECAM	TransMIL	**0.7105 ± 0.0581**	**0.7621 ± 0.0543**
Prov-GigaPath	N/A	0.6424 ± 0.0666	0.7025 ± 0.0903
Prov-GigaPath-TRUECAM	N/A	**0.6656 ± 0.0283**	**0.7332 ± 0.0576**
TITAN	N/A	0.6172 ± 0.0447	0.6952 ± 0.0770
TITAN-TRUECAM	N/A	**0.6231 ± 0.0618**	**0.7026 ± 0.0790**

Supplementary Table 32: **Performance comparison of pathology foundation models on the BRCA coarse-grained subtyping task using the BRACS data.** The best-performing model for each dataset is highlighted in bold. Results are presented as the mean ± 95% confidence intervals, derived from five random data splits and corresponding model training sessions. For the slide-level foundation model setting, we fine-tuned the slide encoder of Prov-GigaPath and performed linear probing on the slide representations of TITAN for downstream classification tasks as suggested by [30] and [31].

Model	MIL Type	Balanced Accuracy	Weighted F1
UNI	ABMIL	0.8748 ± 0.0317	0.9188 ± 0.0130
UNI-TRUECAM	ABMIL	**0.8803 ± 0.0299**	**0.9219 ± 0.0093**
UNI	CLAM	0.8761 ± 0.0245	0.9207 ± 0.0071
UNI-TRUECAM	CLAM	**0.8833 ± 0.0252**	**0.9213 ± 0.0134**
UNI	TransMIL	0.8402 ± 0.0592	0.9073 ± 0.0223
UNI-TRUECAM	TransMIL	**0.8748 ± 0.0233**	**0.9190 ± 0.0204**
CONCH	ABMIL	0.8694 ± 0.0625	**0.9178 ± 0.0221**
CONCH-TRUECAM	ABMIL	**0.8778 ± 0.0178**	0.9157 ± 0.0138
CONCH	CLAM	0.8803 ± 0.0223	**0.9219 ± 0.0070**
CONCH-TRUECAM	CLAM	**0.8833 ± 0.0250**	0.9213 ± 0.0107
CONCH	TransMIL	**0.8864 ± 0.0268**	0.9233 ± 0.0109
CONCH-TRUECAM	TransMIL	0.8847 ± 0.0132	**0.9259 ± 0.0094**
Prov-GigaPath	N/A	0.8586 ± 0.0386	**0.9200 ± 0.0214**
Prov-GigaPath-TRUECAM	N/A	**0.8671 ± 0.0131**	**0.9200 ± 0.0057**
TITAN	N/A	0.8652 ± 0.0438	0.9198 ± 0.0257
TITAN-TRUECAM	N/A	**0.8659 ± 0.0418**	**0.9207 ± 0.0233**

Supplementary Table 33: **Performance comparison of pathology foundation models on the TCGA-BRCA breast cancer subtyping task.** The best-performing model for each dataset is highlighted in bold. Results are presented as the mean ± 95% confidence intervals, derived from five random data splits and corresponding model training sessions. For the slide-level foundation model setting, we fine-tuned the slide encoder of Prov-GigaPath and performed linear probing on the slide representations of TITAN for downstream classification tasks as suggested by [30] and [31].

Model	MIL Type	Balanced Accuracy	Weighted F1
UNI	ABMIL	0.9561 ± 0.0179	0.9509 ± 0.0127
UNI-TRUECAM	ABMIL	**0.9639 ± 0.0156**	**0.9651 ± 0.0163**
UNI	CLAM	0.9564 ± 0.0264	0.9552 ± 0.0256
UNI-TRUECAM	CLAM	**0.9650 ± 0.0124**	**0.9650 ± 0.0061**
UNI	TransMIL	0.9467 ± 0.0161	0.9446 ± 0.0197
UNI-TRUECAM	TransMIL	**0.9513 ± 0.0260**	**0.9615 ± 0.0119**
CONCH	ABMIL	0.9451 ± 0.0274	0.9431 ± 0.0184
CONCH-TRUECAM	ABMIL	**0.9491 ± 0.0201**	**0.9454 ± 0.0151**
CONCH	CLAM	0.9419 ± 0.0113	0.9411 ± 0.0088
CONCH-TRUECAM	CLAM	**0.9439 ± 0.0174**	**0.9444 ± 0.0149**
CONCH	TransMIL	0.9334 ± 0.0345	0.9399 ± 0.0176
CONCH-TRUECAM	TransMIL	**0.9457 ± 0.0191**	**0.9478 ± 0.0149**
Prov-GigaPath	N/A	0.9278 ± 0.0334	0.9409 ± 0.0233
Prov-GigaPath-TRUECAM	N/A	**0.9414 ± 0.0235**	**0.9498 ± 0.0168**
TITAN	N/A	0.9282 ± 0.0153	0.9377 ± 0.0151
TITAN-TRUECAM	N/A	**0.9288 ± 0.0137**	**0.9387 ± 0.0124**

Supplementary Table 34: **Performance comparison of pathology foundation models on the TCGA-RCC kidney cancer subtyping task.** The best-performing model for each dataset is highlighted in bold. Results are presented as the mean ± 95% confidence intervals, derived from five random data splits and corresponding model training sessions. For the slide-level foundation model setting, we fine-tuned the slide encoder of Prov-GigaPath and performed linear probing on the slide representations of TITAN for downstream classification tasks as suggested by [30] and [31].

Mask Ratio	Evaluation dataset	Random		Ambiguity	
		Accuracy	AUROC	Accuracy	AUROC
0.001	TCGA	0.8348 ± 0.0154	0.9366 ± 0.0071	0.9340 ± 0.0077	0.9681 ± 0.0040
0.01		0.9145 ± 0.0091	0.9702 ± 0.0038	**0.9411 ± 0.0070**	0.9711 ± 0.0039
0.1		0.9335 ± 0.0080	0.9730 ± 0.0034	0.9401 ± 0.0069	0.9731 ± 0.0034
0.2		0.9369 ± 0.0075	0.9734 ± 0.0031	0.9401 ± 0.0067	0.9734 ± 0.0033
0.3		0.9348 ± 0.0074	0.9734 ± 0.0032	0.9393 ± 0.0066	0.9737 ± 0.0032
0.4		0.9356 ± 0.0073	0.9734 ± 0.0031	0.9382 ± 0.0067	0.9737 ± 0.0032
0.5		0.9356 ± 0.0069	0.9735 ± 0.0031	0.9382 ± 0.0066	0.9737 ± 0.0032
0.6		0.9363 ± 0.0071	0.9732 ± 0.0032	0.9382 ± 0.0066	**0.9738 ± 0.0032**
0.7		0.9371 ± 0.0072	0.9731 ± 0.0032	0.9382 ± 0.0068	**0.9738 ± 0.0032**
0.8		0.9371 ± 0.0066	0.9732 ± 0.0032	0.9379 ± 0.0066	0.9737 ± 0.0032
0.9		0.9374 ± 0.0070	0.9734 ± 0.0031	0.9382 ± 0.0068	0.9737 ± 0.0032
No mask	TCGA	0.9377 ± 0.0069	0.9734 ± 0.0032	0.9377 ± 0.0069	0.9734 ± 0.0032
0.001	CPTAC	0.8613 ± 0.0134	0.9492 ± 0.0058	0.8833 ± 0.0088	0.9706 ± 0.0027
0.01		0.9125 ± 0.0081	0.9741 ± 0.0022	0.9161 ± 0.0064	0.9761 ± 0.0029
0.1		0.9375 ± 0.0044	0.9768 ± 0.0014	0.9381 ± 0.0064	0.9783 ± 0.0017
0.2		0.9393 ± 0.0044	0.9771 ± 0.0013	0.9429 ± 0.0053	0.9782 ± 0.0016
0.3		0.9435 ± 0.0036	0.9774 ± 0.0011	0.9435 ± 0.0040	**0.9786 ± 0.0015**
0.4		0.9446 ± 0.0033	0.9773 ± 0.0012	**0.9470 ± 0.0034**	**0.9786 ± 0.0016**
0.5		0.9458 ± 0.0038	0.9775 ± 0.0012	**0.9470 ± 0.0034**	**0.9786 ± 0.0015**
0.6		0.9458 ± 0.0038	0.9776 ± 0.0012	0.9464 ± 0.0034	**0.9786 ± 0.0015**
0.7		0.9458 ± 0.0038	0.9775 ± 0.0013	0.9464 ± 0.0034	0.9783 ± 0.0015
0.8		0.9464 ± 0.0038	0.9775 ± 0.0014	0.9458 ± 0.0038	0.9782 ± 0.0015
0.9		0.9464 ± 0.0038	0.9775 ± 0.0013	0.9458 ± 0.0038	0.9779 ± 0.0015
No mask	CPTAC	0.9458 ± 0.0038	0.9774 ± 0.0013	0.9458 ± 0.0038	0.9774 ± 0.0013

Supplementary Table 35: **Impact of mask ratio and elimination strategies on patient-level accuracy and AUROC of UNI-SNGP (no retraining) with respect to the TCGA and CPTAC test data**. In the following supplementary material, unless otherwise specified, the spectral normalization factor c of UNI-SNGP is fixed at 2.0. Best-performing model for each case is highlighted in bold, and results are presented with mean ± 95% CI from 20 experimental replicates.

Mask Ratio	Evaluation dataset	Random		Ambiguity	
		Accuracy	AUROC	Accuracy	AUROC
0.001	TCGA	0.8171 ± 0.0139	0.9064 ± 0.0091	**0.9335 ± 0.0080**	0.9704 ± 0.0054
0.01		0.9068 ± 0.0075	0.9642 ± 0.0047	**0.9335 ± 0.0072**	0.9739 ± 0.0046
0.1		0.9203 ± 0.0088	0.9729 ± 0.0042	0.9306 ± 0.0073	0.9751 ± 0.0040
0.2		0.9211 ± 0.0095	0.9740 ± 0.0042	0.9295 ± 0.0074	0.9758 ± 0.0038
0.3		0.9203 ± 0.0082	0.9739 ± 0.0041	0.9285 ± 0.0085	0.9760 ± 0.0037
0.4		0.9216 ± 0.0098	0.9741 ± 0.0041	0.9277 ± 0.0079	0.9762 ± 0.0037
0.5		0.9224 ± 0.0090	0.9739 ± 0.0040	0.9266 ± 0.0076	**0.9764 ± 0.0037**
0.6		0.9216 ± 0.0095	0.9739 ± 0.0039	0.9264 ± 0.0080	0.9761 ± 0.0037
0.7		0.9229 ± 0.0090	0.9744 ± 0.0038	0.9248 ± 0.0084	0.9761 ± 0.0037
0.8		0.9224 ± 0.0086	0.9744 ± 0.0038	0.9253 ± 0.0083	0.9758 ± 0.0037
0.9		0.9224 ± 0.0093	0.9747 ± 0.0038	0.9242 ± 0.0084	0.9753 ± 0.0038
No mask	TCGA	0.9224 ± 0.0089	0.9747 ± 0.0038	0.9224 ± 0.0089	0.9747 ± 0.0038
0.001	CPTAC	0.8679 ± 0.0148	0.9571 ± 0.0064	0.9512 ± 0.0017	0.9762 ± 0.0015
0.01		0.9327 ± 0.0058	0.9793 ± 0.0027	0.9500 ± 0.0023	0.9803 ± 0.0016
0.1		0.9446 ± 0.0052	0.9837 ± 0.0006	0.9518 ± 0.0013	0.9822 ± 0.0007
0.2		0.9440 ± 0.0044	0.9838 ± 0.0011	**0.9524 ± 0.0000**	0.9821 ± 0.0013
0.3		0.9494 ± 0.0036	0.9839 ± 0.0012	**0.9524 ± 0.0000**	0.9821 ± 0.0014
0.4		0.9500 ± 0.0029	0.9838 ± 0.0013	**0.9524 ± 0.0000**	0.9821 ± 0.0014
0.5		0.9488 ± 0.0032	0.9838 ± 0.0013	**0.9524 ± 0.0000**	0.9823 ± 0.0015
0.6		0.9476 ± 0.0028	0.9837 ± 0.0015	**0.9524 ± 0.0000**	0.9826 ± 0.0015
0.7		0.9488 ± 0.0026	0.9839 ± 0.0014	**0.9524 ± 0.0000**	0.9830 ± 0.0015
0.8		0.9482 ± 0.0027	0.9836 ± 0.0014	**0.9524 ± 0.0000**	0.9832 ± 0.0015
0.9		0.9476 ± 0.0028	0.9836 ± 0.0013	0.9506 ± 0.0021	0.9834 ± 0.0015
No mask	CPTAC	0.9476 ± 0.0028	0.9836 ± 0.0013	0.9476 ± 0.0028	**0.9836 ± 0.0013**

Supplementary Table 36: **Impact of mask ratio and elimination strategies on patient-level accuracy and AUROC of CONCH-SNGP without retraining regarding the TCGA and CPTAC test data**. In the following supplementary material, unless otherwise specified, the spectral normalization factor c of CONCH-SNGP is fixed at 2.0. Best-performing model for each case is highlighted in bold, and results are presented with mean ± 95% CI from 20 experimental replicates.

Evaluation dataset	Mask Ratio	Average set size	
		$\alpha = 0.05$	$\alpha = 0.01$
TCGA	0.001	1.1279 ± 0.0029	1.5732 ± 0.0040
TCGA	0.01	1.0907 ± 0.0023	1.5574 ± 0.0040
TCGA	0.1	1.0727 ± 0.0020	1.5429 ± 0.0040
TCGA	0.2	1.0718 ± 0.0020	1.5378 ± 0.0041
TCGA	0.3	1.0693 ± 0.0019	1.5376 ± 0.0042
TCGA	0.4	1.0692 ± 0.0019	1.5383 ± 0.0042
TCGA	0.5	**1.0690 ± 0.0018**	1.5389 ± 0.0042
TCGA	0.6	1.0691 ± 0.0018	1.5398 ± 0.0042
TCGA	0.7	1.0695 ± 0.0018	1.5381 ± 0.0042
TCGA	0.8	1.0696 ± 0.0017	1.5366 ± 0.0042
TCGA	0.9	1.0709 ± 0.0017	**1.5355 ± 0.0042**
TCGA	No mask	1.0742 ± 0.0017	1.5360 ± 0.0042
CPTAC	0.001	1.1726 ± 0.0021	1.4979 ± 0.0042
CPTAC	0.01	1.1233 ± 0.0027	1.5283 ± 0.0045
CPTAC	0.1	1.0686 ± 0.0018	1.4896 ± 0.0043
CPTAC	0.2	1.0628 ± 0.0018	1.4843 ± 0.0043
CPTAC	0.3	1.0552 ± 0.0018	1.4820 ± 0.0043
CPTAC	0.4	1.0525 ± 0.0018	1.4812 ± 0.0043
CPTAC	0.5	1.0516 ± 0.0018	1.4781 ± 0.0043
CPTAC	0.6	**1.0509 ± 0.0018**	**1.4768 ± 0.0042**
CPTAC	0.7	1.0517 ± 0.0018	1.4810 ± 0.0042
CPTAC	0.8	1.0522 ± 0.0018	1.4820 ± 0.0042
CPTAC	0.9	1.0534 ± 0.0018	1.4829 ± 0.0042
CPTAC	No mask	1.0574 ± 0.0019	1.4870 ± 0.0041

Supplementary Table 37: **Impact of mask ratio on the average set size of UNI-SNGP-EAT without retraining (implemented with the label assignment entropy-based EAT) regarding the TCGA and CPTAC test data**. As the coverage for UNI-SNGP-EAT and CONCH-SNGP-EAT is guaranteed, we have omitted reporting their empirical coverage values. Best-performing model for each case is highlighted in bold, and results are presented with mean ± 95% CI from 20 experimental replicates with each using 500 randomly selected calibration/test permutations.

Evaluation dataset	Mask Ratio	Average set size	
		$\alpha = 0.05$	$\alpha = 0.01$
TCGA	0.001	1.1216 ± 0.0026	1.5752 ± 0.0044
	0.01	1.0986 ± 0.0022	1.5443 ± 0.0045
	0.1	1.0923 ± 0.0019	1.5351 ± 0.0048
	0.2	**1.0913 ± 0.0018**	1.5251 ± 0.0048
	0.3	**1.0913 ± 0.0017**	1.5148 ± 0.0047
	0.4	1.0916 ± 0.0017	1.5085 ± 0.0047
	0.5	1.0932 ± 0.0017	**1.5051 ± 0.0047**
	0.6	1.0953 ± 0.0017	1.5058 ± 0.0047
	0.7	1.0970 ± 0.0017	1.5062 ± 0.0048
	0.8	1.0992 ± 0.0017	1.5072 ± 0.0048
	0.9	1.1026 ± 0.0017	1.5074 ± 0.0048
TCGA	No mask	1.1068 ± 0.0016	1.5088 ± 0.0048
CPTAC	0.001	1.0488 ± 0.0017	1.7683 ± 0.0057
	0.01	1.0337 ± 0.0014	1.6600 ± 0.0051
	0.1	1.0296 ± 0.0011	1.5894 ± 0.0049
	0.2	1.0257 ± 0.0010	1.5424 ± 0.0046
	0.3	1.0226 ± 0.0009	1.5210 ± 0.0046
	0.4	1.0203 ± 0.0009	1.5152 ± 0.0046
	0.5	1.0188 ± 0.0008	1.5143 ± 0.0046
	0.6	1.0170 ± 0.0008	1.5112 ± 0.0046
	0.7	1.0158 ± 0.0008	1.5112 ± 0.0046
	0.8	**1.0155 ± 0.0008**	1.5113 ± 0.0045
	0.9	1.0160 ± 0.0007	**1.5098 ± 0.0045**
CPTAC	No mask	1.0174 ± 0.0008	1.5156 ± 0.0045

Supplementary Table 38: **Impact of mask ratio on the average set size of CONCH-SNGP-EAT without retraining (implemented with the label assignment entropy-based EAT) regarding the TCGA and CPTAC test data**. Best-performing model for each case is highlighted in bold, and results are presented with mean ± 95% CI from 20 experimental replicates with each using 500 randomly selected calibration/test permutations.

Model	Metric	Not reported	Others	White	Fairness gap
UNI	Accuracy	0.9467 ± 0.0158	0.8722 ± 0.0264	0.9352 ± 0.0066	0.0891 ± 0.0264
UNI-SNGP	Accuracy	0.9560 ± 0.0163	0.9006 ± 0.0249	0.9379 ± 0.0080	**0.0759 ± 0.0218**
UNI-SNGP-RE	Accuracy	0.9558 ± 0.0146	0.8888 ± 0.0233	0.9368 ± 0.0086	0.0790 ± 0.0235
UNI-SNGP-EAT	Accuracy	0.9555 ± 0.0138	0.8985 ± 0.0257	0.9391 ± 0.0081	0.0774 ± 0.0234
UNI	AUROC	0.9710 ± 0.0103	0.9323 ± 0.0271	0.9813 ± 0.0037	0.0610 ± 0.0254
UNI-SNGP	AUROC	0.9590 ± 0.0162	0.9469 ± 0.0183	0.9800 ± 0.0042	0.0595 ± 0.0162
UNI-SNGP-RE	AUROC	0.9593 ± 0.0155	0.9476 ± 0.0188	0.9800 ± 0.0040	**0.0589 ± 0.0165**
UNI-SNGP-EAT	AUROC	0.9580 ± 0.0165	0.9512 ± 0.0181	0.9799 ± 0.0043	0.0590 ± 0.0155
CONCH	Accuracy	0.9220 ± 0.0269	0.8654 ± 0.0246	0.9297 ± 0.0099	0.0949 ± 0.0257
CONCH-SNGP	Accuracy	0.9249 ± 0.0235	0.8779 ± 0.0264	0.9274 ± 0.0112	**0.0799 ± 0.0229**
CONCH-SNGP-RE	Accuracy	0.9232 ± 0.0241	0.8729 ± 0.0288	0.9285 ± 0.0118	0.0903 ± 0.0250
CONCH-SNGP-EAT	Accuracy	0.9321 ± 0.0227	0.8782 ± 0.0271	0.9327 ± 0.0091	0.0813 ± 0.0251
CONCH	AUROC	0.9584 ± 0.0141	0.9470 ± 0.0192	0.9806 ± 0.0047	0.0552 ± 0.0180
CONCH-SNGP	AUROC	0.9608 ± 0.0122	0.9516 ± 0.0165	0.9826 ± 0.0044	0.0507 ± 0.0153
CONCH-SNGP-RE	AUROC	0.9605 ± 0.0138	0.9502 ± 0.0173	0.9818 ± 0.0048	0.0525 ± 0.0166
CONCH-SNGP-EAT	AUROC	0.9661 ± 0.0115	0.9502 ± 0.0183	0.9836 ± 0.0042	**0.0496 ± 0.0176**

Supplementary Table 39: **Patient-level race-wise fairness gap (Not reported (n=36), Others (n=20), White (n=133)) of UNI, CONCH and their variants with respect to the TCGA test data.** Note that race categories with fewer than 20 patients are aggregated as 'Others'. Best-performing model for each case is highlighted in bold, and results are presented with mean ± 95% CI from 20 experimental replicates.

Model	Metric	Asian	Others	White	Fairness gap
UNI	Accuracy	1.0000 ± 0.0000	0.9000 ± 0.0000	0.9211 ± 0.0052	0.1000 ± 0.0000
UNI-SNGP	Accuracy	1.0000 ± 0.0000	0.8950 ± 0.0102	0.9222 ± 0.0070	0.1061 ± 0.0102
UNI-SNGP-RE	Accuracy	0.9983 ± 0.0035	0.9000 ± 0.0000	0.9200 ± 0.0061	**0.0988 ± 0.0037**
UNI-SNGP-EAT	Accuracy	1.0000 ± 0.0000	0.9000 ± 0.0000	0.9233 ± 0.0061	0.1006 ± 0.0011
UNI	AUROC	1.0000 ± 0.0000	0.9792 ± 0.0097	0.9568 ± 0.0017	**0.0434 ± 0.0016**
UNI-SNGP	AUROC	1.0000 ± 0.0000	0.9708 ± 0.0139	0.9578 ± 0.0035	0.0504 ± 0.0071
UNI-SNGP-RE	AUROC	1.0000 ± 0.0000	0.9562 ± 0.0157	0.9591 ± 0.0026	0.0576 ± 0.0090
UNI-SNGP-EAT	AUROC	1.0000 ± 0.0000	0.9729 ± 0.0127	0.9582 ± 0.0040	0.0483 ± 0.0063
CONCH	Accuracy	0.9966 ± 0.0048	0.9000 ± 0.0000	0.9267 ± 0.0048	**0.0966 ± 0.0048**
CONCH-SNGP	Accuracy	1.0000 ± 0.0000	0.9000 ± 0.0000	0.9267 ± 0.0048	0.1000 ± 0.0000
CONCH-SNGP-RE	Accuracy	1.0000 ± 0.0000	0.9000 ± 0.0000	0.9278 ± 0.0065	0.1006 ± 0.0011
CONCH-SNGP-EAT	Accuracy	1.0000 ± 0.0000	0.9000 ± 0.0000	0.9333 ± 0.0000	0.1000 ± 0.0000
CONCH	AUROC	1.0000 ± 0.0000	0.9156 ± 0.0176	0.9568 ± 0.0029	0.0844 ± 0.0176
CONCH-SNGP	AUROC	1.0000 ± 0.0000	0.9604 ± 0.0043	0.9642 ± 0.0010	0.0415 ± 0.0003
CONCH-SNGP-RE	AUROC	1.0000 ± 0.0000	0.9604 ± 0.0043	0.9654 ± 0.0017	**0.0414 ± 0.0005**
CONCH-SNGP-EAT	AUROC	1.0000 ± 0.0000	0.9583 ± 0.0000	0.9606 ± 0.0011	0.0420 ± 0.0007

Supplementary Table 40: **Patient-level race-wise fairness gap (Asian (n=29), Others (n=10), White (n=45)) of UNI, CONCH and their variants with respect to the CPTAC test data.** Best-performing model for each case is highlighted in bold, and results are presented with mean ± 95% CI from 20 experimental replicates.

Model	Metric	Female	Male	Fairness gap
UNI	Accuracy	0.9244 ± 0.0102	0.9346 ± 0.0067	**0.0244 ± 0.0064**
UNI-SNGP	Accuracy	0.9472 ± 0.0126	0.9324 ± 0.0082	0.0281 ± 0.0107
UNI-SNGP-RE	Accuracy	0.9475 ± 0.0130	0.9291 ± 0.0085	0.0326 ± 0.0095
UNI-SNGP-EAT	Accuracy	0.9473 ± 0.0125	0.9331 ± 0.0078	0.0294 ± 0.0093
UNI	AUROC	0.9783 ± 0.0060	0.9696 ± 0.0058	0.0165 ± 0.0062
UNI-SNGP	AUROC	0.9773 ± 0.0066	0.9693 ± 0.0041	0.0131 ± 0.0059
UNI-SNGP-RE	AUROC	0.9774 ± 0.0066	0.9694 ± 0.0039	0.0137 ± 0.0056
UNI-SNGP-EAT	AUROC	0.9777 ± 0.0066	0.9699 ± 0.0039	**0.0125 ± 0.0058**
CONCH	Accuracy	0.9207 ± 0.0141	0.9220 ± 0.0067	0.0228 ± 0.0070
CONCH-SNGP	Accuracy	0.9229 ± 0.0150	0.9214 ± 0.0090	0.0242 ± 0.0118
CONCH-SNGP-RE	Accuracy	0.9267 ± 0.0148	0.9193 ± 0.0102	**0.0221 ± 0.0124**
CONCH-SNGP-EAT	Accuracy	0.9266 ± 0.0133	0.9269 ± 0.0077	0.0262 ± 0.0080
CONCH	AUROC	0.9772 ± 0.0051	0.9683 ± 0.0060	0.0135 ± 0.0047
CONCH-SNGP	AUROC	0.9793 ± 0.0058	0.9699 ± 0.0052	0.0131 ± 0.0050
CONCH-SNGP-RE	AUROC	0.9779 ± 0.0065	0.9695 ± 0.0054	**0.0130 ± 0.0052**
CONCH-SNGP-EAT	AUROC	0.9811 ± 0.0059	0.9714 ± 0.0049	0.0138 ± 0.0046

Supplementary Table 41: **Patient-level sex-wise fairness gap among different groups (Female (n=68), Male (n=121)) of UNI, CONCH and their variants with respect to the TCGA test data.** Best-performing model for each case is highlighted in bold, and results are presented with mean ± 95% CI from 20 experimental replicates.

Model	Metric	Female	Male	Fairness gap
UNI	Accuracy	0.9259 ± 0.0000	0.9553 ± 0.0041	**0.0293 ± 0.0041**
UNI-SNGP	Accuracy	0.9167 ± 0.0075	0.9596 ± 0.0037	0.0430 ± 0.0080
UNI-SNGP-RE	Accuracy	0.9148 ± 0.0080	0.9588 ± 0.0039	0.0440 ± 0.0097
UNI-SNGP-EAT	Accuracy	0.9167 ± 0.0075	0.9614 ± 0.0033	0.0447 ± 0.0082
UNI	AUROC	0.9671 ± 0.0000	0.9760 ± 0.0008	**0.0088 ± 0.0008**
UNI-SNGP	AUROC	0.9622 ± 0.0027	0.9805 ± 0.0019	0.0184 ± 0.0036
UNI-SNGP-RE	AUROC	0.9625 ± 0.0024	0.9805 ± 0.0020	0.0180 ± 0.0035
UNI-SNGP-EAT	AUROC	0.9599 ± 0.0052	0.9808 ± 0.0021	0.0210 ± 0.0061
CONCH	Accuracy	0.9222 ± 0.0052	0.9596 ± 0.0037	0.0374 ± 0.0071
CONCH-SNGP	Accuracy	0.9259 ± 0.0000	0.9596 ± 0.0037	0.0337 ± 0.0037
CONCH-SNGP-RE	Accuracy	0.9278 ± 0.0038	0.9596 ± 0.0046	**0.0319 ± 0.0056**
CONCH-SNGP-EAT	Accuracy	0.9259 ± 0.0000	0.9649 ± 0.0000	0.0390 ± 0.0000
CONCH	AUROC	0.9862 ± 0.0029	0.9619 ± 0.0066	0.0242 ± 0.0040
CONCH-SNGP	AUROC	0.9934 ± 0.0000	0.9786 ± 0.0022	0.0148 ± 0.0022
CONCH-SNGP-RE	AUROC	0.9934 ± 0.0000	0.9788 ± 0.0022	0.0146 ± 0.0022
CONCH-SNGP-EAT	AUROC	0.9872 ± 0.0012	0.9792 ± 0.0022	**0.0080 ± 0.0017**

Supplementary Table 42: **Patient-level sex-wise fairness gap (Female (n=27), Male (n=57)) of UNI, CONCH and their variants with respect to the CPTAC test data.** Best-performing model for each case is highlighted in bold, and results are presented with mean ± 95% CI from 20 experimental replicates.

Model	α	Average set size across racial groups			
		Not reported	Others	White	Fairness gap
UNI	0.05	1.0557 ± 0.0018	1.0984 ± 0.0028	1.0907 ± 0.0017	0.1128 ± 0.0017
UNI-SNGP	0.05	1.0446 ± 0.0014	1.0891 ± 0.0028	1.0807 ± 0.0018	0.1044 ± 0.0018
UNI-SNGP-RE	0.05	1.0441 ± 0.0014	1.0912 ± 0.0027	1.0853 ± 0.0018	0.1070 ± 0.0018
UNI-SNGP-EAT	0.05	1.0355 ± 0.0014	1.0811 ± 0.0028	1.0772 ± 0.0020	**0.1006 ± 0.0019**
UNI	0.01	1.5616 ± 0.0060	1.6155 ± 0.0051	1.5731 ± 0.0048	**0.1809 ± 0.0022**
UNI-SNGP	0.01	1.4562 ± 0.0054	1.5601 ± 0.0046	1.5529 ± 0.0041	0.2304 ± 0.0029
UNI-SNGP-RE	0.01	1.4498 ± 0.0053	1.5518 ± 0.0046	1.5580 ± 0.0041	0.2348 ± 0.0029
UNI-SNGP-EAT	0.01	1.4480 ± 0.0055	1.5603 ± 0.0046	1.5588 ± 0.0041	0.2355 ± 0.0029
CONCH	0.05	1.0821 ± 0.0019	1.1362 ± 0.0028	1.1218 ± 0.0017	0.1308 ± 0.0018
CONCH-SNGP	0.05	1.0776 ± 0.0019	1.1266 ± 0.0026	1.1116 ± 0.0017	0.1225 ± 0.0017
CONCH-SNGP-RE	0.05	1.0833 ± 0.0019	1.1368 ± 0.0027	1.1178 ± 0.0018	0.1262 ± 0.0017
CONCH-SNGP-EAT	0.05	1.0536 ± 0.0018	1.1121 ± 0.0026	1.0989 ± 0.0018	**0.1158 ± 0.0017**
CONCH	0.01	1.5017 ± 0.0058	1.5811 ± 0.0054	1.5551 ± 0.0051	**0.1740 ± 0.0021**
CONCH-SNGP	0.01	1.4547 ± 0.0052	1.5358 ± 0.0051	1.5193 ± 0.0049	0.1780 ± 0.0021
CONCH-SNGP-RE	0.01	1.4580 ± 0.0052	1.5380 ± 0.0051	1.5223 ± 0.0049	0.1782 ± 0.0021
CONCH-SNGP-EAT	0.01	1.4380 ± 0.0053	1.5466 ± 0.0051	1.5218 ± 0.0047	0.1935 ± 0.0022

Supplementary Table 43: **Patient-level race-wise fairness gap on average set size (Not reported (n=36), Others (n=20), White (n=133)) of UNI, CONCH and their variants with respect to the internal TCGA test data**. Best-performing model for fairness gap is highlighted in bold, and results are presented with mean ± 95% confidence interval (CI) from 20 experimental replicates.

Model	α	Average set size across racial groups			
		Asian	Others	White	Fairness gap
UNI	0.05	1.0319 ± 0.0009	1.1053 ± 0.0031	1.0633 ± 0.0015	0.1109 ± 0.0023
UNI-SNGP	0.05	1.0389 ± 0.0022	1.0809 ± 0.0030	1.0632 ± 0.0018	0.0991 ± 0.0023
UNI-SNGP-RE	0.05	1.0404 ± 0.0022	1.0940 ± 0.0033	1.0659 ± 0.0018	0.1072 ± 0.0025
UNI-SNGP-EAT	0.05	1.0317 ± 0.0022	1.0738 ± 0.0028	1.0600 ± 0.0018	**0.0962 ± 0.0022**
UNI	0.01	1.4113 ± 0.0029	1.5941 ± 0.0037	1.4413 ± 0.0031	**0.2298 ± 0.0023**
UNI-SNGP	0.01	1.3752 ± 0.0058	1.7105 ± 0.0041	1.5027 ± 0.0040	0.3984 ± 0.0032
UNI-SNGP-RE	0.01	1.3607 ± 0.0058	1.6896 ± 0.0044	1.4849 ± 0.0040	0.3934 ± 0.0032
UNI-SNGP-EAT	0.01	1.3294 ± 0.0062	1.7195 ± 0.0042	1.5160 ± 0.0042	0.4443 ± 0.0037
CONCH	0.05	1.0295 ± 0.0014	1.0077 ± 0.0009	1.0333 ± 0.0014	0.0592 ± 0.0011
CONCH-SNGP	0.05	1.0088 ± 0.0007	1.0005 ± 0.0002	1.0258 ± 0.0011	**0.0490 ± 0.0008**
CONCH-SNGP-RE	0.05	1.0083 ± 0.0007	1.0014 ± 0.0003	1.0256 ± 0.0011	0.0497 ± 0.0008
CONCH-SNGP-EAT	0.05	1.0030 ± 0.0004	1.0276 ± 0.0013	1.0291 ± 0.0013	0.0571 ± 0.0012
CONCH	0.01	1.6627 ± 0.0047	1.6311 ± 0.0049	1.6675 ± 0.0046	0.1743 ± 0.0019
CONCH-SNGP	0.01	1.5386 ± 0.0052	1.5125 ± 0.0051	1.5035 ± 0.0043	**0.1632 ± 0.0019**
CONCH-SNGP-RE	0.01	1.5378 ± 0.0052	1.5101 ± 0.0051	1.4996 ± 0.0043	0.1638 ± 0.0019
CONCH-SNGP-EAT	0.01	1.5184 ± 0.0056	1.4934 ± 0.0047	1.5180 ± 0.0043	0.1708 ± 0.0019

Supplementary Table 44: **Patient-level race-wise fairness gap on average set size (Asian (n=29), Others (n=10), White (n=45)) of UNI, CONCH and their variants with respect to the CPTAC test data**. Best-performing model for fairness is highlighted in bold, and results are presented with mean ± 95% confidence interval (CI) from 20 experimental replicates.

Model	α	Average set size across sexual groups		
		Female	Male	Fairness gap
UNI	0.05	1.0951 ± 0.0018	1.0781 ± 0.0018	0.0475 ± 0.0009
UNI-SNGP	0.05	1.0886 ± 0.0022	1.0660 ± 0.0015	0.0462 ± 0.0010
UNI-SNGP-RE	0.05	1.0911 ± 0.0021	1.0699 ± 0.0015	0.0470 ± 0.0010
UNI-SNGP-EAT	0.05	1.0846 ± 0.0024	1.0606 ± 0.0016	**0.0457 ± 0.0011**
UNI	0.01	1.5794 ± 0.0047	1.5740 ± 0.0054	0.1151 ± 0.0016
UNI-SNGP	0.01	1.6175 ± 0.0043	1.4894 ± 0.0045	**0.1658 ± 0.0023**
UNI-SNGP-RE	0.01	1.6200 ± 0.0043	1.4907 ± 0.0044	0.1691 ± 0.0023
UNI-SNGP-EAT	0.01	1.6288 ± 0.0042	1.4868 ± 0.0045	0.1786 ± 0.0022
CONCH	0.05	1.1296 ± 0.0022	1.1077 ± 0.0015	0.0591 ± 0.0011
CONCH-SNGP	0.05	1.1189 ± 0.0023	1.0996 ± 0.0015	0.0576 ± 0.0011
CONCH-SNGP-RE	0.05	1.1242 ± 0.0023	1.1066 ± 0.0015	0.0583 ± 0.0011
CONCH-SNGP-EAT	0.05	1.1039 ± 0.0024	1.0847 ± 0.0015	**0.0545 ± 0.0012**
CONCH	0.01	1.5856 ± 0.0053	1.5265 ± 0.0053	0.1024 ± 0.0016
CONCH-SNGP	0.01	1.5497 ± 0.0051	1.4856 ± 0.0048	0.1024 ± 0.0016
CONCH-SNGP-RE	0.01	1.5549 ± 0.0051	1.4873 ± 0.0049	**0.1022 ± 0.0016**
CONCH-SNGP-EAT	0.01	1.5668 ± 0.0050	1.4757 ± 0.0048	0.1150 ± 0.0017

Supplementary Table 45: **Patient-level sex-wise fairness gap on average set size (Female (n=68), Male (n=121)) of UNI, CONCH and their variants with respect to the TCGA test data**. Best-performing model for fairness gap is highlighted in bold, and results are presented with mean ± 95% CI from 20 experimental replicates.

Model	α	Average set size across sexual groups		
		Female	Male	Fairness gap
UNI	0.05	1.0660 ± 0.0016	1.0541 ± 0.0014	**0.0417 ± 0.0007**
UNI-SNGP	0.05	1.0783 ± 0.0026	1.0469 ± 0.0016	0.0507 ± 0.0011
UNI-SNGP-RE	0.05	1.0788 ± 0.0025	1.0520 ± 0.0017	0.0482 ± 0.0011
UNI-SNGP-EAT	0.05	1.0726 ± 0.0025	1.0425 ± 0.0015	0.0561 ± 0.0011
UNI	0.01	1.3984 ± 0.0036	1.4755 ± 0.0028	**0.1252 ± 0.0017**
UNI-SNGP	0.01	1.4711 ± 0.0051	1.4948 ± 0.0040	0.1375 ± 0.0020
UNI-SNGP-RE	0.01	1.4437 ± 0.0049	1.4829 ± 0.0041	0.1304 ± 0.0019
UNI-SNGP-EAT	0.01	1.4914 ± 0.0050	1.4758 ± 0.0042	0.1296 ± 0.0018
CONCH	0.05	1.0255 ± 0.0014	1.0310 ± 0.0013	0.0351 ± 0.0007
CONCH-SNGP	0.05	1.0084 ± 0.0007	1.0218 ± 0.0009	0.0273 ± 0.0005
CONCH-SNGP-RE	0.05	1.0079 ± 0.0008	1.0218 ± 0.0009	0.0286 ± 0.0005
CONCH-SNGP-EAT	0.05	1.0137 ± 0.0008	1.0235 ± 0.0010	**0.0259 ± 0.0006**
CONCH	0.01	1.6261 ± 0.0046	1.6796 ± 0.0047	0.1264 ± 0.0015
CONCH-SNGP	0.01	1.5042 ± 0.0053	1.5213 ± 0.0043	0.1070 ± 0.0015
CONCH-SNGP-RE	0.01	1.4945 ± 0.0052	1.5221 ± 0.0044	**0.1049 ± 0.0015**
CONCH-SNGP-EAT	0.01	1.5172 ± 0.0055	1.5142 ± 0.0043	0.1184 ± 0.0017

Supplementary Table 46: **Patient-level sex-wise performance fairness gap on average set size (Female (n=27), Male (n=57)) of UNI, CONCH and their variants with respect to the CPTAC test data**. Best-performing model for fairness gap is highlighted in bold, and results are presented with mean ± 95% CI from 20 experimental replicates with 500 calibration/test permutations.

Dataset	Link
TCGA-NSCLC	https://portal.gdc.cancer.gov/projects/TCGA-LUAD<br>https://portal.gdc.cancer.gov/projects/TCGA-LUSC
CPTAC-NSCLC	https://portal.gdc.cancer.gov/projects/CPTAC-3
GTEx	https://www.gtexportal.org/home/histologyPage
TCGA-OT	https://portal.gdc.cancer.gov
TCGA-BRTS	https://portal.gdc.cancer.gov
BRACS	https://bracs.icar.cnr.it
TCGA-BRCA	https://portal.gdc.cancer.gov/projects/TCGA-BRCA
TCGA-RCC	https://portal.gdc.cancer.gov/projects/TCGA-RCC

Supplementary Table 47: **Summary of publicly available datasets used in the paper.**
